# Intermittent fasting for microbes: how discontinuous feeding increases functional stability in anaerobic digestion

**DOI:** 10.1186/s13068-018-1279-5

**Published:** 2018-10-06

**Authors:** Fabian Bonk, Denny Popp, Sören Weinrich, Heike Sträuber, Sabine Kleinsteuber, Hauke Harms, Florian Centler

**Affiliations:** 1 Department of Environmental Microbiology, UFZ–Helmholtz Centre for Environmental Research, Permoserstr. 15, 04318 Leipzig, Germany; 2 Biochemical Conversion Department, DBFZ Deutsches Biomasseforschungszentrum Gemeinnützige GmbH, Torgauer Str. 116, 04347 Leipzig, Germany

**Keywords:** Biogas, Microbial resources management, ADM1, Acetoclastic methanogenesis

## Abstract

**Background:**

Demand-driven biogas production could play an important role for future sustainable energy supply. However, feeding a biogas reactor according to energy demand may lead to organic overloading and, thus, to process failures. To minimize this risk, digesters need to be actively steered towards containing more robust microbial communities. This study focuses on acetogenesis and methanogenesis as crucial process steps for avoiding acidification. We fed lab-scale anaerobic digesters with volatile fatty acids under various feeding regimes and disturbances. The resulting microbial communities were analyzed on DNA and RNA level by terminal restriction fragment length polymorphism of the *mcrA* gene, 16S rRNA gene amplicon sequencing, and a [2-^13^C]-acetate assay. A modified Anaerobic Digestion Model 1 (ADM1) that distinguishes between the acetoclastic methanogens *Methanosaeta* and *Methanosarcina* was developed and fitted using experimental abiotic and biotic process parameters.

**Results:**

Discontinuous feeding led to more functional resilience than continuous feeding, without loss in process efficiency. This was attributed to a different microbial community composition. *Methanosaeta* dominated the continuously fed reactors, while its competitor *Methanosarcina* was washed out. With discontinuous feeding, however, the fluctuating acetic acid concentrations provided niches to grow and co-exist for both organisms as shown by transcription analysis of the *mcrA* gene. Our model confirmed the higher functional resilience due to the higher abundance of *Methanosarcina* based on its higher substrate uptake rate and higher resistance to low pH values. Finally, we applied our model to maize silage as a more complex and practically relevant substrate and showed that our model is likely transferable to the complete AD process.

**Conclusions:**

The composition of the microbial community determined the AD functional resilience against organic overloading in our experiments. In particular, communities with higher share of *Methanosarcina* showed higher process stability. The share of these microorganisms can be purposefully increased by discontinuous feeding. A model was developed that enables derivation of the necessary feeding regime for a more robust community with higher share of *Methanosarcina*.

**Electronic supplementary material:**

The online version of this article (10.1186/s13068-018-1279-5) contains supplementary material, which is available to authorized users.

## Background

Demand-driven electricity production from biogas could be used in the future to compensate for supply shortages associated with fluctuating renewable energies like wind and solar power [1, 2]. To avoid large, expensive biogas storage modules, biogas needs to be produced flexibly by changing the organic loading rate of anaerobic digesters [3]. However, this can result in accidental organic overloading, i.e., organic loads that exceed the capacity of acetogenesis and methanogenesis, thus leading to accumulation of volatile fatty acids (VFAs) and potentially to process failure [4, 5]. One option to avoid process failures is increasing the microbial community’s functional resilience, i.e., its ability to quickly return to its previous methane production rate, pH and VFA concentrations following disturbance.

Selecting a microbial community that ensures higher functional resilience requires a microbial resources management strategy. Such a strategy necessitates monitoring of the microbial community which can be achieved by molecular biological analyses [6]. These can be based on marker genes, such as the 16S rRNA gene [6], but techniques targeting all genomes (metagenome) [7–9], all proteins (metaproteome) [10], all transcripts (metatranscriptome) [11] or all metabolites (metabolome) [12] in a sample have also been applied in anaerobic digestion research. For simply monitoring the change in relative abundance of microbial taxa, amplicon sequencing and terminal restriction fragment length polymorphism (T-RFLP) analysis of marker genes are more cost efficient than metagenome analyses. Beyond mere monitoring, microbial resources management requires ways to manipulate the composition of the microbial community. These can be the addition of certain beneficial microorganisms (bioaugmentation) [13] and/or the application of selection pressure, i.e., choosing specific process parameters, to favor their growth. Such environmental selection pressures can be, for example, temperature [14, 15], ammonia concentration [16], feed composition [17], hydraulic retention time (HRT) [18], organic loading rate (OLR) [19], or the feeding regime in terms of temporal feeding schedule [1, 20–24]. A temporal feeding schedule is particularly interesting because it could be implemented in industrial practice without additional investments [25].

Continuous feeding has been commonly suggested in industrial practice to assure stable biogas production [3]. Contrary to this practice, several laboratory-scale studies have shown a positive influence of discontinuous feeding regimes on process performance and functional stability associated with shifts in the microbial community [1, 20–23]. In addition to functional stability, a techno-economic analysis for existing co-digestion plants in Belgium revealed that discontinuous feeding regimes could even be economically advantageous as they allowed an increase in overall organic loading rates and biogas production [25].

Discontinuous feeding (once per 24 h or 48 h instead of every 2 h) improved stability against organic overloading and even led to higher process efficiency compared to continuous feeding in a lab-scale experiment [1]. In that study, dried distillers grains with solubles (DDGS) were used as substrate and reactors were operated at 38 °C with an HRT of 10–26 days and OLRs of 4–11 g volatile solids per liter per day. Using T-RFLP analysis, a stable dominance of *Methanosarcina* in the methanogenic communities was observed, while the relative abundances of bacterial terminal restriction fragments (T-RFs) changed as an effect of the feeding regime. However, no taxa were assigned to these T-RFs due to the lack of sequence data.

In another study, discontinuous feeding (every second day compared to once per day) of synthetic raw domestic sewage also led to a higher functional stability against organic overloading and ammonia shocks [20]. The higher stability was attributed to a more dynamic bacterial community as determined by denaturing gradient gel electrophoresis (DGGE). However, from the DGGE analyses, it cannot be concluded if this principle applies to acidogenic and acetogenic bacteria alike. For functional stability against organic overloading, in particular, acetogens and methanogens are important because they determine how quickly the accumulated VFAs are degraded. A closer analysis of acetogens and methanogens is, therefore, needed.

In a study with focus on acetoclastic methanogens [21], an hourly vs. daily feeding of acetic acid led to communities with higher share of *Methanosaeta* and *Methanosarcina*, respectively. The reactors with higher share of *Methanosarcina* showed higher stability against acetic acid overloading, which was attributed to a higher acetate capacity number (ACN). The ACN is the quotient of the maximum acetic acid uptake rate (determined in batch experiments with reactor samples subjected to high acetic acid concentrations) and the currently observed average acetic acid uptake rate [26]. A higher ACN is the result of a higher apparent biomass yield and maximum specific acetic acid uptake rate of *Methanosarcina*. Furthermore, simulations in the Anaerobic Digestion Model No. 1 (ADM1) led to the conclusion that *Methanosaeta* cannot sustain HRTs below 11 days and will be outcompeted at HRTs below 15 days under continuous feeding [26].

**Fig. 1:**
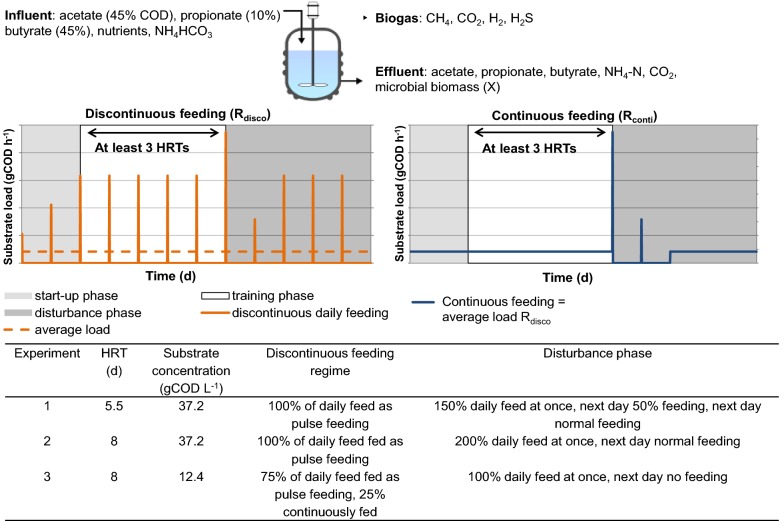
Experimental design. Lab-scale biogas reactors were fed with VFAs using two different feeding regimes (*R*_disco_ and *R*_conti_), two different HRTs and two different substrate concentrations. The microbial community was analyzed based on the 16S rRNA gene (amplicon sequencing) for bacteria and the *mcrA* gene (T-RFLP) for methanogenic archaea. Substrate concentration of 37.2 gCOD L^−1^ corresponds to 27.4 g L^−1^ and 0.54 M

Not only acetoclastic methanogens are the sole determinant for functional resilience, but also syntrophic VFA-oxidizing bacteria as well as hydrogenotrophic methanogens may play important roles. Therefore, in this study, we used a mixture of VFAs as substrate in reactor experiments to test the impact of discontinuous compared to continuous feeding on community composition and functional resilience. Three experiments were conducted to test two different HRTs, three OLRs, and two substrate concentrations (see Fig. 1). The resulting microbial community was analyzed by 16S rRNA gene amplicon sequencing, DNA- and RNA-based T-RFLP profiling of *mcrA* amplicons, and a [2-^13^C]-acetate assay. Abiotic and biotic process parameters were used to develop and fit a modified ADM1 model.

## Results and discussion

### Discontinuous feeding increased functional resilience without loss in efficiency

**Fig. 2:**
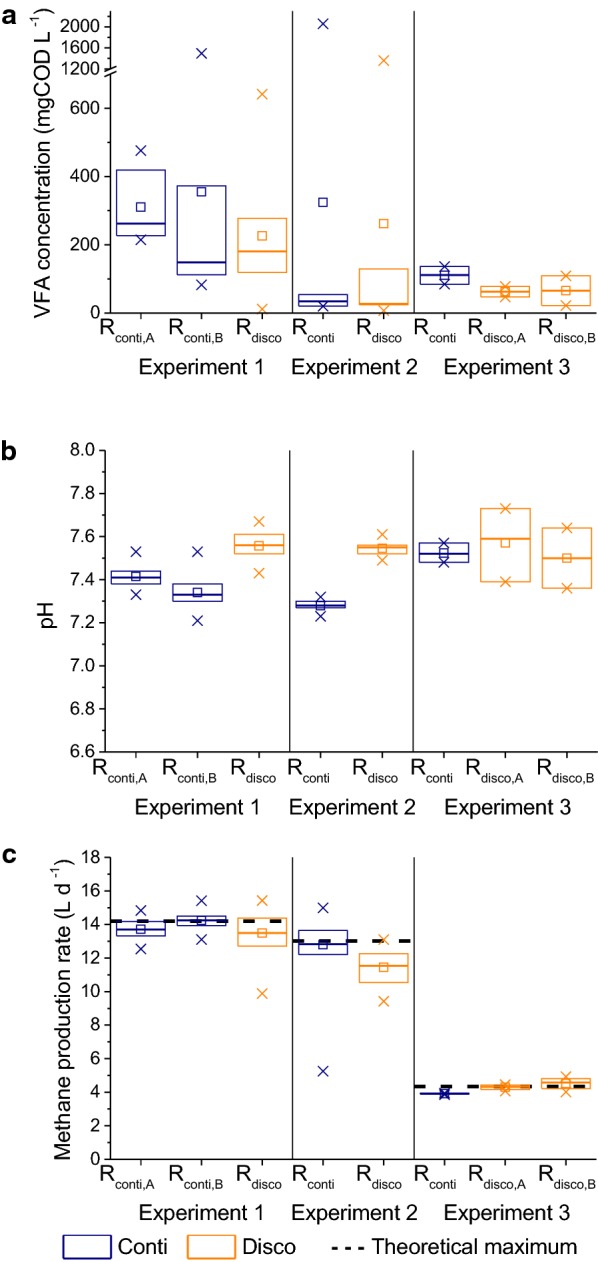
Process performance at the end of the training phase of each experiment. Biological replicates are designated as “A” and “B”. Box plots include all samples from 3 HRTs after the start of the training phase until the end of the training phase. **a** Total VFA concentration, **b** pH, **c** experimental methane production rates and theoretical maximum methane production rate assuming complete conversion of all VFAs to methane. Boxes represent the 25–75% percentiles, solid lines the median, squares the average, x the max and minimum, and dashed lines the theoretical maximum

VFA concentrations in biogas reactors are a critical indicator for process performance. The average VFA concentrations in both the continuously (*R*_conti_) and discontinuously (*R*_disco_) fed reactors in all experiments were below 0.36 g chemical oxygen demand (COD) per liter towards the end of the training phase (see Fig. 2a). Given substrate influent concentrations of 37.2 gCOD L^−1^ for Experiment 1 and Experiment 2 as well as 12.4 gCOD L^−1^ for Experiment 3, the average VFA concentrations in the reactors corresponded to substrate conversion efficiencies of more than 99%. This was reflected by methane production rates close to the theoretical maximum values assuming complete VFA conversion into methane (see Fig. 2c). VFA concentration was the better measure for process performance in our experiment, since biogas composition could be measured only up to twice daily, and thus did not fully reflect the dynamics in the discontinuously fed reactors.

**Fig. 3:**
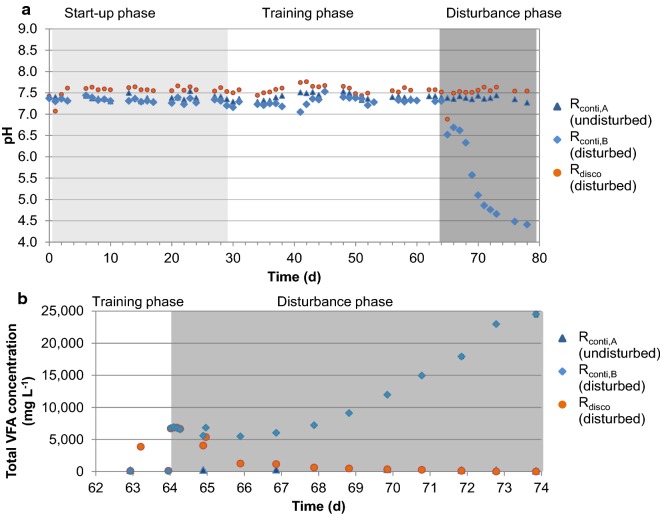
Effect of disturbance in Experiment 1. **a** pH values over the whole course of the experiment and **b** total VFA concentrations in the disturbance phase. Process disturbance led to failure of the continuously fed reactor (*R*_conti,B,disturbed_) but not for the discontinuously fed reactor (*R*_disco,disturbed_) in Experiment 1. The continuously fed undisturbed control reactor (*R*_conti,A_) shows no changes in pH and VFA concentrations

In Experiment 1, the same disturbance (organic overloading) for *R*_conti_ and *R*_disco_ led to process failure of *R*_conti_; while for *R*_disco_, the pre-disturbance pH value was reached after 1 day (see Fig. 3a) and the pre-disturbance total VFA concentration was reached after about 1 week (see Fig. 3b). In Experiment 2, a similar behavior was observed (see Additional file 1, Figure S2). In Experiment 3, there was hardly any effect on either *R*_conti_ or *R*_disco_ because a weaker disturbance was used (see Additional file 1, Figure S3). In conclusion, discontinuous feeding led to a higher functional resilience against organic overloading without negative effects on process efficiency, which is in accordance with the previous findings [1, 20, 21]. In the following, the mechanisms leading to this higher functional resilience are discussed.

### Potential reasons for higher functional resilience

#### Functional resilience not explained by biomass concentration nor pH

The VFA conversion capacity of an anaerobic digester depends on the amount of biocatalysts [27], i.e., the microbial biomass concentration. Therefore, a higher biomass concentration could be the reason for the higher functional resilience of *R*_disco_ in Experiments 1 and 2. However, the microbial biomass concentrations before the disturbance were 1.11 ± 0.0043 gVS L^−1^ for* R*_conti,B_ compared to 0.85 ± 0.0003 gVS L^−1^ for *R*_disco_ in Experiment 1, and 0.87 ± 0.01 gVS L^−1^ (average ± 1 standard error of mean) for *R*_conti_ compared to 0.78 ± 0.02 gVS L^−1^ for *R*_disco_ in Experiment 2 (see Additional file 1, Figure S1, S2). The microbial biomass concentration before the disturbance was lower for *R*_disco_ in Experiments 1 and 2 (*p* < 0.016, Tukey’s test with *α* = 0.05) and, thus, cannot explain the higher functional resilience of *R*_disco_.

Based on abiotic parameters, the only notable difference between *R*_conti_ and *R*_disco_ concerned the pH values, which were about 0.3 points higher for *R*_disco_ compared to *R*_conti_ at the end of the training phase of Experiment 1 (see Fig. 2b) and Experiment 2 (see Additional file 1, Figure S9). However, after the disturbance in Experiment 1, the pH value difference disappeared and the resulting values were almost identical with 5.93 and 5.99 for *R*_conti_ and *R*_disco_, respectively. Hence, right after the disturbance, functional resilience was not a function of pH.

#### Little contribution of bacteria to functional resilience

Ecological indices are a useful tool to describe and compare complex microbial communities [28]. High diversity and/or high evenness are generally expected to have a positive influence on the functionality of microbial communities [28]. However, in Experiment 1 at the end of the training phase, diversity and evenness of the bacterial community were similar in *R*_disco_ and *R*_conti_ and could, therefore, not explain the higher functional resilience of *R*_disco_ (see Additional file 1, Figure S12).

At the end of the training phase, the major phyla in the bacterial communities of all three experiments were Firmicutes (16–57%), Bacteroidetes (4–40%), Synergistetes (4–21%) and Proteobacteria (5–25%) (see Additional file 3). In most samples, *Syntrophobacter* and *Syntrophomonas* belonged to the most abundant genera, which is not surprising given their known roles as propionic and butyric acid oxidizers, respectively. Overall, at the end of the training phase, the bacterial communities of *R*_disco_ of the three experiments could not be clearly differentiated from the communities of *R*_conti_ (see Nonmetric Multidimensional Scaling (NMDS) plot in Additional file 1, S13). Only the operational taxonomic unit (OTU) Lineage I (Endomicrobia), an order within the phylum Elusimicrobia, was found in higher relative abundance in *R*_disco_ compared to *R*_conti_ for each sample. However, relative abundances of this OTU in *R*_disco_ accounted for less than 4.1% of total sequences in all experiments at the end of the training phase (see Additional file 2, Sheet “bacterial indicators”). Therefore, this OTU is most likely not the reason for the higher functional resilience of *R*_disco_. Unfortunately, the functions of this OTU and most other bacterial taxa in our reactors could not be derived from the literature.

Since only VFAs were fed to the reactors, most of the bacteria should be VFA-oxidizing bacteria. At the end of the training phase, 89–107, 83–134, and 86–126 bacterial OTUs were detected in the reactors of Experiments 1, 2 and 3, respectively, but there are only 12 syntrophic VFA oxidizers known in the context of anaerobic digestion from which only three genera were found in our reactors: *Syntrophobacter*, *Pelotomaculum,* and *Syntrophomonas* with a combined relative abundance of 34 ± 13% (average ± 1 SD) for all samples. Some of the other bacterial OTUs might not consume VFAs but decaying microbial biomass. However, based on our model simulations of Experiment 1, biomass degraders should be less than 0.2% of all bacteria, indicating that many VFA-oxidizing bacteria remain to be discovered.

Via mutual exclusions of OTUs with the known VFA oxidizers *Syntrophomonas* (butyric acid oxidizer) and *Syntrophobacter* (propionic acid oxidizer) in a co-occurrence analysis, we inferred Rikenellaceae RC9 gut group, Family XIII UCG-002 (Clostridiales), and *Aminobacterium* as potential propionate-oxidizing bacteria, and Blvii28 wastewater-sludge group, *Desulfovibrio*, *Thermovirga*, *Mesotoga*, and uncultured members of *Syntrophomonadaceae* and *Synergistaceae* as potential butyric acid oxidizing bacteria (see Additional file 1, Section 2.2.5). Because mutual exclusion does not necessarily imply the same function as *Syntrophomonas* and *Syntrophobacter*, these inferences only identify potential candidates. Future studies applying for example meta-omics approaches are required to elucidate the functional roles of these taxa. Furthermore, the phylum Cloacimonetes was found to be highly abundant in several samples. In the genome of *Candidatus* Cloacamonas acidaminovorans, all genes necessary for syntrophic propionate oxidation have been found [29] but no pure or co-culture could be established yet to prove the activity of this pathway.

No hints on syntrophic acetate oxidation were found. Feeding ^13^C-labeled acetate to reactor effluent in batch experiments resulted in ^13^C-labeled CO_2_:^13^C-labeled CH_4_ ratios much lower than 1 (about 0.03), which is an indicator that the major acetic acid utilization pathway was acetoclastic methanogenesis [30] (see Additional file 1, Table S8).

In conclusion, the higher functional resilience of *R*_disco_ could be explained neither by the high abundance of specific bacterial taxa nor by a general change in bacterial diversity or evenness.

#### Strong difference in composition and activity of methanogenic archaea

**Fig. 4:**
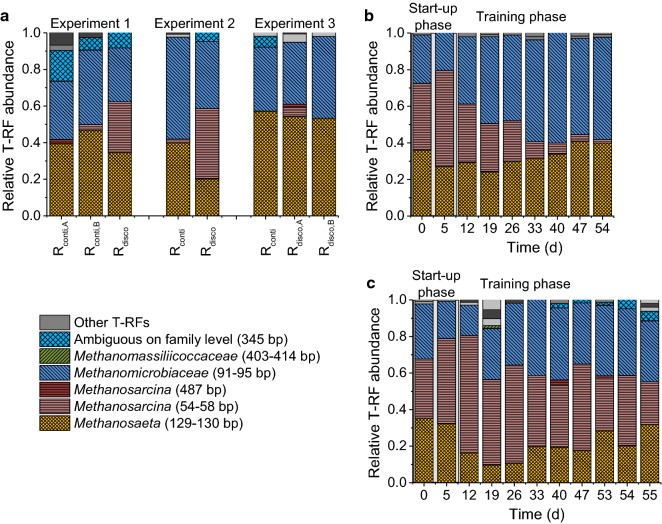
Methanogenic community compositions. T-RFLP analysis of *mcrA* DNA amplicons using *Bst*NI as restriction enzyme **a** at the end of training phase for all experiment as well as time series for **b** *R*_conti_ and **c** *R*_disco_ for Experiment 2. “Ambiguous on family level” stand for *Methanocorpusculaceae*, *Methanomicrobiaceae*, *Methanoregulaceae*, and *Methanospirillaceae*, which cannot be distinguished by this T-RFLP analysis. Other T-RFs are marked in shades of gray

The methanogenic community showed a similar composition for continuous feeding (*R*_conti_) in all experiments at the end of the training phase based on T-RFLP analysis (see Fig. 4a and Additional file 1, Figure S15). The dominating methanogens were the acetoclastic *Methanosaeta* with 40–57% and the hydrogenotrophic *Methanomicrobiaceae* with 35–56% relative abundance. The dominance of *Methanosaeta* under continuous feeding at HRTs of 5.5 and 8 days is remarkable, since it has been assumed previously that *Methanosaeta* will be outcompeted by *Methanosarcina* at HRTs below 15 days [26].

The methanogenic community composition of *R*_disco_ in Experiments 1 and 2 at the end of the training phase strongly differed from that of *R*_conti_. *Methanosaeta* and *Methanomicrobiaceae* were also present but each 10–20 percentage points less abundant than in *R*_conti_. *Methanosarcina* became one of the most abundant taxa in *R*_disco_ with approximately 40% (see Fig. 4a); whereas under continuous feeding, *Methanosarcina* was almost washed out in all experiments, which can be well seen in Experiment 2 (see Fig. 4b). However, under discontinuous feeding, *Methanosarcina* could co-exist and even surpass *Methanosaeta* in abundance (see Fig. 4c). In Experiment 3, the differences in the methanogenic communities between *R*_disco_ and *R*_conti_ were much smaller than in the other experiments, which can be attributed to the lower feeding pulses applied.

**Fig. 5:**
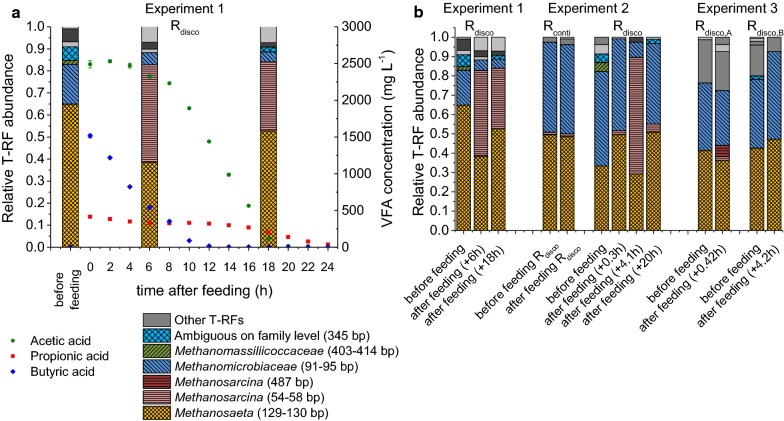
Activity of methanogens. T-RFLP analysis of *mcrA* transcripts (cDNA amplicons) using *Bst*NI as restriction enzyme. **a** Before and after the discontinuous feeding event for Experiment 1 compared to the VFA concentration during a daily feeding interval and **b** *R*_disco_ for all experiments as well as *R*_conti_ as control. “Ambiguous on family level” stands for *Methanocorpusculaceae, Methanomicrobiaceae*, *Methanoregulaceae*, and *Methanospirillaceae*, which cannot be distinguished by this T-RFLP analysis. Other T-RFs are marked in shades of gray

The observed advantage of *Methanosarcina* under discontinuous feeding is in accordance with a previous study, which compared hourly with daily acetic acid feeding [21]. This advantage was explained by the higher maximum substrate uptake rate of *Methanosarcina* at high acetic acid concentrations that follow the discontinuous feeding [21]. We confirmed this phenomenon in our experiments using T-RFLP analysis of *mcrA* transcripts before and after the feeding (see Fig. 5). Before the discontinuous feeding event, acetic acid concentrations were low, and therefore, mostly *Methanosaeta* was active and *Methanosarcina* was not detectable on mRNA level. After the discontinuous feeding, however, acetic acid concentrations were high and thus, *Methanosarcina* became temporarily more active than *Methanosaeta*. In conclusion, *Methanosaeta* was always active, while *Methanosarcina* could only become active at high acetic acid concentrations.

### Reaching increased functional resilience by model-based microbial resources management of methanogens

As discussed above, the differences in pH, total microbial biomass concentration and the bacterial community composition were all unable to explain the increased resilience against organic overloading of *R*_disco_. Therefore, we hypothesized that the increased abundance of *Methanosarcina* with their beneficial physiological properties is the reason for the higher process resilience of *R*_disco_.

A modified ADM1 was used to simulate the two competing acetoclastic methanogens: *Methanosarcina* and *Methanosaeta*. Previously published kinetic growth parameter values for *Methanosaeta* could not be used because they do not support growth at an HRT of 5.5 days [26]. However, in Experiment 1, *Methanosaeta* clearly dominated *R*_conti_ at this HRT. Furthermore, published kinetic growth parameters assume a very high decay rate of 0.1 day^−1^ for *Methanosarcina*, which has been derived from long-term starvation experiments [21] but has never been measured under continuous or discontinuous feeding.

Therefore, new parameter values for the growth kinetics of methanogens were determined. In addition, also the growth kinetic parameters values of acetogens were adjusted to fit our experimental results (see Table 1). Some parameter values could be estimated based on the frequent measurement of VFA concentrations over the course of 24 h for *R*_disco_ in Experiment 1 (see Additional file 1, Section 2.1.4). However, the final parameter set is based on fitting the simulation results to the experimental results concerning total microbial biomass concentration, the VFA concentration and the ratio of *Methanosarcina* and *Methanosaeta* at the end of the training phase as well as the pH drop after the disturbance of both *R*_disco_ and *R*_conti_ in Experiments 1 and 2.

**Table 1:** Model parameters used for acetic, propionic, and butyric acid as well as hydrogen converting populations

	Yield Y (gCOD_X_ gCOD_S_^−1^)	Maximum substrate utilization rate *k*_m_ (gCOD_S_ gCOD_X_^−1^ day^−1^)	Half saturation constant * K* (gCOD_S_ L^−1^)	Decay rate *k*_dec_	pH inhibition	References
X_ac,1_	0.033	20	0.32	0.02	pH_l = 4; pH_u = 5.5	This study
	0.060	8.95	0.32	0.1	N/A	[26]
X_ac,2_	0.033	14.5	0.09	0.02	pH_l = 6.3; pH_u = 7	This study
	0.042	2.77	0.09	0.0064	N/A	[26]
X_h2_	0.032	35	7 × 10^−6^	0.02	pH_l = 5; pH_u = 6	This study
	0.060	35	1 × 10^−6^	0.02	pH_l = 5; pH_u = 6	[52]
X_pro_	0.012	30	0.1	0.02	N/A	This study
	0.040	13	0.1	0.02	N/A	[52]
X_C4_	0.012	25	0.2	0.02	N/A	This study
	0.060	20	0.2	0.02	N/A	[52]

X_ac,1_ = *Methanosarcina*, X_ac,2_ = *Methanosaeta*, X_h2_ = hydrogenotrophic methanogens, X_pro_ = propionic acid degraders, X_C4_ = butyric acid degraders, pH_l = lower limit and pH_u = upper limit for pH inhibition

#### A new parameter set for acetoclastic methanogens

One of the most important findings was that yield and maximum substrate utilization rate values for *Methanosarcina* cannot differ from the values for *Methanosaeta* as much as suggested previously [26]. Otherwise, the co-existence of *Methanosaeta* and *Methanosarcina* in *R*_disco_ in Experiment 1 and Experiment 2 even after several HRTs cannot be achieved in our simulations. A major difference between both genera in our model is the parameter values for pH inhibition resulting in a stronger influence of pH inhibition on *Methanosaeta* due to the higher sensitivity of this genus against low pH values and VFA concentration changes [31].

#### Co-existence of acetoclastic methanogens and higher resilience for *R*_disco_

**Fig. 6:**
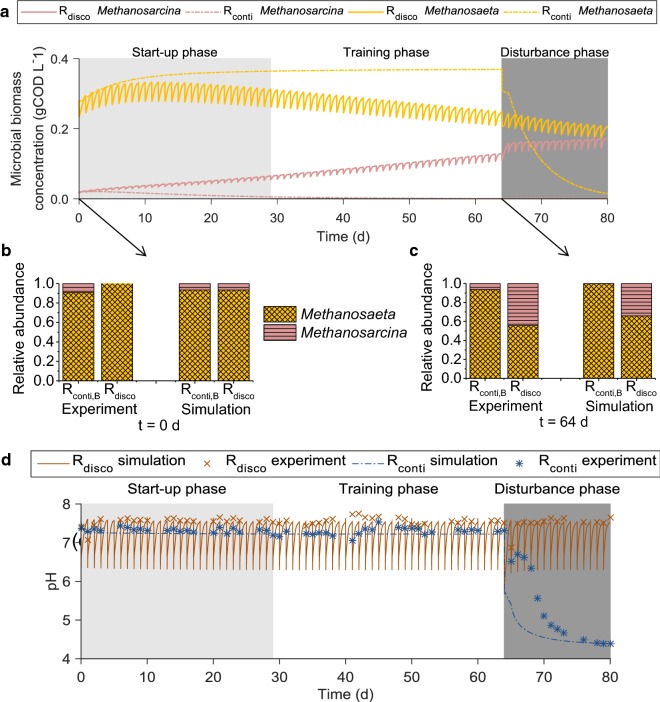
Simulation result of the ADM1 model for Experiment 1. The model includes *Methanosaeta* and *Methanosarcina* extension for R_conti,B_ and *R*_disco_. **a** Acetoclastic methanogens, compared to experimental values, **b** at the start of the experiment and **c** at the end of the training phase, **d** pH values. Disturbance on day 64

Finally, our parameter value set predicted the higher resilience of *R*_disco_ against the disturbance (Fig. 6). Furthermore, our parameter value set was able to predict the dominance of *Methanosaeta* in *R*_conti_ and the co-existence of *Methanosarcina* and *Methanosaeta* at the end of the training phase in *R*_disco_. The relative abundance of *Methanosaeta* among total acetoclastic methanogens at the end of the training phase was 99.8% in *R*_conti_ in the simulation, while it was 93.7% in the T-RFLP analysis. For *R*_disco_, the simulation predicted a relative abundance of *Methanosarcina* of 56% compared to 65% in the T-RFLP analysis at the end of the training phase.

#### Differences in pH between *R*_conti_ and *R*_disco_

The model was also able to explain the higher pH values in *R*_disco_ (Fig. 6). Because all reactors were fed with the same medium, the difference in pH can only be explained by the dynamics of VFA and dissolved inorganic carbon concentrations. The generally higher pH values of *R*_disco_ cannot be explained by residual VFAs because the difference in pH remained even when VFA concentrations were almost 0 in both *R*_disco_ and *R*_conti_ (see Additional file 2, Experiment 2, *t* = 47 days and *t* = 53 days). Therefore, the systematically higher pH seems to be caused by the dynamics of dissolved inorganic carbon concentration in *R*_disco_. During a discontinuous feeding event in Experiment 1, the pH dropped from approximately 7.6 to 6.5 due to the high amount of VFAs suddenly added (see Additional file 1, Figure S4). This shift in pH caused a shift in the acid–base equilibrium from bicarbonate to dissolved CO_2_ resulting in a driving force for a decrease of dissolved inorganic carbon, which caused the higher pH in *R*_disco_. We could show this effect in model simulations for which the dissolved inorganic carbon concentrations were continuously lower with 0.065–0.107 M in *R*_disco_ compared to 0.113 M in *R*_conti_ resulting in maximum pH value of 7.56 for *R*_disco_ versus 7.29 for *R*_conti_. VFA concentrations were negligible in both simulations (< 6 mgCOD L^−1^, see Additional file 1: Figure S21).

#### Limitations and crucial assumptions

The determination of microbial growth yields was constrained by the experimentally determined total microbial biomass concentration. The individual yields could not be unambiguously determined by our DNA-based analyses because the function of all bacterial taxa could not be determined. For example for many bacterial taxa, it was not clear if they belong to propionic or butyric acid oxidizers (see “Little contribution of bacteria to functional resilience” section). Furthermore, DNA-based relative abundances do not necessarily reflect mass or COD-based relative abundances of the bacterial and methanogenic taxa. Inhibition was implemented as default in ADM1, which means that a low pH value is the major contributor to inhibition of acetoclastic methanogens after an increase in VFA concentrations and not direct inhibition by VFAs diffusing into microbial cells.

Most species of *Methanosarcina* can also convert CO_2_ and hydrogen into methane [31] but we assumed *Methanosarcina* to be purely acetoclastic in our model as others did previously for mesophilic continuous stirred tank reactors (CSTRs) [26]. In the T-RFLP analysis, an increase in *Methanosarcina* usually coincided with a decrease in both *Methanosaeta* and hydrogenotrophic methanogens. However, this does not necessarily mean that *Methanosarcina* additionally consumed hydrogen in our reactors because the decrease in relative *mcrA* gene abundance of hydrogenotrophic methanogens could also be a result of a higher microbial COD to *mcrA* gene ratio for *Methanosarcina*. An argument against *Methanosarcina* being additionally a hydrogenotrophic methanogen is its disadvantage against strict hydrogenotrophic methanogens because it requires higher hydrogen partial pressures (> 10 Pa) due to its different energy conservation mechanism [32]. Despite this disadvantage, *Methanosarcina* could, nevertheless, considerably contribute to hydrogen consumption because of its high abundance. This has to be clarified by metatranscriptomics or metaproteomics in future studies.

Furthermore, it should be noted that the substrate composition could have a crucial impact on the outcome of our study. A higher acetic acid concentration in the substrate may require less drastic feeding pulses to provide a niche for *Methanosarcina*. A higher concentration of propionic or butyric acid might lead to niches for other VFA-oxidizing bacteria that were not observed in our experiment. In the fermentation of complex substrates, a variety of products in addition to VFAs can be formed with changing compositions depending on the process conditions [33, 34]. This complexity is a future challenge and a chance to develop feeding regimes as instrument of microbial resources management.

#### Applicability to agricultural biogas plants

We suppose that our feeding regime will not only work for VFAs as substrate but also for many other substrates as long as hydrolysis and acidogenesis are faster than acetogenesis and methanogenesis thus leading to VFA accumulations. To illustrate this, we simulated a maize silage fed reactor using our modified ADM1 model with the same growth kinetic parameter values used for Experiment 1 (see Additional file 1, Figure S24). Under continuous feeding with maize silage, the reactor was completely dominated by *Methanosaeta*. Changing to discontinuous feeding (every second day) led to an increase in the abundance of *Methanosarcina* by one order of magnitude in 1 year. We chose the initial concentration of *Methanosarcina* to 0.2 gCOD L^−1^, which could be easily reached in practice by replacing about 5% of the working volume with effluent from a digester with a high share of *Methanosarcina*. In conclusion, simulations with maize silage suggested that discontinuous feeding could also be used in practice for microbial resources management. However, insufficient substrate and digestate storage capacities necessary for discontinuous feeding might limit the applicability in some settings such as wastewater treatment plants. In other settings, such as agricultural biogas plants, storage capacity may not be limiting.

Further substrates, for which hydrolysis is not the rate limiting step, may be suitable for our proposed microbial resource management strategy, such as chicken manure [35], municipal solid waste co-digested with sewage sludge [36], thin stillage [37], sugar beet by-products co-digested with pig manure [38] and food waste [39]. Lignocellulosic wastes are most likely not suitable without pre-treatment, because of low hydrolysis rates due to their compositional and structural features [40].

## Conclusions and outlook for microbial resources management to increase resilience against organic overloading

Our study shows that a model-based microbial resources management to increase resilience against organic overloading is possible. Concerning methanogens, increasing the relative abundance of *Methanosarcina* at the cost of *Methanosaeta* is recommended. This can be achieved by certain discontinuous feeding regimes that lead to temporary acetic acid accumulations and pH drops to provide a niche for *Methanosarcina* (active only at higher acetic acid concentrations and resistant to low pH values) to compete with *Methanosaeta* (always active but inhibited at low pH values). In our experiments, acetic acid accumulations of 2.1 gCOD L^−1^ accompanied by a pH drop to below 6.75 were sufficient to achieve a relative *Methanosarcina* abundance of about 30% of total methanogens (Experiments 1 and 2). The shift of the methanogenic community towards higher shares of *Methanosarcina* depends on the intensity of VFA accumulation after the discontinuous feeding. Low acetic acid accumulations of 0.5 gCOD L^−1^, for example, led to an increase of only 7 percentage points in relative abundance of *Methanosarcina* on total methanogens after 5 HRTs (Experiment 3). The use of T-RFLP fingerprinting based on DNA and cDNA of *mcrA* genes and transcripts as a monitoring tool for shifts towards higher shares of *Methanosarcina* was demonstrated. Finally, our model simulations with maize silage as a more complex and practically relevant substrate showed that our microbial resources management scheme is likely transferable to the complete AD process. This scheme could be used to strengthen microbial communities for withstanding organic overloads that might, for example, occur in demand-driven biogas production schemes.

## Methods

### Laboratory-scale CSTR experiments and process analytics

Three laboratory-scale CSTR experiments were conducted at 37 °C with various HRTs, OLRs and feeding regimes (see Fig. 1). The working volume was 6 L for Experiments 1 and 2, and 8 L for Experiment 3. Continuous stirring at 50 rpm with anchor-type impellers was applied in all experiments. In all experiments, a synthetic, liquid substrate was used comprising a mixture of VFAs as the only carbon sources in a mineral medium containing all necessary trace elements, macronutrients, and vitamins (see Additional file 1, Table S1). The VFA composition in this medium was 45% acetic acid, 10% propionic acid, and 45% butyric acid based on COD, which corresponds to 65%, 8% and 26% on mol-base, and 57%, 9% and 34% on mass-base for acetic, propionic and butyric acid, respectively. 1 gCOD L^−1^ of this medium corresponds to 0.74 g L^−1^ and 14 mM.

In each experiment, at least two reactors were run in parallel with two different feeding regimes: a continuously fed (*R*_conti_) and a discontinuously fed reactor (*R*_disco_). Biological replicate reactors were designated as R_conti,B_ or R_disco,B_. Discontinuous feeding means that a certain amount (75–100% of daily feed, see Fig. 1) was pumped into the reactor within 20 min. In the “start-up phase”, the reactors were slowly accustomed to the selected feeding regime. In the following “training phase”, the selected feeding regime was applied for at least 3 HRTs. In the following “disturbance phase”, the *R*_disco_ and *R*_conti_ were exposed to a pulse disturbance with the same VFA mixture used as substrate (see Fig. 1) but higher concentrations than the usual feeding to simulate organic overloading.

All CSTRs were inoculated from a lab scale continuously fed digester operated with the same synthetic substrate. At the start of the experiment, the content of all CSTRs was mixed to guarantee a homogenous inoculum. VS values of the inoculum were on average 0.87, 0.41 and 0.46 g L^−1^ for Experiments 1, 2 and 3, respectively. Total VFA concentrations in the inoculum were below 0.5 gCOD L^−1^ in all experiments (see Additional file 2, abiotic parameters, *t* = 0 days for details).

Biogas composition (CH_4_, CO_2_, O_2_, H_2_, and H_2_S) was analyzed with an AWIFLEX gas analyzer (AWITE Bioenergie, Germany). Rate of biogas production was determined by drum-type gas meters TG05 (Ritter, Germany) and normalized to standard temperature (237.15 K) and standard pressure (101,325 Pa). VFA concentrations as well as pH values were analyzed as reported previously [1].

### Microbial community analyses

#### DNA and RNA extraction

The reactors were sampled before feeding if not indicated otherwise. Samples (1.5 mL) from the reactors were centrifuged immediately after sampling at − 7 °C and 15,000×*g* for 2 min and the supernatant was removed. The samples did not freeze within the 2 min despite the low temperature, which was chosen to minimize the risk of mRNA degradation. Pellets for DNA extraction were stored at − 20 °C. Pellets for RNA extraction were stored at − 80 °C.

DNA was extracted with the NucleoSpin^®^ Soil Kit (MACHEREY–NAGEL GmbH & Co. KG, Germany) following the manufacturer’s instructions (buffer SL2, no enhancer solution). The quantity and quality of extracted DNA were determined by NanoDrop^®^ ND 1000 spectrophotometer (Thermo Fisher Scientific, USA). The DNA was stored at − 20 °C.

RNA was extracted with the ZR Soil/Fecal RNA MicroPrep™ Kit (Zymo Research, USA) following the manufacturer’s instructions. DNA was removed from the isolated RNA using the DNA-*free*™ DNA Removal Kit (Invitrogen™, Thermo Fisher Scientific, USA). The quantity and quality of extracted RNA were determined by NanoDrop^®^ ND 1000 spectrophotometer (Thermo Fisher Scientific, USA). The RevertAid H Minus First Strand cDNA Synthesis Kit (Thermo Scientific™, Thermo Fisher Scientific, USA) with random hexamer primers was used to synthesize cDNA from the total RNA following the manufacturer’s instructions. T-RFLP analysis of cDNA was performed as described below (“Composition and dynamics of methanogenic communities” section).

#### Composition and dynamics of methanogenic communities

For a detailed analysis of abundances and activities of methanogenic archaea, T-RFLP analysis of *mcrA* gene DNA and cDNA amplicons was used as described previously [41]. The primers mlas (GGTGGTGTMGGDTTCACMCARTA) and mcrA-rev (CGTTCATBGCGTAGTTVGGRTAGT) were used [42]. *Bst*NI (New England Biolabs) was used as restriction enzyme. A previously published database was used to assign families to the detected T-RFs [43]. Methanogens are supposed to contain only one copy of *mcrA* per genome [42]. Methanobacteriales and Methanococcales contain one copy of the *mrtA* gene, which is also amplified by the primers mlas and mcrA-rev [42]. To convert T-RF abundances into genome abundances, the T-RFs of these two orders were corrected by the factor 2.

#### Composition of bacterial communities

Bacterial community compositions were analyzed by amplicon sequencing of 16S rRNA genes. PCR amplification and sequencing with the MiSeq platform (V3, 2 × 300 bp, Illumina) were performed by LGC Genomics GmbH (Berlin, Germany). The primers 341f (CCTACGGGNGGCWGCAG) and 785r (GACTACHVGGGTATCTAAKCC) targeting the V3–V4 regions were used [44]. LGC Genomics processed the sequencing data by de-multiplexing and removing barcodes (max. 1 mismatch), adapter as well as primer sequences (max. 3 mismatches). Raw de-multiplexed sequence data were deposited at EMBL European Nucleotide Archive (ENA) under accession number PRJEB27523 (http://www.ebi.ac.uk/ena/data/view/PRJEB27523). The software BBMerge 34.48 (http://bbmap.sourceforge.net/) was used to merge forward and reverse reads using a minimum overlap of 12 bp. The QIIME 1.9.1 Virtual Box [45] was used for further data analysis: quality filtering was performed (threshold < 20, truncation after three consecutive low quality bases, no ambiguous base calls), and chimeras were removed. Reads were clustered into OTUs using the usearch tool and a minimum cluster size of 4 to remove spurious reads [46]. Taxonomic assignment was performed using MiDAS taxonomy 2.1 [47] and the RDP classifier 2.2 (confidence threshold 0.8) [48]. The OTU table was rarefied to 41,305, 18,288, and 57,507 sequences per sample for Experiments 1, 2, and 3, respectively (see Additional file 1, Figure S10). Archaeal 16S rRNA genes are only partially amplified by the applied primers and the resulting archaeal community composition may, therefore, be biased [44]. Thus, the methanogenic community composition was not analyzed based on the amplicon sequencing data.

The relative taxon-specific 16S rRNA gene abundances were converted to relative taxon-specific genome abundances by the average strain-specific 16S rRNA operon copy number per genome [49]. The 16S rRNA gene operon numbers per genome for each OTU at genus level were taken from the rrnDB database version 5.2 using the taxonomy search function (https://rrndb.umms.med.umich.edu/, accessed on 05.09.2017, Name type: NCBI—all names). If the copy number was not available at the genus level, the next higher taxonomic level was chosen.

#### Inference of biological network associations using CoNet

The weights of statistically significant (Pearson correlation, threshold = 0.05) mutual exclusions were calculated using the CoNet App (v 1.1.1 beta) inside Cytoscape (v 3.6.0). The command ‘column normalization’ as standardization method and otherwise default settings were used. All 23 bacterial community compositions from all experiments were included in the analysis. Statistically significant mutual exclusions of OTUs with *Syntrophomonas* and *Syntrophobacter* were interpreted as potential butyric and propionic acid oxidizers, respectively (see Additional file 1, Section 2.2.5).

#### Diversity and evenness indices

Diversity indices ^*q*^*D* and evenness indices ^*q*^*E* were calculated for the bacterial communities at the end of the training phase of Experiment 1 (*t* = 64 days) and Experiment 2 (*t* = 55 days) according to [28]. The parameter *q* determines the weight on rare versus abundant OTUs in the calculation of the indices. For *q *= 0, ^*0*^*D* equals the number of OTUs in a sample regardless of their relative abundance (richness). For *q *= 1, OTUs are weighted by their relative abundance (equals the exponentiated Shannon index). For *q *= 2, abundant OTUs are weighted more than rare OTUs (equals the reciprocal of the Simpson’s index). The evenness ^*q*^*E* for each sample is given by ^*q*^*D*/^*0*^*D*.

#### Absolute quantification of microbial COD and biomass

*Microbial biomass* No particulate organic matter was added to the influent of the reactors. Therefore, the volatile solids (VS) concentration in the reactor effluent was assumed to equal the microbial biomass dry weight concentration. Total solids (TS) and VS were analyzed as reported previously [1] except that due to the low TS and VS contents in the reactors, crucibles were not filled directly with sample, but with the pellet from 100 mL samples after centrifugation (10,000×*g*, 10 min, 10 °C).

*Microbial COD* The COD of the microbial biomass was determined by centrifuging 500 μL reactor sample (15,000×*g*, 2 min, 4 °C) removing the supernatant, washing the pellet with phosphate buffered saline (PBS) solution, centrifuging the pellet, removing the supernatant, resuspending the pellet in 2 mL deionized water and adding it into the COD cuvette test LCK 714 (Hach Lange GmbH, Germany).

#### [2-^13^C]-acetate labeling experiment

[2-^13^C]-acetate labeling batch experiments were conducted in biological triplicates for each reactor from Experiment 1. Each serum bottle had a total volume of 122 mL, a working volume of 20 mL and was filled with 18.74 mL reactor effluent and 1.26 mL of 1 M [2-^13^C]-acetate (Sigma Aldrich, USA) in an anaerobic chamber (97% N_2_, 3% H_2_). The bottles were incubated at 37 °C for 20 h. Gas composition was analyzed using the gas chromatograph Clarus 580 (PerkinElmer, Germany) and the ratio of ^12^CO:^13^CO_2_ as well as ^12^CH_4_:^13^CH_4_ was determined using the gas chromatography-mass spectrometer (GC–MS) Clarus 600 (PerkinElmer, Germany) as described previously [50].

### ADM1 simulation

The original ADM1 model structure [51] was changed in several aspects. The ordinary differential equations for inorganic carbon and inorganic nitrogen were amended by additional balancing terms to close mass balances in all processes. The overpressure in the headspace was used to calculate the biogas production rate. Standard parameters [52] were used if not indicated otherwise. The cation (*S*_cat,in_) and anion influent concentration (*S*_an,in_) did not need to be fitted but could be determined based on the known composition of the synthetic medium used in the experiments to 78.3 and 19.8 mM for *S*_cat,in_ and *S*_an,in_, respectively.

The single population of acetoclastic methanogens (X_ac_) was split into two separate populations *Methanosarcina* (X_ac,1_) and *Methanosaeta* (X_ac,2_). The differential equation for acetic acid concentration was adapted accordingly. The original ADM1 inhibition functions [51] were used for both X_ac,1_ and X_ac,2_ with default parameter values [52] except for upper (pH_u_ac) and lower limits (pH_l_ac) for pH inhibition (see Table 1). The complete model structure can be found as Petersen Matrix in Additional file 2.

The kinetic growth parameters *k*, *Y* and *K* for acetic, propionic and butyric acid degraders were manually optimized to fit the experimental total microbial biomass concentration, the VFA concentration and the ratio of *Methanosarcina* and *Methanosaeta* at the end of the training phase as well as pH changes after the disturbance of both *R*_disco_ and *R*_conti_ in Experiment 1 (see Table 1).

The parameter set established for the lab-scale reactors was also applied to simulate a maize silage-fed digester. Only the HRT (20 days) and the substrate input composition for maize silage [53] were changed. Disintegration, hydrolysis and acidogenesis were simulated with standard parameters [52]. Continuous feeding was simulated until steady state was reached and then switched to discontinuous feeding (every second day). Initial conditions, input concentrations, and parameters of all simulations can be found in Additional file 2.

## Additional files

**Additional file 1.** Details on Methods and Results. Composition of mineral medium, details on experimental design, detailed process performance data of all experiments, detailed results of microbial community analysis, detailed ADM1 simulation results for all experiments.

**Additional file 2.** Further details on Methods and Results. ADM1 parameter values of all simulations, Petersen Matrix, detailed process performance data of all experiments, detailed results of microbial community analysis.

**Additional file 3.** Bacterial community composition. Zoomable pie chart using the visualization tool Krona.
