# Supplementary material for: Intermittent fasting for microbes: how discontinuous feeding increases functional stability in anaerobic digestion
Source: Biotechnol Biofuels. 2018 Oct 6;11:274. doi: 10.1186/s13068-018-1279-5 (PMC6173896; doi:10.1186/s13068-018-1279-5)
Supplement: Supplementary file 1 — Additional file 1. Details on Methods and Results. Composition of mineral medium, details on experimental design, detailed process performance data of all experiments, detailed results of microbial community analysis, detailed ADM1 simulation results for all experiments. [file 13068_2018_1279_MOESM1_ESM.docx]

Additional File 1

*for*

**Intermittent fasting for microbes: How discontinuous feeding leads to higher functional stability in anaerobic digestion**

Fabian Bonk^1*^, Denny Popp^1^, Sören Weinrich^2^, Heike Sträuber^1^, Sabine Kleinsteuber^1^, Hauke Harms^1^, Florian Centler^[[1]](#footnote-1)^

^1^ Helmholtz Centre for Environmental Research – UFZ, Department of Environmental Microbiology, Permoserstr. 15, 04318 Leipzig, Germany
^2^ DBFZ – Deutsches Biomasseforschungszentrum gemeinnützige GmbH, Department Biochemical Conversion, Torgauer Str. 116, 04347 Leipzig, Germany

# List of contents

List of Figures 1

List of Tables 3

A.1 Additional material and methods 4

A.1.1 Composition of the mineral medium 4

A.1.2 Details on experimental design 6

A.2 Supplementary results 7

A.2.1 Process performance data 7

A.2.1.1 Experiment 1 8

A.2.1.2 Experiment 2 10

A.2.1.3 Experiment 3 14

A.2.1.4 Deriving kinetic data from the daily VFA profile of R_disco_ for Experiment 1 17

A.2.1.5 Comparison of substrate conversion efficiency and pH in the three experiments 25

A.2.2 Bacterial community 16S rRNA sequencing 26

A.2.2.1 Rarefaction curves 26

A.2.2.2 Overview on community composition 27

A.2.2.3 Bacterial diversity and evenness 35

A.2.2.4 Abundance of syntrophic acetic acid oxidizing bacteria (SAOB) 35

A.2.2.5 Inference of biological network associations using CoNet 37

A.2.2.6 Specialists for continuous and discontinuous feeding, generalists and most abundant members of the bacterial community 40

A.2.3 Methanogenic community structure 42

A.2.3.1 T-RFLP DNA 42

A.2.3.2 T-RFLP cDNA 44

A.2.4 Ratio of bacteria to archaea 47

A.2.5 [2-^13^C]-acetate labelling experiment 48

A.2.6 ADM1 Model 49

A.2.6.1 Growth kinetic parameters 49

A.2.6.2 Detailed simulation results of Experiment 1 52

A.2.6.3 Simulation results of Experiment 2 and Experiment 3 56

A.2.6.4 Discontinuously fed maize silage reactor 64

**References** 66

# List of Figures

Figure A.1 Abiotic results of Experiment 1: (a) pH, (b) methane production rate, (c) total VFA concentration and (d) microbial biomass. Error bars represent 1 standard error of the mean (SEM). 9

Figure A.2 Abiotic results of Experiment 2: (a) pH, (b) methane production rate, (c) total VFA concentration, (d) microbial biomass as well as (e) pH and (f) VFA concentrations in disturbance phase. Error bars represent 1 SEM. Disturbance at 55d for R_disco_ and 68d for R_conti_. 12

Figure A.3 Abiotic results of Experiment 3: (a) pH, (b) methane production rate, (c) total VFA concentration, (d) microbial biomass and (e) VFA concentration after disturbance. Error bars represent 1 SEM. 16

Figure A.4 VFA concentrations and pH during a daily feeding interval of Experiment 1. Error bars represent 1 SEM. 17

Figure A.5 Rate of butyric acid consumption after discontinuous feeding (a) as a function of butyric acid concentration in the reactor and (b) as a function of time after discontinuous feeding.. 18

Figure A.6 Rate of propionic acid consumption after discontinuous feeding (a) as a function of propionic acid concentration in the reactor and (b) as a function of time after discontinuous feeding ,(c) in comparison with butyric acid consumption rate and (d) as a function of pH. (e) Gibbs energy change (ΔG1,T) of propionate and butyrate oxidation corrected for the reactor temperature (T) of 37°C and substrate and product concentrations and pH measured 10h after feeding as function of hydrogen partial pressure. Calculations were based on [2]. Concentrations were used instead of activities.... 22

Figure A.7 Rate of acetic acid consumption after discontinuous feeding (a) as a function of acetic acid concentration in the reactor and (b) as a function of time after discontinuous feeding.. 23

Figure A.8 Predicted rate of hydrogen production from propionic and butyric acid oxidation as a function of time after discontinuous feeding.. 24

Figure A.9 Equal process performance (a) but different pH (b) for the different reactors at the end of the training phase of each experiment (Exp.). Substrate conversion was based on VFA concentrations in influent and effluent. R_disco_ stands for discontinuously fed and R_conti_ for continuously fed reactor. 25

Figure A.10 Rarefaction curves for (a) Experiment 1, (b) Experiment 2 and (c) Experiment 3.. 27

Figure A.11 Bacterial OTUs at the end of the training phase. Edwards-Venn diagram generated using jvenn [3] for the bacterial OTUs at the end of the training phase of Experiment 1, Experiment 2 and Experiment 3. Asterisks mark technical replicates. 30

Figure A.12 Bacterial OTUs at the end of the training phase. Taxonomic profiles for Experiment 1 (a), Experiment 2 (b), Experiment 3 (c). Other OTUs are the sum of OTUs with relative abundances smaller than 1%. Asterisks mark technical replicates. 33

Figure A.13 Nonmetric Multidimensional Scaling (NMDS) plot using R (library “vegan”, function metaMDS using Bray–Curtis dissimilarities) of relative bacterial community composition at the end of the training phase. 34

Figure A.14 Diversity and Evenness of the bacterial community of Rdisco and Rconti at the end of the training phase (only reactors with disturbance experiment). 35

Figure A.15 T-RFLP profiles of the methanogenic community on DNA level. T-RFs that could not be assigned are labelled as "Other". ”Ambiguous on family level” stands for *Methanocorpusculaceae, Methanomicrobiaceae*, *Methanoregulaceae*, and *Methanospirillaceae* which cannot be distinguished by our T-RFLP analysis. 43

Figure A.16 T-RFLP analysis of the methanogenic community on cDNA level. T-RFs that could not be assigned are labelled as "Other". ”Ambiguous on family level” stands for *Methanocorpusculaceae, Methanomicrobiaceae*, *Methanoregulaceae*, and *Methanospirillaceae* which cannot be distinguished by our T-RFLP analysis. 45

Figure A.17 T-RFLP analysis of the methanogenic community on cDNA level before and after discontinuous feeding. Comparison of all experiments. T-RFs that could not be assigned are labelled as "Other". ”Ambiguous on family level” stands for *Methanocorpusculaceae, Methanomicrobiaceae*, *Methanoregulaceae*, and *Methanospirillaceae* which cannot be distinguished by our T-RFLP analysis. 46

Figure A.18 Relative abundance of bacteria over total prokaryotes based on 16S rRNA sequencing results. Genome abundances are derived by correcting for the average bacterial 16S rRNA operon copy number per genome for each sample. 47

Figure A.19 ADM1 simulation results for Experiment 1. Disturbance at day 64 d. (a) total VFA concentration and (b) acetic acid concentration. 52

Figure A.20 ADM1 simulation results for total microbial biomass concentrations for Experiment 1. Disturbance at day 64 d. 53

Figure A.21 ADM1 simulation for Experiment 1 with increased maximum specific substrate uptake rates (51 gCOD_ac_ L^-1^ d^-1^) and decreased half-saturation constants (0.001 gCOD L^-1^) to compare the maximum pH for both feeding regimes in Experiment 1 independent of residual VFA anions. (a) pH values, (b) VFA concentrations and (c) dissolved inorganic carbon concentrations. 55

Figure A.22 ADM1 simulations for Experiment 2 (a) pH, (b) total VFA concentration, (c) total microbial biomass concentration and (d) acetoclastic methanogens. 59

Figure A.23 ADM1 simulations for Experiment 3 (a) pH, (b) total VFA concentration, (c) total microbial biomass concentration and (d) acetoclastic methanogens. 63

Figure A.24 ADM1 simulation of a digester fermenting maize silage using the same growth kinetic parameter values of Experiment 1 (a) continuous feeding and (b) discontinuous feeding. 65

# List of Tables

Table A.1 Composition of mineral medium 4

Table A.2 Details on experimental design 6

Table A.3 Abundance of known SAO oxidizing bacteria or related taxa in our experiments 36

Table A.4 Network analysis for *Syntrophomonas*: Pearson correlation (threshold=0.05) for all samples (n=23) 38

Table A.5 Network analysis for *Syntrophobacter*: Pearson correlation (threshold=0.05) for all samples (n=23) 39

Table A.6 Network analysis for *Syntrophobacter* and *Syntrophomonas*: Pearson correlation (treshold=0.05) for all samples (n=23) 39

Table A.7 Specialists for continuous and discontinuous feeding, generalists and most abundant members of the bacterial community 41

Table A.8 Ratio of 13C-labelled acetate to 13C-labelled CO_2_ in batch experiment for Experiment 1 48

# A.1 Additional material and methods

## A.1.1 Composition of the mineral medium

The synthetic medium consisted of two components (component A and component B, equal volume) which were kept separated until feeding to avoid precipitation and microbial growth during storage (see Table A.1).

Table A.1 Composition of mineral medium

| **Compound** | **Concentration in partitioned media (mg L^-1^)** | **Final concentration in CSTR influent (mg L^-1^)** |
| --- | --- | --- |
| **Component A** |  |  |
| Acetic acid | 31,420 for Experiment 1 and 2 10,470 for Experiment 3 | 15,710 for Experiment 1 and 2 72,000 for Experiment 3 |
| Propionic acid | 4,920 for Experiment 1 and 2 1,640 for Experiment 3 | 2,460 for Experiment 1 and 2 820 for Experiment 3 |
| Butyric acid | 18,440 for Experiment 1 and 2 6,147 for Experiment 3 | 9,220 for Experiment 1 and 2 3,073 for Experiment 3 |
| KCl | 600 | 300 |
| MgCl_2_×6H_2_O | 600 | 300 |
| CaCl_2_×2H_2_O | 200 | 100 |
| Na_2_S×9H_2_O | 500 | 250 |
| FeCl_2_×4H_2_O | 42.36 | 21.18 |
| CuCl_2_×2H_2_O | 0.86 | 0.43 |
| CoCl_2_×6H_2_O | 1.94 | 0.97 |
| MnCl_2_×4H_2_O | 1.64 | 0.82 |
| Na_2_MoO_4_×2H_2_O | 0.86 | 0.43 |
| NiCl_2_×6H_2_O | 3.28 | 1.64 |
| Na_2_WO_4_×2H_2_O | 0.36 | 0.18 |
| Na_2_SeO_3_×5H_2_O | 0.8 | 0.40 |
| ZnCl_2_ | 4.72 | 2.36 |
| H_3_BO_3_ | 4.96 | 2.48 |
| Biotin | 0.04 | 0.02 |
| Folic acid | 0.04 | 0.02 |
| Pyridoxine | 0.2 | 0.1 |
| Thiamine | 0.1 | 0.05 |
| Riboflavin | 0.1 | 0.05 |
| Nicotinic acid | 0.1 | 0.05 |
| Ca-pantothenate | 0.1 | 0.05 |
| B12 | 0.1 | 0.05 |
| p-aminobenzoate | 0.1 | 0.05 |
| Lipoic acid | 0.1 | 0.05 |
|  |  |  |
| **Component B** |  |  |
| KH_2_PO_4_ | 1000 | 500 |
| NH_4_HCO_3_ | 8190 | 4095 |
| NaOH | 5600 | 2800 |

## A.1.2 Details on experimental design

The detailed experimental set-up for the reactor experiments can be found in Table A.2.

Table A.2 Details on experimental design

|  | **day** | **R_conti,A_** | **R_conti,B_** | **R_disco,A_** | **R_disco,B_** |
| --- | --- | --- | --- | --- | --- |
| **Experiment 1** |  |  |  |  |  |
| Phase 1: start-up | 0-29 | See method section, | See method section, | See method section, but working volume (V_w_) change between 6 L and 7 L | N/A |
| Phase 2: training | 29-64 | See method section | See method section | See method section (V_w_) change between 5 and 6 L) | N/A |
| Phase 3: disturbance | 64-80 | control, no disturbance | See method section, disturbance on day 64 | See method section, disturbance on day 64 | N/A |
| **Experiment 2** |  |  |  |  |  |
| Phase 1: start-up | 0-11 | Continuous feeding. Increase of VFA concentration in substrate from 12.4 to 37.2 g COD L^‑1^ | N/A | Discontinuous feeding (100% daily feed at once) Increase of VFA concentration in substrate from 12.4 to 37.2 g COD L^‑1^ | N/A |
| Phase 2: training | 11-55 (R_disco_) and 11-68 (R_conti_) | see Method section | N/A | see Method section | N/A |
| Phase 3: disturbance | 55-61 (R_disco_) and 68-74 (R_conti_) | see Method section, disturbance at day 68 | N/A | see Method section, disturbance at day 55 | N/A |
| **Experiment 3** |  |  |  |  |  |
| Phase 1: start-up | 0-30 | Continuous feeding | N/A | Increasing amount discontinuous (daily pulse) feed, decreasing amount continuous feed | Increasing amount discontinuous (daily pulse) feed, decreasing amount continuous feed |
| Phase 2: training | 31-58 | Continuous feeding | N/A | See Method section | See Method section |
| Phase 3: disturbance | 59-71 | Day 59: no propionic and butyric acid fed; day 63: no acetic and propionic acid fed; day 66 no acetic and butyric acid fed; day70: VFA shock as described in Figure 1 | N/A | No disturbances | Day 59: no propionic and butyric acid fed; day 63: no acetic and propionic acid fed; day 66 no acetic and butyric acid fed; day70: VFA shock as described in Figure 1 |

## A.2 Supplementary results

## A.2.1 Process performance data

Methane production rates, pH, microbial biomass concentrations and total VFA concentrations of the reactor experiments can be found in Figure A.1 (Experiment 1), Figure A.2 (Experiment 2), Figure A.3 (Experiment 3). Error bars represent one standard error of the mean (SEM).

### A.2.1.1 Experiment 1

Figure A.1 Abiotic results of Experiment 1: (a) pH, (b) methane production rate, (c) total VFA concentration and (d) microbial biomass. Error bars represent 1 standard error of the mean (SEM).

### A.2.1.2 Experiment 2

Figure A.2 Abiotic results of Experiment 2: (a) pH, (b) methane production rate, (c) total VFA concentration, (d) microbial biomass as well as (e) pH and (f) VFA concentrations in disturbance phase. Error bars represent 1 SEM. Disturbance at 55d for R_disco_ and 68d for R_conti_.

The differences in methane production rate were most likely the result of technical issues since in both reactors, the identical amount of substrate was converted. Due to technical limitations, the biogas composition in both reactors could only be measured twice per day. Since the biogas composition and biogas production rate changed over the course of a day drastically in R_disco_, the resulting methane production rate was inaccurate.

### A.2.1.3 Experiment 3

Figure A.3 Abiotic results of Experiment 3: (a) pH, (b) methane production rate, (c) total VFA concentration, (d) microbial biomass and (e) VFA concentration after disturbance. Error bars represent 1 SEM.

### A.2.1.4 Deriving kinetic data from the daily VFA profile of R_disco_ for Experiment 1

In order to derive information on the substrate conversion rate and microbial activity over a day in R_disco_, samples for RNA extraction as well as for pH and VFA measurements were taken at least every 2 hours over the course of 24 hours (see Figure A.4). From this data, butyric acid (Figure A.5), propionic acid (Figure A.6), acetic acid (Figure A.7), and hydrogen (Figure A.8) consumption rates were estimated according to Equation (A.1a), Equation (A.1b), Equation (A.1c) and Equation (A.1d), respectively.

$r_{but}\left( t \right)=\frac{S_{but}\left( t-\Delta t \right)-S_{but}\left( t \right)}{\Delta t}$ (Equation A.1a)

$r_{pro}\left( t \right)=\frac{S_{pro}\left( t-\Delta t \right)-S_{pro}\left( t \right)}{\Delta t}$ (Equation A.1b)

$r_{ac}\left( t \right)=\frac{S_{ac}\left( t-\Delta t \right)-S_{ac}\left( t \right)}{\Delta t}+1.4\cdot r_{but}\left( t \right)+0.8\cdot r_{pro}\left( t \right)$ (Equation A.1c)

$r_{h2}\left( t \right)=0.046\cdot r_{but}\left( t \right)+0.082\cdot r_{pro}\left( t \right)$ (Equation A.1d)

with *r_i_*(*t*) being the conversion rate of substrate *i* [mg L^-1^ d^-1^], *S_i_* the concentration of substrate *i* [mg L^-1^], *t* a sampling point, *Δt* the time difference to the previous sampling point [d] and the numbers are stoichiometric parameters [g g^-1^] for the hydrogen and acetic acid production from propionic and butyric acid oxidation neglecting biomass build up.

Figure A.4 VFA concentrations and pH during a daily feeding interval of Experiment 1. Error bars represent 1 SEM.

#### Butyric acid

The maximal butyric acid consumption rate equaled 203.4 mg L^-1^ h^-1^ corresponding to 8.89 gCOD L^-1^d^-1^ (Figure A.5). Assuming a maximal electron transfer rate of 51 gCOD gCOD^-1^ d^-1^ [1], the measured maximum butyric acid consumption rate corresponded to at least 0.174 gCOD L^-1^ of butyric acid degrading microbial biomass (X_bu_).

Figure A.5 Rate of butyric acid consumption after discontinuous feeding (a) as a function of butyric acid concentration in the reactor and (b) as a function of time after discontinuous feeding.

#### Propionic acid

The maximal propionic acid consumption rate equaled 33.04 mg L^-1^ h^-1^ corresponding to 1.2 gCOD L^-1^d^-1^ (Figure A.6). Assuming a maximal electron transfer rate of 51 gCOD gCOD^-1^ d^-1^ [1], the measured maximum propionic acid consumption rate corresponded to at least 0.024 gCOD L^-1^ of propionic acid degrading microbial biomass (X_pro_).

Propionic acid oxidation rate appeared to decrease when butyric acid degradation rate increased and vice versa (Figure A.6c). This might be due to the fact that both reactions depend on a low hydrogen partial pressure and thus on hydrogenotrophic methanogens in proximity. Due to the high amount of hydrogen produced from butyric acid oxidation as a result of the much higher microbial abundance and the lower Gibbs energy, propionic acid oxidation might become unfeasible. Propionate oxidation becomes unfeasible at a hydrogen partial pressure of about 100 ppm for the environmental conditions measured in R_disco_ 10h after the feeding, when the propionic acid consumption rate is close to 0 (see Figure A.6e). For the same sampling point, butyrate oxidation is feasible up to a hydrogen partial pressure of about 7000 ppm. We measured hydrogen partial pressures of 0 that day, but the error of the detector is about 60 ppm and hydrogen partial pressures could be measured only twice a day. Therefore, a temporarily elevated hydrogen partial pressure inhibitory for propionate oxidation might have occurred unnoticed. An alternative hypothesis could be that propionic acid oxidizers are more sensitive to pH than butyric acid oxidizers but since an increase in rate was only visible after the pH raised over pH 7.5 this seems unlikely. Furthermore, the two rate peaks might be caused by two different propionic acid oxidizing populations which became active in certain niches.

.

**(e)**

Figure A.6 Rate of propionic acid consumption after discontinuous feeding (a) as a function of propionic acid concentration in the reactor and (b)as a function of time after discontinuous feeding ,(c) in comparison with butyric acid consumption rate and (d) as a function of pH. (e) Gibbs energy change (ΔG^1,T^) of propionate and butyrate oxidation corrected for the reactor temperature (T) of 37°C and substrate and product concentrations and pH measured 10h after feeding as function of hydrogen partial pressure. Calculations were based on [2]. Concentrations were used instead of activities.

#### Acetic acid

The maximal acetic acid consumption rate equaled 323.22 mg L^-1^ h^-1^ corresponding to 8.3 gCOD L^-1^d^-1^ (Figure A.7). Assuming a maximal electron transfer rate of 51 gCOD gCOD^-1^ d^-1^ [1], the measured maximum acetic acid consumption rate corresponded to at least 0.163 gCOD L^-1^ of acetic acid degrading microbial biomass (X_ac_). Assuming X_ac_ to 0.96 gCOD_Xac_ L^-1^ (microbial biomass concentration based on Y_ac_=0.033 gCOD_Xac_ gCOD_ac_ and full substrate conversion, see Section A.2.6.1), k_m_ needs to be at least 8.7 gCOD_ac_ gCOD_Xac_d^-1^.

Figure A.7 Rate of acetic acid consumption after discontinuous feeding (a) as a function of acetic acid concentration in the reactor and (b) as a function of time after discontinuous feeding.

#### Hydrogen

Assuming that hydrogen was consumed as fast as it was produced (since no accumulation was measured), the maximal hydrogen consumption rate equaled 88.4 mgCOD L^-1^ h^-1^ corresponding to 2.12 gCOD L^-1^d^-1^ (Figure A.8). Assuming a maximal electron transfer rate of 51 gCOD gCOD^-1^ d^-1^ [1], the inferred maximum hydrogen consumption rate corresponded to at least 0.0416 gCOD L^-1^ of hydrogen degrading microbial biomass (X_h2_).

Figure A.8 Predicted rate of hydrogen production from propionic and butyric acid oxidation as a function of time after discontinuous feeding.

### A.2.1.5 Comparison of substrate conversion efficiency and pH in the three experiments

Figure A.9 shows the substrate conversion efficiency and pH for all experiments and reactors at the end of the training phase. All reactors converted the substrate to almost 100%. Nevertheless, the discontinuously fed reactors showed higher pH values in all experiments. The pH values were determined with the same pH-meter for all reactors during an experiment.

Figure A.9 Equal process performance in terms of substrate conversion (a) but different pH (b) for the different reactors at the end of the training phase of each experiment (Exp.). Substrate conversion was based on VFA concentrations in influent and effluent. Rdisco stands for discontinuously fed and Rconti for continuously fed reactor.

## A.2.2 Bacterial community 16S rRNA sequencing

### A.2.2.1 Rarefaction curves

The rarefaction curves of the 16S rRNA amplicon sequencing are presented in Figure A.10.

(a)

(b)

(c)

Figure A.10 Rarefaction curves for (a) Experiment 1, (b) Experiment 2 and (c) Experiment 3.

### A.2.2.2 Overview on community composition

The bacterial community compositions are presented as Edwards-Venn-Diagrams for the end of the training phase (Figure A.11), as bar charts for all samples (Figure A.12) and as Nonmetric Multidimensional Scaling (NMDS) plot for the end of the training phase (Figure A.13). In many samples, *Syntrophobacter* and *Syntrophomonas* were highly abundant which is not surprising given their known role as propionic and butyric acid oxidizer, respectively. Furthermore, members of the *Synergistaceae*, in particular *Thermovirga* and an uncultured one, were found to be highly abundant in several samples. Furthermore, the phylum Cloacimonetes was found to be highly abundant in several samples.

As generalists with genome abundances >0.7% in all samples, only Cloacimonetes, *Syntrophomonas, Syntrophobacter*, an uncultured member of *Spirochaetaceae* and *Thermovirga* were found.

Number of OTUs per sample

Number of OTUs: specific (1) or shared by 2,3,… samples

Experiment 1

Number of OTUs per sample

Number of OTUs: specific (1) or shared by 2,3,… samples

Experiment 2

Number of OTUs: specific (1) or shared by 2,3,… samples

Number of OTUs per sample

Number of OTUs: specific (1) or shared by 2,3,… samples

Number of OTUs per sample

Experiment 3

Figure A.11 Bacterial OTUs at the end of the training phase. Edwards-Venn diagram generated using jvenn [3] for the bacterial OTUs at the end of the training phase of Experiment 1, Experiment 2 and Experiment 3. Asterisks mark technical replicates.

Figure A.12 Bacterial OTUs at the end of the training phase. Taxonomic profiles for Experiment 1 (a), Experiment 2 (b), Experiment 3 (c). Other OTUs are the sum of OTUs with relative abundances smaller than 1%. Asterisks mark technical replicates.


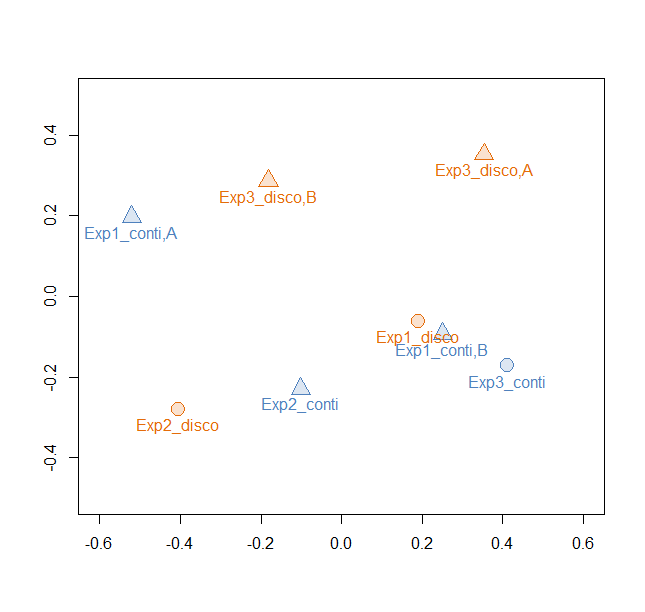


Stress=0.0678

Figure A.13 Nonmetric Multidimensional Scaling (NMDS) plot using R (library “vegan”, function metaMDS using Bray–Curtis dissimilarities) of relative bacterial community composition at the end of the training phase.

### A.2.2.3 Bacterial diversity and evenness

In Experiment 1 and 2, a process failure of R_conti_ was observed after a deliberate disturbance while R_disco_ recovered. A higher bacterial diversity and evenness might be a reason for this. In Experiment 2, evenness and diversity parameter values are higher for R_disco_. But for Experiment 1, the parameter values are mostly similar (see Figure A.14 and Additional File 2, sheet “microbial diversity” for more details). Therefore, the higher functional resilience in our experiments does not seem to depend on diversity and/or evenness of the bacterial community.

Figure A.14 Diversity and Evenness of the bacterial community of R_disco_ and R_conti_ at the end of the training phase (only reactors with disturbance experiment).

### A.2.2.4 Abundance of syntrophic acetic acid oxidizing bacteria (SAOB)

There are five described species of SAOB: *Pseudothermotoga lettingae*, *Thermacetogenium phaeum*, *Clostridium ultunense*, *Syntrophaceticus schinkii* and *Tepidanaerobacter acetatoxydans* (Müller et al., 2016)). In addition, OTU_M_H_C (related to *Alkaliphilus halophilus*) was found to be abundant in chemostats fed only with acetic acid and also hypothesized to be a SAOB [4]. In our reactors, these SAOB or closely related taxa only played a minor role (see Table A.3).

Table A.3 Abundance of known SAOB or related taxa in our experiments

| Known SAOB | Related OTUs in our reactors (relative genome abundance to total bacterial genome abundance) |
| --- | --- |
| *OTU_M_H_C (related to Alkaliphilus halophilus)* | *Alkaliphilus*  Experiment 1: <0.4% all reactors  Experiment 2: <0.2% all reactors Experiment 3: <0.1% all reactors |
| *Tepidanaerobacter acetatoxydans* | *Tepidanaerobacter* Experiment 1: <0.2% all reactors Experiment 2: <0.1% all reactors  Experiment 3: 0.31% R_conti_ |
| *Clostridium ultunense* | Experiment 1: N/A  Experiment 2: N/A  Experiment 3: N/A |
| *Thermacetogenium phaeum* | *Thermoanaerobacterales*  Experiment 1: 1.6% R_conti_,A; 2.1% R_conti_,B; 1.3% Rdisco  Experiment 2: ~2% both R_conti_+R_disco_ Experiment 3: 2.18% R_conti_; 0.28% R_disco_,A; 0.33% R_disco_,B |
| *Pseudothermotoga lettingae* | *Thermotogaceae_Mesotoga* Experiment 3: 1.74% R_conti_; 0.5% R_disco_,A; 0.74% R_disco_,B  Experiment 2: 5.2-8.7% R_conti_; R_disco_ 0.6-0.9%  Experiment 1: 2.2% R_conti_,A; 4.1% R_conti_,B; 1.5% R_disco_ |
| *Syntrophaceticus schinkii* | See *Thermoanaerobacterales* |

### A.2.2.5 Inference of biological network associations using CoNet

There are 12 described genera of syntrophic VFA oxidizers: the butyric acid oxidizers *Syntrophomonas, Syntrophus, Syntrophothermus* [5] and *Thermosyntropha* [6], the propionic acid oxidizers *Smithella, Syntrophobacter* and *Pelotomaculum* [5]) and acetic acid oxidizers *Pseudothermotoga, Thermacetogenium, Clostridium, Syntrophaceticus* and *Tepidanaerobacter* [7] from which only *Syntrophobacter*, *Pelotomaculum* and *Syntrophomonas* were found in our samples.

Mutual exclusion was chosen based on the fact that throughout all experiments, almost all VFAs were consumed at any time point. Therefore, an OTU inside a functional group most likely increases in relative abundance at the cost of another OTU inside the same functional group competing for the same substrate. However, the detection of this relationship can be hampered by differences in ploidy, biomass yields and cellular biomass between the competing species. Sufficient physiological information on the detected bacteria to correct for these potential differences is not available. Furthermore, the correlations needed to be calculated based on a low sample size (n=21).

The weights of statistically significant (Pearson correlation, threshold=0.05) mutual exclusions of OTUs to *Syntrophomonas* (Table A.4), *Syntrophobacter* (Table A.5) and both *Syntrophomonas* and *Syntrophobacter* (Table A.6) were calculated using the CoNet App (v 1.1.1 beta) inside Cytoscape (v 3.6.0). These statistically significant mutual exclusions are a first hint on the function of the listed OTUs in our reactors. OTUs in Table A.4 are more likely butyric acid oxidizers while OTUs in Table A.5 are more likely propionic acid oxidizers. Table A.6 shows OTUs that appear in both Table A.4 and A.5. These OTUs might be able to degrade both acids but they might also just negatively correlate for other reasons with *Syntrophobacter* or *Syntrophomonas* or both of them.

Table A.4 Network analysis for *Syntrophomonas*: Pearson correlation (threshold=0.05) for all samples (n=23)

| **Phylum** | **Class** | **Order** | **Family** | **Genus** | **Weight** |
| --- | --- | --- | --- | --- | --- |
| p_Bacteroidetes | c_Sphingobacteriia | o_Sphingobacteriales | f_WCHB169 | g_G35D8 | -0.52 |
| p_Proteobacteria | c_Gammaproteobacteria | o_Xanthomonadales | f_Xanthomonadaceae | g_Stenotrophomonas | -0.48 |
| p_Bacteroidetes | c_Sphingobacteriia | o_Sphingobacteriales | f_Chitinophagaceae | Other | -0.43 |
| p_SHA109 | c_ | o_ | f_ | g_ | -0.43 |
| p_Proteobacteria | c_Betaproteobacteria | o_Burkholderiales | f_Comamonadaceae | g_Comamonas | -0.41 |
| p_CandidatedivisionWS6 | c_ | o_ | f_ | g_ | -0.36 |
| p_Armatimonadetes | c_ | o_ | f_ | g_ | -0.35 |
| p_CandidatedivisionSR1 | c_ | o_ | f_ | g_ | -0.34 |
| p_Firmicutes | c_Clostridia | o_D8A2 | f_MRE50b23 | g_MRE50b23 | -0.34 |
| p_Actinobacteria | c_Actinobacteria | o_Corynebacteriales | f_Corynebacteriaceae | g_Corynebacterium1 | -0.31 |
| p_Firmicutes | c_Clostridia | o_Clostridiales | f_Gracilibacteraceae | g_Lutispora | -0.31 |
| p_Firmicutes | c_Clostridia | o_Clostridiales | f_FamilyXIII | g_Anaerovorax | -0.30 |
| p_Bacteroidetes | c_Bacteroidia | o_Bacteroidales | f_Rikenellaceae | g_Blvii28wastewatersludgegroup | -0.27 |
| p_Spirochaetae | c_Spirochaetes | o_Spirochaetales | f_Leptospiraceae | g_uncultured | -0.26 |
| p_Synergistetes | c_Synergistia | o_Synergistales | f_Synergistaceae | g_Thermovirga | -0.26 |
| p_Proteobacteria | c_Alphaproteobacteria | o_Rhodobacterales | f_Rhodobacteraceae | g_Paracocccus | -0.24 |
| p_Chloroflexi | c_WCHB150 | o_ | f_ | g_ | -0.24 |
| p_Proteobacteria | c_Epsilonproteobacteria | o_Campylobacterales | f_Helicobacteraceae | g_Wolinella | -0.23 |
| p_Firmicutes | c_Clostridia | o_Clostridiales | f_Clostridiaceae1 | g_Proteiniclasticum | -0.23 |
| p_Bacteroidetes | c_Bacteroidia | o_Bacteroidales | f_Rikenellaceae | g_vadinBC27wastewatersludgegroup | -0.23 |
| p_Proteobacteria | c_Alphaproteobacteria | o_Rhizobiales | f_Rhodobiaceae | g_Parvibaculum | -0.20 |
| p_Chloroflexi | c_Anaerolineae | o_Anaerolineales | f_Anaerolineaceae | g_uncultured | -0.19 |
| p_Synergistetes | c_Synergistia | o_Synergistales | f_Synergistaceae | g_uncultured | -0.18 |
| p_Firmicutes | c_Bacilli | o_Bacillales | f_Bacillaceae | g_Bacillus | -0.18 |
| p_Firmicutes | c_Clostridia | o_Clostridiales | f_Peptococcaceae | g_Cryptanaerobacter | -0.17 |
| p_Bacteroidetes | c_vadinHA17 | o_vadinHA17 | f_WCHB153 | g_TSI14 | -0.16 |
| p_Tenericutes | c_Mollicutes | o_NB1n | f_RFN82 | g_ | -0.16 |
| p_Firmicutes | c_Clostridia | o_Clostridiales | f_Gracilibacteraceae | Other | -0.15 |
| p_Firmicutes | c_Clostridia | o_Clostridiales | Other | Other | -0.15 |
| p_Chloroflexi | c_Anaerolineae | o_Anaerolineales | f_Anaerolineaceae | g_Pelolinea | -0.15 |
| p_Firmicutes | c_Clostridia | o_Clostridiales | f_Peptococcaceae | g_uncultured | -0.14 |
| p_Proteobacteria | c_Epsilonproteobacteria | o_Campylobacterales | f_Helicobacteraceae | g_RsM59termitegroup | -0.13 |
| p_Firmicutes | c_Clostridia | o_Clostridiales | f_Ruminococcaceae | Other | -0.12 |
| p_Parcubacteria | c_ | o_ | f_ | g_ | -0.11 |
| p_Firmicutes | c_OPB54 | o_Hydrogenisporales | f_MBA03 | g_ | -0.10 |
| p_Atribacteria | c_AtribacteriaIncertaeSedis | o_UnknownOrder | f_Caldatribacteriaceae | g_ | -0.10 |
| p_Firmicutes | c_Clostridia | o_Thermoanaerobacterales | f_FamilyIII | g_Tepidanaerobacter | -0.08 |
| p_Proteobacteria | c_Deltaproteobacteria | o_Desulfovibrionales | f_Desulfovibrionaceae | g_Desulfovibrio | -0.07 |
| p_Bacteroidetes | c_Bacteroidia | o_Bacteroidales | f_Porphyromonadaceae | g_Proteiniphilum | -0.07 |
| p_Thermotogae | c_Thermotogae | o_Thermotogales | f_Thermotogaceae | g_Mesotoga | -0.07 |
| p_Lentisphaerae | c_Oligosphaeria | o_Oligosphaerales | f_Oligosphaeraceae | g_ | -0.06 |

Table A.5 Network analysis for *Syntrophobacter*: Pearson correlation (threshold=0.05) for all samples (n=23)

| **Phylum** | **Class** | **Order** | **Family** | **Genus** | **Weight** |
| --- | --- | --- | --- | --- | --- |
| p_Tenericutes | c_Mollicutes | o_Acholeplasmatales | f_Acholeplasmataceae | g_Acholeplasma | -0.64 |
| p_Synergistetes | c_Synergistia | o_Synergistales | f_Synergistaceae | g_Aminobacterium | -0.62 |
| p_CandidatedivisionSR1 | c_ | o_ | f_ | g_ | -0.58 |
| p_Bacteroidetes | c_Bacteroidia | o_Bacteroidales | f_Rikenellaceae | g_RikenellaceaeRC9gutgroup | -0.58 |
| p_Firmicutes | c_Clostridia | o_Clostridiales | f_Peptococcaceae | g_Pelotomaculum | -0.56 |
| p_Tenericutes | c_Mollicutes | o_NB1n | f_RFN82 | g_ | -0.54 |
| p_Firmicutes | c_Clostridia | o_Clostridiales | f_Ruminococcaceae | Other | -0.49 |
| p_Bacteroidetes | c_Sphingobacteriia | o_Sphingobacteriales | f_WCHB169 | g_G35D8 | -0.43 |
| p_Firmicutes | c_Clostridia | o_Clostridiales | f_Peptococcaceae | Other | -0.42 |
| p_Firmicutes | c_Clostridia | o_Thermoanaerobacterales | f_SRB2 | g_ | -0.37 |
| p_Firmicutes | c_Clostridia | o_Clostridiales | f_Syntrophomonadaceae | g_uncultured | -0.30 |
| p_Proteobacteria | c_Epsilonproteobacteria | o_Campylobacterales | f_Helicobacteraceae | g_Wolinella | -0.27 |
| p_Firmicutes | c_OPB54 | o_Hydrogenisporales | f_MBA03 | g_ | -0.26 |
| p_Firmicutes | c_Clostridia | o_Clostridiales | f_FamilyXIII | g_FamilyXIIIUCG002 | -0.25 |
| p_Firmicutes | c_Clostridia | o_Clostridiales | f_Gracilibacteraceae | g_Lutispora | -0.19 |
| p_Firmicutes | c_Clostridia | o_Clostridiales | f_Ruminococcaceae | g_Fastidiosipila | -0.17 |
| p_Bacteroidetes | c_vadinHA17 | o_vadinHA17 | f_SHA94 | g_SHA94 | -0.13 |
| p_Firmicutes | c_Clostridia | o_Thermoanaerobacterales | f_FamilyIII | g_Tepidanaerobacter | -0.10 |

Table A.6 Network analysis for *Syntrophobacter* and *Syntrophomonas*: Pearson correlation (treshold=0.05) for all samples (n=23)

| **Phylum** | **Class** | **Order** | **Family** | **Genus** | **Weight** | **Phylum** |
| --- | --- | --- | --- | --- | --- | --- |
| g_Syntrophobacter | p_Bacteroidetes | c_Sphingobacteriia | o_Sphingobacteriales | f_WCHB169 | g_G35D8 | -0.43 |
| g_Syntrophomonas | p_Bacteroidetes | c_Sphingobacteriia | o_Sphingobacteriales | f_WCHB169 | g_G35D8 | -0.52 |
| g_Syntrophobacter | p_Firmicutes | c_Clostridia | o_Clostridiales | f_Gracilibacteraceae | g_Lutispora | -0.19 |
| g_Syntrophomonas | p_Firmicutes | c_Clostridia | o_Clostridiales | f_Gracilibacteraceae | g_Lutispora | -0.31 |
| g_Syntrophobacter | p_Firmicutes | c_Clostridia | o_Thermoanaerobacterales | f_FamilyIII | g_Tepidanaerobacter | -0.10 |
| g_Syntrophomonas | p_Firmicutes | c_Clostridia | o_Thermoanaerobacterales | f_FamilyIII | g_Tepidanaerobacter | -0.08 |
| g_Syntrophobacter | p_Proteobacteria | c_Epsilonproteobacteria | o_Campylobacterales | f_Helicobacteraceae | g_Wolinella | -0.27 |
| g_Syntrophomonas | p_Proteobacteria | c_Epsilonproteobacteria | o_Campylobacterales | f_Helicobacteraceae | g_Wolinella | -0.23 |
| g_Syntrophobacter | p_Firmicutes | c_Clostridia | o_Clostridiales | f_Ruminococcaceae | Other | -0.49 |
| g_Syntrophobacter | p_Firmicutes | c_Clostridia | o_Clostridiales | f_Peptococcaceae | Other | -0.42 |
| g_Syntrophomonas | p_Bacteroidetes | c_Sphingobacteriia | o_Sphingobacteriales | f_Chitinophagaceae | Other | -0.43 |
| g_Syntrophomonas | p_Firmicutes | c_Clostridia | o_Clostridiales | f_Gracilibacteraceae | Other | -0.15 |
| g_Syntrophomonas | p_Firmicutes | c_Clostridia | o_Clostridiales | Other | Other | -0.15 |
| g_Syntrophomonas | p_Firmicutes | c_Clostridia | o_Clostridiales | f_Ruminococcaceae | Other | -0.12 |

### A.2.2.6 Specialists for continuous and discontinuous feeding, generalists and most abundant members of the bacterial community

Table A.7 summarizes bacterial OTUs that have drawn our attention, because they occur in all reactors in all experiments (“generalists”), because they are more abundant in the continuous fed reactors than in the discontinuously fed reactors in each experiment (“specialist R_conti_”) and vice versa (“specialist R_disco_”) or because they belong to the five most abundant bacterial OTUs in a reactor (“Top 5”).

Table A.7 Specialists for continuous and discontinuous feeding, generalists and most abundant members of the bacterial community

| **OTU** | **generalist** | **Specialist R_conti_** | **Specialist R_disco_** | **Top 5** | **Weight (Pearsson, p<0.05)** | **Physiological characterization of related taxa** |
| --- | --- | --- | --- | --- | --- | --- |
| **p__Bacteroidetes;**c__Bacteroidia;o__Bacteroidales;  f__Rikenellaceae;g__Blvii28 wastewater-sludge group |  |  |  | ●● | -0.27 (*Syntrophomonas*) | *Acetobacteroides hydrogenigenes* gen. nov., sp. nov.: carbohydrates degrader, not degradation of propionic or butyric acid in methanogenic co-culture [8] |
| **p__Bacteroidetes;**c__Bacteroidia;o__Bacteroidales;  f__Rikenellaceae;**;**g__ Rikenellaceae RC9 gut group |  |  |  | ●● | -0.58 (*Syntrophobacter*) |  |
| **p__Bacteroidetes;**c__Sphingobacteriia;o__Sphingobacteriales; f__ST-12K33 |  |  |  | ● | NA | Sphingobacteriales spp. dominated high solid rape straw AD [9] |
| **p__Bacteroidetes;**c__vadinHA17;o__vadinHA17 |  |  |  |  | -0.16 (*Syntrophomonas*), -0.13 (*Syntrophobacter*) |  |
| **p__Cloacimonetes;**c__W27 | ● |  |  | ●●●●● | NA | Genome of Candidatus *Cloacamonas acidaminovorans* contains all necessary genes for propionic acid oxidation via methylmalonyl-CoA [10] |
| **p__Elusimicrobia;**c__Elusimicrobia;o__Lineage I (Endomicrobia) |  |  | ● | ●●● | NA |  |
| **p__Firmicutes;**c__Clostridia;o__Clostridiales;  f__Family XIII;g__Family XIII UCG-002 |  |  |  | ● | -0.25 (*Syntrophobacter*) |  |
| **p__Firmicutes;**c__Clostridia;o__Clostridiales; f__Syntrophomonadaceae;g__Syntrophomonas | ● |  |  | ●●●●● | NA | syntrophic butyric acid oxidizer [11] |
| **p__Firmicutes;**c__Clostridia;o__Clostridiales; f__uncultured |  |  |  | ●● | -0.15 (*Syntrophomonas*) |  |
| **p__Nitrospirae;**c__Nitrospira;o__Nitrospirales;  f__Nitrospiraceae;g__uncultured |  | ● |  |  | NA | Thermophilic sulfate reducers, hydrogenotrophic or chemoorganoheterotrophic [12]. Enriched in anaerobic digester with tylosin (antimicrobial) addition [13]. |
| **p__Proteobacteria;**c__Deltaproteobacteria;o__Desulfovibrionales;  f__Desulfovibrionaceae;g__Desulfovibrio |  | ● |  |  | -0.07 (*Syntrophomonas*) |  |
| **p__Proteobacteria;**c__Deltaproteobacteria;o__Syntrophobacterales;  f__Syntrophobacteraceae;g__Syntrophobacter | ● |  |  | ●●●●● | NA | propionic acid oxidizer [14] |
| **p__Proteobacteria;**c__Betaproteobacteria;o__Burkholderiales;  f__Comamonadaceae;g__Brachymonas |  |  |  | ● | NA |  |
| **p__Spirochaetae**;c__Spirochaetes;o__Spirochaetales;  f__Spirochaetaceae;g__uncultured | ● |  |  | ●●●● | NA |  |
| **p__Spirochaetae**;c__Synergistia;o__Synergistales;  f__Synergistaceae;g__Aminobacterium |  |  |  |  | -0.62 (*Syntrophobacter*) |  |
| **p__Spirochaetae**;c__Synergistia;o__Synergistales;  f__Synergistaceae;g__Thermovirga | ● |  |  | ●●●● | -0.26 (*Syntrophomonas*) | *Thermovirga lienii* gen. nov., sp. nov: amino acid fermenter under thermophilic conditions [15] |
| **p__Spirochaetae**;c__Synergistia;o__Synergistales;  f__Synergistaceae;g__uncultured |  |  |  | ●● | -0.18 (*Syntrophomonas*) |  |
| **p__Thermotogae;**c__Thermotogae;o__Thermotogales;  f__Thermotogaceae;g__Mesotoga |  | ● |  | ●● | -0.07 (*Syntrophomonas*) | *Mesotoga prima* gen. nov., sp. nov.: sugar and amino acid degrader [16] |
| Analysis only includes samples at the end of the training phase.  generalists: relative genome abundance in all reactor samples >0.7% in all reactors.  specialist R_conti_: relative genome abundance in R_conti_ > R_disco_ for all three experiments including technical replicates.  specialist R_disco_: relative genome abundance in R_disco_ > R_conti_ for all three experiments including technical replicates.  Top 5: Number of points = number of reactors where taxa is under 5 most abundant bacteria  Weight determined with CoNet analyzer. A negative weight = mutual exclusion. If OTU is mutually exclusive to Syntrophomonas, then potentially butyric acid oxidizer, if to Syntrophobacter or a propionic acid oxidizer. | | | | | | |

## A.2.3 Methanogenic community structure

Figure A.15 shows the composition of methanogens for all sampling points of the three reactor experiments.

### A.2.3.1 T-RFLP DNA

Figure A.15 T-RFLP profiles of the methanogenic community on DNA level. T-RFs that could not be assigned are labelled as "Other". ”Ambiguous on family level” stands for *Methanocorpusculaceae, Methanomicrobiaceae*, *Methanoregulaceae*, and *Methanospirillaceae* which cannot be distinguished by our T-RFLP analysis.

### A.2.3.2 T-RFLP cDNA

Figure A.16 shows the relative abundance of *mcrA* cDNA (synthesized from mRNA) of the methanogens of all three reactor experiments for all sampling points. Figure A.17 shows a comparison between all experiments of the relative abundance of *mcrA* cDNA before and after a feeding event.

Figure A.16 T-RFLP analysis of the methanogenic community on cDNA level. T-RFs that could not be assigned are labelled as "Other". ”Ambiguous on family level” stands for *Methanocorpusculaceae, Methanomicrobiaceae*, *Methanoregulaceae*, and *Methanospirillaceae* which cannot be distinguished by our T-RFLP analysis.

Figure A.17 T-RFLP analysis of the methanogenic community on cDNA level before and after discontinuous feeding. Comparison of all experiments. T-RFs that could not be assigned are labelled as "Other". ”Ambiguous on family level” stands for *Methanocorpusculaceae, Methanomicrobiaceae*, *Methanoregulaceae*, and *Methanospirillaceae* which cannot be distinguished by our T-RFLP analysis.

## A.2.4 Ratio of bacteria to archaea

As mentioned in the manuscript, the primers used for 16S rRNA gene sequencing do not provide the same high coverage for archaea as for bacteria. Therefore, the ratio of bacteria to all prokaryotes determined based on the sequencing data can only qualify as maximum estimate for the relative abundance of bacteria. This estimate for the relative abundance of bacteria over total prokaryotes on genome basis was determined to 14±8% (average±SD) based on all three experiments (see Figure A.18).

Figure A.18 Relative abundance of bacteria over total prokaryotes based on 16S rRNA sequencing results. Genome abundances are derived by correcting for the average bacterial 16S rRNA operon copy number per genome for each sample.

## A.2.5 [2-^13^C]-acetate labelling experiment

Except for one outlier, the ratio of ^13^C-labelled CO_2_ to ^13^C-labelled CH_4_ was much lower than 1 (Table A.8) which is an indicator that the major acetic acid utilization occurs via acetoclastic methanogenesis, and not via syntrophic acetic acid oxidation [17]. This is in accordance together with the low abundance of known SAOB found by amplicon sequencing. Furthermore, there was no major difference between R_conti_ and R_disco_.

Table A.8 Ratio of ^13^C-labelled acetate to ^13^C-labelled CO_2_ in batch experiment for Experiment 1

| Batch sample | ^13^CO_2_/^13^CH_4_ in gas phase | ^13^CO_2_/^13^CH_4_ in gas and liquid phase |
| --- | --- | --- |
| R_conti,A__t=50d (a) | N/A | N/A |
| R_conti,A__t=50d (b) | 0.027 | 0.031 |
| R_conti,A__t=50d (c) | 1.07 | 1.418 |
|  |  |  |
| R_conti,B__t=50d (a) | 0.021 | 0.027 |
| R_conti,B__t=50d (b) | 0.033 | 0.045 |
| R_conti,B__t=50d (c) | 0.021 | 0.028 |
|  |  |  |
| R_disco__t=50d (a) | 0.029 | 0.040 |
| R_disco__t=50d (b) | 0.023 | 0.031 |
| R_disco__t=50d (c) | 0.022 | 0.030 |
| (a),(b),(c) stand for biological replicates R_conti,A__t=50d (a) technical problem |  |  |

## A.2.6 ADM1 Model

### A.2.6.1 Growth kinetic parameters

The growth kinetic parameters for the simulations were chosen based on the following considerations:

*Microbial biomass yields*

1. Y_ac1_ = Y_ac2_ = 0.033 gCOD_X_ gCOD_S_^‑1^ based on the apparent microbial biomass yield of 0.02 g_X_ g_S_^‑1^ from a *Methanosaeta*-enriched chemostat [18] corrected for the decay rate of 0.02 d^-1^ [19] and converted to COD by assuming 1.416 gCOD gVS^-1^ [20].
2. Y_pro_ = 0.012 gCOD_X_ gCOD_S_^‑1^ and Y_but_ and 0.012 gCOD_X_ gCOD_S_^‑1^ based on the minimum biomass concentrations determined in chapter A.2.1.4 and the default microbial biomass decay rate 0.02 d^-1^ [19].
3. Y_h2_ = 0.032 gCOD_X_ gCOD_S_^‑1^ based on the average total microbial biomass concentration of Experiment 1 of 0.9 gVS L^-1^, which equals 1.3 gCOD_X_ L^-1^ assuming 1.416 gCOD gVS^-1^ [20], minus the microbial biomass concentrations of acetic, propionic and butyric acid degraders based on the yields above.

*Half saturation constants*

1. K_S,pro_ = 0.1 gCOD_S_ L^-1^, K_but_ = 0.2 gCOD_S_ L^-1^, K_h2_ = 0.2 gCOD_S_ L^-1^  based on [19].
2. K_S,ac1_ = 0.32 gCOD_S_ L^-1^ and K_S,ac2_ = 0.09 gCOD_S_ L^-1^ based on [21].

*Maximum specific substrate uptake rates*

1. k_m,h2_ = 35 gCOD_S_ gCOD_X_^‑1^ d^-1^ based on [19].
2. k_m,pro_ = 30 and k_m,but_ = 25 gCOD_S_ gCOD_X_^‑1^ d^-1^ to avoid propionic and butyric acid accumulation.
3. k_m,ac1_ = 14.5 gCOD_S_ gCOD_X_^‑1^ d^-1^ based on the minimum rate of 8.7 gCOD_ac_ gCOD_Xac_d^-1^ (Section A.2.1.4) divided by an inhibition factor of 0.6 which follows from the default free ammonia inhibition [19].
4. k_m,ac2_ = 20 gCOD_S_ gCOD_X_^‑1^ d^-1^ which is the maximum value leading to simulation results close to our experimental data considering pH and total VFA concentrations after the disturbances as well as population dynamics of acetoclastic methanogens.

*pH inhibition*

1. pH_u_ac1 (upper limit) = 5 and pH_l_ac1 (lower limit) = 4.5 based on the strong resistance of *Methanosarcina* against ammonia inhibition [22]
2. pH_u_ac2 (upper limit) = 7 based on default [19] and pH_l_ac2 = 6.3 which is the highest value leading to simulation results close to our experimental data considering pH and total VFA concentrations after the disturbances as well as population dynamics of acetoclastic methanogens.

*Discussion*

Assuming identical microbial biomass yields of both acetoclastic methanogens is justified because our experimental absolute microbial biomass concentrations do not change after a shift from *Methanosarcina* (X_ac1_) to *Methanosaeta* (X_ac2_) and vice versa. However, in literature, higher yield values for *Methanosarcina* compared to *Methanosaeta* have been reported [18]. Such differences might have been unnoticed in our experimental data due to biomass concentration measurement errors. A higher yield value for *Methanosarcina* would result in a higher biomass concentration of *Methanosarcina* and accordingly, lower specific maximum substrate uptake rates could be chosen.

All of our microbial biomass yields are low compared to default ADM1 yields (see Table 1), however, they are consistent with maximum yields previously determined based on the Gibbs energy dissipation method [2]. For the conversion of propionic acid to methane, the total maximum microbial biomass yield based on our assumptions is 0.15 Cmol_X_  mol_S_^-1^ compared to 0.12 Cmol_X_  mol_S_^-1^ for the Gibbs energy dissipation method [2] while it is 0.31 Cmol_X_  mol_S_^-1^ for default ADM1 parameter values. For the conversion of butyric acid to methane, the total maximum microbial biomass yield based on our assumptions is 0.21 Cmol_X_  mol_S_^-1^ and 0.16 Cmol_X_  mol_S_^-1^ for the Gibbs energy dissipation method [2] while it is 0.52 Cmol_X_  mol_S_^-1^ for default ADM1 parameter values. For the conversion of acetic acid to methane, the total maximum microbial biomass yield based on our assumptions is 0.063 Cmol_X_  mol_S_^-1^ and 0.06 Cmol_X_  mol_S_^-1^ for the Gibbs energy dissipation method [2] while it is 0.096 Cmol_X_  mol_S_^-1^ for default ADM1 parameter values.

The assumptions about individual microbial biomass concentrations above were necessary because they could not be solely based on T-RFLP and amplicon sequencing data for several reasons. First of all, since not all VFA oxidizing bacteria are known in our system, X_pro_ and X_but_ cannot be determined from experimental data. X_h2_ and X_ac_ could have been estimated based on the total microbial biomass, the ratio of archaea to bacteria (Section A.2.4) and the relative methanogenic abundances by T-RFLP. However, we did not do this because our experimentally determined ratio of archaea to bacteria led to unrealistic results concerning substrate uptake rates of bacteria. For R_disco_ in Experiment 1 on day 58, bacteria make up 10.5% on genome level of total prokaryotes based on 16S rRNA gene amplicon sequencing. For a total microbial biomass concentration of 1.2 gCOD L^-1^, the resulting 0.126 gCOD L^-1^ would not be enough microbial biomass to explain the experimentally determined propionic and butyric acid uptake rates (Section A.2.1.4) without exceeding the maximum electron transfer rate of 51 gCOD_S_ gCOD_X_^‑1^ d^-1^. Besides amplicon sequencing biases for example because of primer biases, important reasons might be differences in ploidy (number of genomes per cell), as well as microbial biomass and COD per cell.

### A.2.6.2 Detailed simulation results of Experiment 1

The detailed ADM1 simulation results of Experiment 1 are shown in Figure A.19 (R_disco_), Figure A.20 (R_conti_,_B_) and Figure A.21.

(a)

 (b)

******

Figure A.19 ADM1 simulation results for Experiment 1. Disturbance on day 64 d. (a) Total VFA concentration and (b) acetic acid concentration.

Figure A.20 ADM1 simulation results for total microbial biomass concentrations for Experiment 1. Disturbance on day 64.

#### Reason for higher pH in R_disco_

To show that the higher pH in R_disco_ compared to R_conti_ in experiment and simulation is not a result of different VFA concentrations, we repeated the simulation of Experiment 1 with increased maximum specific substrate uptake rates and decreased half saturation constants to reach near zero VFA concentrations (detailed parameter values see Additional File 2).

This led to maximum pH values of 7.53 and 7.28 for R_disco_ and R_conti_ (Figure A.19a), respectively, while VFA concentrations were negligible in both simulations (<6 mg COD_VFA_ L^-1^, Figure A.19b). This shows that the higher pH in R_disco_ compared to R_conti_ at the end of each feeding interval is not due to a different VFA concentration. Since both reactors were fed with the same medium, the difference in pH is most likely due to the CO_2_ outgassing during a feeding event (Figure A.21c).

(a)

(b)

(c)

Figure A.21 ADM1 simulation for Experiment 1 with increased maximum specific substrate uptake rates (51 gCOD_ac_ L^‑1^ d^-1^) and decreased half-saturation constants (0.001 gCOD L^-1^) to compare the maximum pH for both feeding regimes in Experiment 1 independent of residual VFA anions. (a) pH values, (b) VFA concentrations and (c) dissolved inorganic carbon concentrations.

### A.2.6.3 Simulation results of Experiment 2 and Experiment 3

Experiment 2 (Figure A.22) and Experiment 3 (Figure A.23) were simulated using the same parameter set as for Experiment 1.

(a)

(b)

(c)

(d)

Figure A.22 ADM1 simulations for Experiment 2 (a) pH, (b) total VFA concentration, (c) total microbial biomass concentration and (d) acetoclastic methanogens.

(a)

(b)

(c)

(d)

Figure A.23 ADM1 simulations for Experiment 3 (a) pH, (b) total VFA concentration, (c) total microbial biomass concentration and (d) acetoclastic methanogens.

### A.2.6.4 Discontinuously fed maize silage reactor

Applying the microbial parameter set of Experiment 1 to maize silage digesting reactors showed complete dominance of *Methanosaeta* for continuous feeding (Figure A.24a) versus a shift from *Methanosaeta* to *Methanosarcina* for discontinuous feeding (Figure A.24b). It took 64 d to reach a relative abundance of 32.3% for *Methanosarcina* which is the relative abundance of *Methanosarcina* at the end of the training phase in the simulation of R_disco_ in Experiment 1. The initial concentration of *Methanosarcina* was set to 0.2 gCOD L^-1^, which could be easily reached in practice by replacing 5% of the digester content with a digester content rich in *Methanosarcina*.

(b)

(a)

Figure A.24 ADM1 simulation of a digester fermenting maize silage using the same growth kinetic parameter values of Experiment 1 (a) continuous feeding and (b) discontinuous feeding.

**References**

1. Heijnen JJ. Bioenergetics of Microbial GrowthMicrobial Growth. In: Flickinger MC, Drew SW, editors. Encyclopedia of Bioprocess Technology. Hoboken, NJ, USA: John Wiley & Sons, Inc.; 2002. p. 267–91. doi:10.1002/0471250589.ebt026.

2. Kleerebezem R, Van Loosdrecht MCM. A generalized method for thermodynamic state analysis of environmental systems. Crit Rev Environ Sci Technol. 2010;40:1–54. doi:10.1080/10643380802000974.

3. Bardou P, Mariette J, Escudié F, Djemiel C, Klopp C. Jvenn: An interactive Venn diagram viewer. BMC Bioinformatics. 2014;15:293. doi:10.1186/1471-2105-15-293.

4. Westerholm M, Müller B, Singh A, Karlsson Lindsjö O, Schnürer A. Detection of novel syntrophic acetate-oxidizing bacteria from biogas processes by continuous acetate enrichment approaches. Microb Biotechnol. 2018;11:680–93. doi:10.1111/1751-7915.13035.

5. Worm P, Koehorst JJ, Visser M, Sedano-Núñez VT, Schaap PJ, Plugge CM, et al. A genomic view on syntrophic versus non-syntrophic lifestyle in anaerobic fatty acid degrading communities. Biochim Biophys Acta - Bioenerg. 2014;1837:2004–16. doi:10.1016/j.bbabio.2014.06.005.

6. Sieber JR, McInerney MJ, Müller N, Schink B, Gunsalus RP, Plugge CM. Methanogens: Syntrophic Metabolism. In: Biogenesis of Hydrocarbons. Cham: Springer International Publishing; 2018. p. 1–31. doi:10.1007/978-3-319-53114-4_2-1.

7. Müller B, Sun L, Westerholm M, Schnürer A. Bacterial community composition and fhs profiles of low- and high-ammonia biogas digesters reveal novel syntrophic acetate-oxidising bacteria. Biotechnol Biofuels. 2016;9:48. doi:10.1186/s13068-016-0454-9.

8. McIlroy SJ, Saunders AM, Albertsen M, Nierychlo M, McIlroy B, Hansen AA, et al. MiDAS: the field guide to the microbes of activated sludge. Database. 2015;2015:bav062. doi:10.1093/database/bav062.

9. Tian JH, Pourcher AM, Bureau C, Peu P. Cellulose accessibility and microbial community in solid state anaerobic digestion of rape straw. Bioresour Technol. 2017;223:192–201. doi:10.1016/j.biortech.2016.10.009.

10. Pelletier E, Kreimeyer A, Bocs S, Rouy Z, Gyapay G, Chouari R, et al. “Candidatus Cloacamonas acidaminovorans”: Genome sequence reconstruction provides a first glimpse of a new bacterial division. J Bacteriol. 2008;190:2572–9.

11. Sousa DZ, Smidt H, Madalena Alves M, Stams AJM. *Syntrophomonas zehnderi* sp. nov., an anaerobe that degrades long-chain fatty acids in co-culture with *Methanobacterium formicicum*. Int J Syst Evol Microbiol. 2007;57:609–15.

12. Daims H. The Family *Nitrospiraceae*. In: The Prokaryotes. Berlin, Heidelberg: Springer Berlin Heidelberg; 2014. p. 733–49. doi:10.1007/978-3-642-38954-2_126.

13. Shimada T, Li X, Zilles JL, Morgenroth E, Raskin L. Effects of the antimicrobial tylosin on the microbial community structure of an anaerobic sequencing batch reactor. Biotechnol Bioeng. 2011;108:296–305.

14. Boone DR, Bryant MP. Propionate-Degrading Bacterium , *Syntrophobacter wolinii* sp . nov . gen . nov ., from Methanogenic Ecosystems. Appl Environ Microbiol. 1980;40:626–32. http://www.ncbi.nlm.nih.gov/pmc/articles/PMC291629/.

15. Dahle H, Birkeland NK. *Thermovirga lienii* gen. nov., sp. nov., a novel moderately thermophilic, anaerobic, amino-acid-degrading bacterium isolated from a North Sea oil well. Int J Syst Evol Microbiol. 2006;56:1539–45.

16. Nesbø CL, Bradnan DM, Adebusuyi A, Dlutek M, Petrus AK, Foght J, et al. *Mesotoga prima* gen. nov., sp. nov., the first described mesophilic species of the Thermotogales. Extremophiles. 2012;16:387–93.

17. Fotidis I a., Karakashev D, Angelidaki I. The dominant acetate degradation pathway/methanogenic composition in full-scale anaerobic digesters operating under different ammonia levels. Int J Environ Sci Technol. 2014;11:2087–94. doi:10.1007/s13762-013-0407-9.

18. Conklin A, Stensel HD, Ferguson J. Growth kinetics and competition between *Methanosarcina* and *Methanosaeta* in mesophilic anaerobic digestion. Water Environ Res. 2006;78:486–96.

19. Rosen C, Jeppsson U. Aspects on ADM1 Implementation within the BSM2 Framework. Lund, Sweden; 2006. http://www.iea.lth.se/publications/Reports/LTH-IEA-7224.pdf.

20. Hoover SR, Porges N. Assimilation of Dairy Wastes by Activated Sludge: II. The Equation of Synthesis and Rate of Oxygen Utilization. Sewage Ind Waste. 1952;24:306–12.

21. Straub AJ, Conklin ASQ, Ferguson JF, Stensel HD. Use of the ADM1 to investigate the effects of acetoclastic methanogen population dynamics on mesophilic digester stability. Water Sci Technol. 2006;54:59–66.

22. De Vrieze J, Hennebel T, Boon N, Verstraete W. *Methanosarcina*: The rediscovered methanogen for heavy duty biomethanation. Bioresour Technol. 2012;112:1–9. doi:10.1016/j.biortech.2012.02.079.

1. * Corresponding author: Fabian Bonk, [fabian.bonk@ufz.de](mailto:fabian.bonk@ufz.de), +49 341 2351315
    [↑](#footnote-ref-1)
