# Supplementary material for: Intermittent fasting for microbes: how discontinuous feeding increases functional stability in anaerobic digestion
Source: Biotechnol Biofuels. 2018 Oct 6;11:274. doi: 10.1186/s13068-018-1279-5 (PMC6173896; doi:10.1186/s13068-018-1279-5)
Supplement: Supplementary file 3 — Additional file 3. Bacterial community composition. Zoomable pie chart using the visualization tool Krona. [file 13068_2018_1279_MOESM3_ESM.html]

Javascript must be enabled to view this page.

magnitude

Exp.1; Rconti,A; t=0 d
Exp.1; Rconti,B; t=0 d
Exp.1; Rdisco; t=0 d
Exp.1; Rconti,B; t=58 d
Exp.1; Rdisco; t=58 d
Exp.1; Rconti,A; t=64 d
Exp.1; Rconti,B; t=64 d
Exp.1; Rdisco; t=64 d
Exp.1; Rconti,B; t=71 d
Exp.1; Rdisco; t=71 d
Exp.1; Rdisco; t=80 d
Exp.2; Rconti; t=0 d
Exp.2; Rdisco; t=0 d
Exp.2; Rconti average; t=54 d
Exp.2; Rdisco average; t=54 d
Exp.3; Rconti,A; t=0
Exp.3; Rdisco,A; t=0
Exp.3; Rdisco,B; t=0
Exp.3; Rconti,A; t=58 d
Exp.3; Rdisco,A; t=58 d
Exp.3; Rdisco,B; t=58 d

 1
 1
 1
 1
 1
 1
 1
 1
 1
 1
 1
 1
 1
 1
 1
 1
 1
 1
 1
 1
 1

 8.78260237430665E-03
 4.51934530507686E-03
 9.64812272019842E-03
 3.38048887859158E-03
 6.72548306586358E-03
 1.14476367445154E-02
 2.48508717308506E-03
 1.35312917541839E-02
 2.82364847403822E-03
 2.96290767084214E-03
 1.28672098938792E-02
 7.87897952066245E-04
 3.57348912907287E-03
 5.78426897665105E-04
 2.91779621416665E-03
 9.42274085056586E-04
 1.29233067651856E-03
 1.67376351120706E-03
 8.49850663442349E-03
 5.86705127335372E-04
 7.33509603683161E-04

 8.78260237430665E-03
 4.51934530507686E-03
 9.64812272019842E-03
 3.38048887859158E-03
 6.72548306586358E-03
 1.14476367445154E-02
 2.48508717308506E-03
 1.35312917541839E-02
 2.82364847403822E-03
 2.96290767084214E-03
 1.28672098938792E-02
 7.87897952066245E-04
 3.57348912907287E-03
 5.78426897665105E-04
 2.91779621416665E-03
 9.42274085056586E-04
 1.29233067651856E-03
 1.67376351120706E-03
 8.49850663442349E-03
 5.86705127335372E-04
 7.33509603683161E-04

 8.78260237430665E-03
 4.51934530507686E-03
 9.64812272019842E-03
 3.38048887859158E-03
 6.72548306586358E-03
 1.14476367445154E-02
 2.48508717308506E-03
 1.35312917541839E-02
 2.82364847403822E-03
 2.96290767084214E-03
 1.28672098938792E-02
 7.87897952066245E-04
 3.57348912907287E-03
 5.78426897665105E-04
 2.91779621416665E-03
 9.42274085056586E-04
 1.29233067651856E-03
 1.67376351120706E-03
 8.49850663442349E-03
 5.86705127335372E-04
 7.33509603683161E-04

 8.78260237430665E-03
 4.51934530507686E-03
 9.64812272019842E-03
 3.38048887859158E-03
 6.72548306586358E-03
 1.14476367445154E-02
 2.48508717308506E-03
 1.35312917541839E-02
 2.82364847403822E-03
 2.96290767084214E-03
 1.28672098938792E-02
 7.87897952066245E-04
 3.57348912907287E-03
 5.78426897665105E-04
 2.91779621416665E-03
 9.42274085056586E-04
 1.29233067651856E-03
 1.67376351120706E-03
 8.49850663442349E-03
 5.86705127335372E-04
 7.33509603683161E-04

 8.78260237430665E-03
 4.51934530507686E-03
 9.64812272019842E-03
 3.38048887859158E-03
 6.72548306586358E-03
 1.14476367445154E-02
 2.48508717308506E-03
 1.35312917541839E-02
 2.82364847403822E-03
 2.96290767084214E-03
 1.28672098938792E-02
 7.87897952066245E-04
 3.57348912907287E-03
 5.78426897665105E-04
 2.91779621416665E-03
 9.42274085056586E-04
 1.29233067651856E-03
 1.67376351120706E-03
 8.49850663442349E-03
 5.86705127335372E-04
 7.33509603683161E-04

 0
 0
 0
 5.47872335495993E-04
 3.76306790590652E-04
 0
 0
 0
 0
 0
 0
 1.54296682279593E-04
 0
 2.21656046417919E-04
 0
 2.21434409988298E-04
 1.41725597525024E-03
 2.53764145247404E-04
 7.77100801203556E-05
 0
 0

 0
 0
 0
 5.47872335495993E-04
 3.76306790590652E-04
 0
 0
 0
 0
 0
 0
 1.54296682279593E-04
 0
 2.21656046417919E-04
 0
 2.21434409988298E-04
 1.41725597525024E-03
 2.53764145247404E-04
 7.77100801203556E-05
 0
 0

 0
 0
 0
 5.47872335495993E-04
 3.76306790590652E-04
 0
 0
 0
 0
 0
 0

 0
 0
 0
 5.47872335495993E-04
 3.76306790590652E-04
 0
 0
 0
 0
 0
 0

 0
 0
 0
 5.47872335495993E-04
 3.76306790590652E-04
 0
 0
 0
 0
 0
 0

 0
 0
 0
 0
 0
 0
 0
 0
 0
 0
 0
 1.54296682279593E-04
 0
 2.21656046417919E-04
 0
 2.21434409988298E-04
 1.41725597525024E-03
 2.53764145247404E-04
 7.77100801203556E-05
 0
 0

 0
 0
 0
 0
 0
 0
 0
 0
 0
 0
 0
 1.54296682279593E-04
 0
 2.21656046417919E-04
 0
 2.21434409988298E-04
 1.41725597525024E-03
 2.53764145247404E-04
 7.77100801203556E-05
 0
 0

 0
 0
 0
 0
 0
 0
 0
 0
 0
 0
 0
 1.54296682279593E-04
 0
 2.21656046417919E-04
 0
 2.21434409988298E-04
 1.41725597525024E-03
 2.53764145247404E-04
 7.77100801203556E-05
 0
 0

 2.04730995276622E-03
 1.27901017789617E-03
 1.28056517932992E-03
 6.95439140146321E-04
 0
 1.84028946982426E-03
 4.64913645809627E-04
 7.5058119798369E-05
 8.24659167882305E-04
 1.22154965377073E-04
 1.99902895533344E-04
 3.13269021598346E-04
 6.59293496584122E-02
 2.52972980984588E-04
 5.89189072118245E-02
 5.75154311657007E-04
 1.81035387562046E-03
 2.52445889947704E-03
 5.91403986369529E-04
 2.2561478987609E-03
 6.92798065871079E-04

 1.21191717930605E-03
 1.15608817017691E-03
 8.08209171152811E-04
 3.35897919976899E-04
 0
 1.5934826225803E-03
 4.64913645809627E-04
 7.5058119798369E-05
 6.98985419511097E-04
 0
 4.20848201123516E-05
 3.13269021598346E-04
 6.58510129097804E-02
 2.52972980984588E-04
 5.87641748646117E-02
 5.75154311657007E-04
 1.20295845765441E-03
 2.14381268160556E-03
 5.13693906249174E-04
 1.94975744337416E-03
 6.02074509625864E-04

 9.82815027601755E-04
 9.02492316414205E-04
 4.34526630899313E-04
 1.30445794165713E-04
 0
 1.34621916678666E-03
 1.56148525581851E-04
 5.2214344207561E-05
 0
 0
 0
 2.10404566745284E-04
 6.52559359147676E-02
 1.05202283372642E-04
 5.82646596937538E-02
 1.00652004540135E-04
 4.60148043912794E-05
 6.57479830869103E-04
 4.02679506077556E-04
 1.94975744337416E-03
 3.29903840890742E-04

 9.82815027601755E-04
 7.15182590366204E-04
 3.22060914666435E-04
 0
 0
 1.34621916678666E-03
 1.56148525581851E-04
 0
 0
 0
 0
 2.10404566745284E-04
 6.50052583191455E-02
 1.05202283372642E-04
 5.80842904326585E-02
 1.00652004540135E-04
 4.60148043912794E-05
 4.03715685621699E-04
 2.47259345837292E-04
 1.94975744337416E-03
 3.29903840890742E-04

 8.93468206910687E-05
 2.68193471387096E-04
 1.07353638222293E-04
 0
 0
 1.34621916678666E-03
 7.80742627909257E-05
 0
 0
 0
 0
 2.10404566745284E-04
 6.31251763519804E-02
 1.05202283372642E-04
 5.70391912918968E-02

 8.93468206910687E-04
 4.46989118979108E-04
 2.14707276444142E-04
 0
 0
 0
 7.80742627909257E-05
 0
 0
 0
 0
 0
 1.88008196716505E-03
 0
 1.0450991407617E-03
 1.00652004540135E-04
 4.60148043912794E-05
 4.03715685621699E-04
 2.47259345837292E-04
 1.94975744337416E-03
 3.29903840890742E-04

 0
 1.87309726048001E-04
 1.12465716232878E-04
 1.30445794165713E-04
 0
 0
 0
 5.2214344207561E-05
 0
 0
 0
 0
 2.50677595622146E-04
 0
 1.80369261095302E-04

 0
 1.87309726048001E-04
 1.12465716232878E-04
 1.30445794165713E-04
 0
 0
 0
 5.2214344207561E-05
 0
 0
 0
 0
 0
 0
 5.50304632842289E-05

 0
 0
 0
 0
 0
 0
 0
 0
 0
 0
 0
 0
 2.50677595622146E-04
 0
 1.25338797811073E-04

 0
 0
 0
 0
 0
 0
 0
 0
 0
 0
 0
 0
 0
 0
 0
 0
 0
 2.53764145247404E-04
 1.55420160240264E-04
 0
 0

 0
 0
 0
 0
 0
 0
 0
 0
 0
 0
 0
 0
 0
 0
 0
 0
 0
 2.53764145247404E-04
 1.55420160240264E-04
 0
 0

 1.2285187845005E-04
 4.09740025730849E-05
 2.46018754259421E-04
 1.14140069894999E-04
 0
 2.57090465879662E-05
 7.15680742248675E-05
 2.28437755908079E-05
 0
 0
 0
 0
 3.76016393433218E-04
 0
 1.88008196716609E-04
 0
 0
 1.26882072624067E-04
 0
 0
 2.72170668735123E-04

 1.2285187845005E-04
 4.09740025730849E-05
 2.46018754259421E-04
 1.14140069894999E-04
 0
 2.57090465879662E-05
 7.15680742248675E-05
 2.28437755908079E-05
 0
 0
 0
 0
 3.76016393433218E-04
 0
 1.88008196716609E-04

 1.2285187845005E-04
 4.09740025730849E-05
 2.46018754259421E-04
 1.14140069894999E-04
 0
 2.57090465879662E-05
 7.15680742248675E-05
 2.28437755908079E-05
 0
 0
 0

 0
 0
 0
 0
 0
 0
 0
 0
 0
 0
 0
 0
 3.76016393433218E-04
 0
 1.88008196716609E-04

 0
 0
 0
 0
 0
 0
 0
 0
 0
 0
 0
 0
 0
 0
 0
 0
 0
 1.26882072624067E-04
 0
 0
 2.72170668735123E-04

 0
 0
 0
 0
 0
 0
 0
 0
 0
 0
 0
 0
 0
 0
 0
 0
 0
 1.26882072624067E-04
 0
 0
 2.72170668735123E-04

 0
 0
 0
 9.13120559161875E-05
 0
 8.81453025873127E-05
 2.37197046002908E-04
 0
 6.98985419511097E-04
 0
 4.20848201123516E-05
 1.02864454853062E-04
 8.35591985405624E-05
 1.47770697611946E-04
 1.18822247868202E-04
 4.74502307116872E-04
 1.15694365326313E-03
 1.3594507781124E-03
 1.11014400171617E-04
 0
 0

 0
 0
 0
 9.13120559161875E-05
 0
 8.81453025873127E-05
 2.37197046002908E-04
 0
 6.98985419511097E-04
 0
 4.20848201123516E-05
 1.02864454853062E-04
 8.35591985405624E-05
 1.47770697611946E-04
 1.18822247868202E-04
 4.74502307116872E-04
 1.15694365326313E-03
 1.3594507781124E-03
 1.11014400171617E-04
 0
 0

 0
 0
 0
 0
 0
 8.81453025873127E-05
 1.22688127242883E-04
 0
 0
 0
 0

 0
 0
 0
 9.13120559161875E-05
 0
 0
 1.14508918760024E-04
 0
 2.68103996524691E-04
 0
 4.20848201123516E-05
 1.02864454853062E-04
 8.35591985405624E-05
 1.47770697611946E-04
 1.18822247868202E-04

 0
 0
 0
 0
 0
 0
 0
 0
 4.30881422986406E-04
 0
 0

 0
 0
 0
 0
 0
 0
 0
 0
 0
 0
 0
 0
 0
 0
 0
 4.74502307116872E-04
 1.15694365326313E-03
 1.3594507781124E-03
 1.11014400171617E-04
 0
 0

 1.06250273254244E-04
 2.12621851189623E-04
 1.27663785994078E-04
 0
 0
 1.3340910661836E-04
 0
 0
 0
 0
 0
 0
 1.35501403038998E-04
 0
 1.92684726273112E-04

 1.06250273254244E-04
 2.12621851189623E-04
 1.27663785994078E-04
 0
 0
 1.3340910661836E-04
 0
 0
 0
 0
 0
 0
 1.35501403038998E-04
 0
 1.92684726273112E-04

 1.06250273254244E-04
 2.12621851189623E-04
 1.27663785994078E-04
 0
 0
 1.3340910661836E-04
 0
 0
 0
 0
 0

 0
 0
 0
 0
 0
 0
 0
 0
 0
 0
 0
 0
 1.35501403038998E-04
 0
 1.92684726273112E-04

 5.89689016560241E-04
 0
 4.72356008177112E-04
 2.73936167747997E-04
 0
 2.46806847243966E-04
 0
 0
 0
 1.22154965377073E-04
 0
 0
 0
 0
 1.15563972896881E-04
 0
 6.07395417966052E-04
 3.80646217871471E-04
 7.77100801203556E-05
 3.06390455386739E-04
 9.07235562452148E-05

 5.89689016560241E-04
 0
 4.72356008177112E-04
 2.73936167747997E-04
 0
 2.46806847243966E-04
 0
 0
 0
 1.22154965377073E-04
 0
 0
 0
 0
 1.15563972896881E-04
 0
 6.07395417966052E-04
 3.80646217871471E-04
 7.77100801203556E-05
 3.06390455386739E-04
 9.07235562452148E-05

 5.89689016560241E-04
 0
 4.72356008177112E-04
 2.73936167747997E-04
 0
 2.46806847243966E-04
 0
 0
 0
 1.22154965377073E-04
 0
 0
 0
 0
 1.15563972896881E-04
 0
 6.07395417966052E-04
 3.80646217871471E-04
 7.77100801203556E-05
 3.06390455386739E-04
 9.07235562452148E-05

 5.89689016560241E-04
 0
 4.72356008177112E-04
 2.73936167747997E-04
 0
 2.46806847243966E-04
 0
 0
 0
 1.22154965377073E-04
 0
 0
 0
 0
 1.15563972896881E-04
 0
 6.07395417966052E-04
 3.80646217871471E-04
 7.77100801203556E-05
 3.06390455386739E-04
 9.07235562452148E-05

 2.45703756899931E-04
 1.22922007719255E-04
 0
 8.56050524214258E-05
 0
 0
 0
 0
 1.25673748371208E-04
 0
 1.57818075420992E-04
 0
 7.83367486317772E-05
 0
 3.91683743158886E-05

 2.45703756899931E-04
 1.22922007719255E-04
 0
 8.56050524214258E-05
 0
 0
 0
 0
 1.25673748371208E-04
 0
 1.57818075420992E-04
 0
 7.83367486317772E-05
 0
 3.91683743158886E-05

 2.45703756899931E-04
 1.22922007719255E-04
 0
 8.56050524214258E-05
 0
 0
 0
 0
 1.25673748371208E-04
 0
 1.57818075420992E-04
 0
 7.83367486317772E-05
 0
 3.91683743158886E-05

 2.45703756899931E-04
 1.22922007719255E-04
 0
 8.56050524214258E-05
 0
 0
 0
 0
 1.25673748371208E-04
 0
 1.57818075420992E-04
 0
 7.83367486317772E-05
 0
 3.91683743158886E-05

 0
 0
 0
 5.82842910103324E-05
 0
 1.05024190316581E-04
 0
 0
 0
 0
 0

 0
 0
 0
 5.82842910103324E-05
 0
 1.05024190316581E-04
 0
 0
 0
 0
 0

 0
 0
 0
 5.82842910103324E-05
 0
 1.05024190316581E-04
 0
 0
 0
 0
 0

 0
 0
 0
 5.82842910103324E-05
 0
 1.05024190316581E-04
 0
 0
 0
 0
 0

 0
 0
 0
 5.82842910103324E-05
 0
 1.05024190316581E-04
 0
 0
 0
 0
 0

 9.82815027601755E-04
 1.96675212350808E-04
 0
 1.36968083874281E-04
 0
 0
 0
 1.09650122835878E-04
 0
 1.22154965377073E-04
 3.78763381010121E-04
 8.02342747854221E-03
 5.01355191244291E-03
 5.89031390788749E-03
 3.43128773939608E-03
 6.6430322996362E-04
 6.07395417966052E-04
 6.34410363119604E-04
 4.66260480723027E-04
 0
 9.07235562452148E-05

 9.82815027601755E-04
 1.96675212350808E-04
 0
 1.36968083874281E-04
 0
 0
 0
 1.09650122835878E-04
 0
 1.22154965377073E-04
 3.78763381010121E-04
 8.02342747854221E-03
 5.01355191244291E-03
 5.89031390788749E-03
 3.43128773939608E-03
 6.6430322996362E-04
 6.07395417966052E-04
 6.34410363119604E-04
 4.66260480723027E-04
 0
 9.07235562452148E-05

 9.82815027601755E-04
 1.96675212350808E-04
 0
 1.36968083874281E-04
 0
 0
 0
 1.09650122835878E-04
 0
 1.22154965377073E-04
 3.78763381010121E-04
 8.02342747854221E-03
 5.01355191244291E-03
 5.89031390788749E-03
 3.43128773939608E-03
 6.6430322996362E-04
 6.07395417966052E-04
 6.34410363119604E-04
 4.66260480723027E-04
 0
 9.07235562452148E-05

 9.82815027601755E-04
 1.96675212350808E-04
 0
 1.36968083874281E-04
 0
 0
 0
 1.09650122835878E-04
 0
 1.22154965377073E-04
 3.78763381010121E-04
 8.02342747854221E-03
 5.01355191244291E-03
 5.89031390788749E-03
 3.43128773939608E-03
 6.6430322996362E-04
 6.07395417966052E-04
 6.34410363119604E-04
 4.66260480723027E-04
 0
 9.07235562452148E-05

 9.82815027601755E-04
 1.96675212350808E-04
 0
 1.36968083874281E-04
 0
 0
 0
 1.09650122835878E-04
 0
 1.22154965377073E-04
 3.78763381010121E-04
 8.02342747854221E-03
 5.01355191244291E-03
 5.89031390788749E-03
 3.43128773939608E-03
 6.6430322996362E-04
 6.07395417966052E-04
 6.34410363119604E-04
 4.66260480723027E-04
 0
 9.07235562452148E-05

 5.01862992816536E-04
 5.8584105806347E-04
 5.02506391678817E-04
 9.32548656164356E-04
 1.60130549187181E-04
 5.2512095158399E-05
 8.77089590499899E-04
 2.33298133693358E-04
 1.62573700020415E-03
 1.55942508991793E-04
 2.68626511355436E-04
 1.31316325344574E-04
 3.73349610500092E-04
 1.88643443760276E-04
 4.32555598647215E-04
 6.59591859538527E-04
 5.16932270607672E-04
 7.01900827282382E-04
 6.28294264801164E-04
 3.25947292965178E-04
 2.31634611689688E-04

 5.01862992816536E-04
 5.8584105806347E-04
 5.02506391678817E-04
 9.32548656164356E-04
 1.60130549187181E-04
 5.2512095158399E-05
 8.77089590499899E-04
 2.33298133693358E-04
 1.62573700020415E-03
 1.55942508991793E-04
 2.68626511355436E-04
 1.31316325344574E-04
 2.66678293214073E-04
 1.88643443760276E-04
 3.79219940004205E-04
 4.71137042527751E-04
 4.73854581390304E-04
 7.01900827282382E-04
 5.290899072009E-04
 2.60757834372068E-04
 1.93028843074703E-04

 5.01862992816536E-04
 5.8584105806347E-04
 5.02506391678817E-04
 9.32548656164356E-04
 1.60130549187181E-04
 5.2512095158399E-05
 8.77089590499899E-04
 2.33298133693358E-04
 1.62573700020415E-03
 1.55942508991793E-04
 2.68626511355436E-04
 1.31316325344574E-04
 2.66678293214073E-04
 1.88643443760276E-04
 3.79219940004205E-04
 4.71137042527751E-04
 4.73854581390304E-04
 7.01900827282382E-04
 5.290899072009E-04
 2.60757834372068E-04
 1.93028843074703E-04

 5.01862992816536E-04
 5.8584105806347E-04
 5.02506391678817E-04
 9.32548656164356E-04
 1.60130549187181E-04
 5.2512095158399E-05
 8.77089590499899E-04
 2.33298133693358E-04
 1.62573700020415E-03
 1.55942508991793E-04
 2.68626511355436E-04
 1.31316325344574E-04
 2.66678293214073E-04
 1.88643443760276E-04
 3.79219940004205E-04
 4.71137042527751E-04
 4.73854581390304E-04
 7.01900827282382E-04
 5.290899072009E-04
 2.60757834372068E-04
 1.93028843074703E-04

 5.01862992816536E-04
 5.8584105806347E-04
 5.02506391678817E-04
 9.32548656164356E-04
 1.60130549187181E-04
 5.2512095158399E-05
 8.77089590499899E-04
 2.33298133693358E-04
 1.62573700020415E-03
 1.55942508991793E-04
 2.68626511355436E-04
 1.31316325344574E-04
 2.66678293214073E-04
 1.88643443760276E-04
 3.79219940004205E-04
 4.71137042527751E-04
 4.73854581390304E-04
 7.01900827282382E-04
 5.290899072009E-04
 2.60757834372068E-04
 1.93028843074703E-04

 0
 0
 0
 0
 0
 0
 0
 0
 0
 0
 0
 0
 1.06671317286019E-04
 0
 5.33356586430097E-05
 1.88454817010775E-04
 4.30776892173679E-05
 0
 9.92043576002638E-05
 6.51894585931107E-05
 3.8605768614985E-05

 0
 0
 0
 0
 0
 0
 0
 0
 0
 0
 0
 0
 1.06671317286019E-04
 0
 5.33356586430097E-05
 1.88454817010775E-04
 4.30776892173679E-05
 0
 9.92043576002638E-05
 6.51894585931107E-05
 3.8605768614985E-05

 0
 0
 0
 0
 0
 0
 0
 0
 0
 0
 0
 0
 1.06671317286019E-04
 0
 5.33356586430097E-05
 1.88454817010775E-04
 4.30776892173679E-05
 0
 9.92043576002638E-05
 6.51894585931107E-05
 3.8605768614985E-05

 0
 0
 0
 0
 0
 0
 0
 0
 0
 0
 0
 0
 1.06671317286019E-04
 0
 5.33356586430097E-05
 1.88454817010775E-04
 4.30776892173679E-05
 0
 9.92043576002638E-05
 6.51894585931107E-05
 3.8605768614985E-05

 .320618331525728
 .216807972937898
 .219116772785214
 .135795733319011
 6.52178718525009E-02
 .402150799995394
 .126798092562107
 .102998898170617
 .110493532460091
 5.08445104740419E-02
 .076807106940985
 4.15975547093424E-02
 6.69129539147651E-02
 3.72858184543292E-02
 5.91904323029242E-02
 7.72582652350799E-02
 7.14008754862465E-02
 6.38728762617839E-02
 7.53563795145119E-02
 8.24748818223367E-02
 .218996333280662

 0
 5.31554627975156E-04
 1.27663785994078E-04
 7.40368020942061E-05
 0
 1.3340910661836E-04
 1.85690138529386E-04
 1.77811010003882E-04
 0
 0
 0

 0
 5.31554627975156E-04
 1.27663785994078E-04
 7.40368020942061E-05
 0
 1.3340910661836E-04
 1.85690138529386E-04
 1.77811010003882E-04
 0
 0
 0

 0
 5.31554627975156E-04
 1.27663785994078E-04
 7.40368020942061E-05
 0
 1.3340910661836E-04
 1.85690138529386E-04
 1.77811010003882E-04
 0
 0
 0

 0
 5.31554627975156E-04
 1.27663785994078E-04
 7.40368020942061E-05
 0
 1.3340910661836E-04
 1.85690138529386E-04
 1.77811010003882E-04
 0
 0
 0

 .295214233933495
 .177087297748945
 .19632577754124
 9.63472635708912E-02
 4.02155483229361E-02
 .39612498982648
 8.39922919099699E-02
 6.68186962823183E-02
 .102846608095395
 3.16788543543977E-02
 5.09662201853855E-02
 1.08485262564678E-02
 .024608183970214
 1.00760349743287E-02
 .022506739878004
 4.19407317013149E-02
 4.36047242317172E-02
 4.24058012719077E-02
 2.22858044271951E-02
 6.26568481267058E-02
 .171100306908825

 .295017670927975
 .176890622536594
 .19632577754124
 9.60733274031432E-02
 4.02155483229361E-02
 .39612498982648
 8.39922919099699E-02
 .066599396036647
 .102846608095395
 3.16788543543977E-02
 5.09662201853855E-02
 9.92274616279142E-03
 2.43575063745919E-02
 9.46863722221239E-03
 .022265837107296
 .039726387601432
 .042693631104774
 4.12638626182962E-02
 2.15864137061151E-02
 6.26568481267058E-02
 .169920900677636

 4.68007156000836E-04
 1.03020349326459E-03
 1.12465716232878E-03
 1.10878925040869E-03
 2.8670993568745E-03
 0
 0
 1.00773684320437E-02
 9.57514273304444E-05
 2.85028252546599E-03
 5.86181422992475E-03
 3.5488236924343E-03
 1.37872677591951E-03
 3.50850430955515E-03
 2.07613106272274E-03
 2.76793012485054E-02
 2.93574452015955E-02
 2.95635229213426E-02
 4.19634432649384E-03
 6.12780910774359E-04
 5.44341337472332E-04

 4.68007156000836E-04
 1.03020349326459E-03
 1.12465716232878E-03
 1.10878925040869E-03
 2.8670993568745E-03
 0
 0
 1.00773684320437E-02
 9.57514273304444E-05
 2.85028252546599E-03
 5.86181422992475E-03
 3.5488236924343E-03
 1.37872677591951E-03
 3.50850430955515E-03
 2.07613106272274E-03
 2.76793012485054E-02
 2.93574452015955E-02
 2.95635229213426E-02
 4.19634432649384E-03
 6.12780910774359E-04
 5.44341337472332E-04

 0
 0
 0
 0
 0
 0
 0
 5.2214344207561E-05
 0
 0
 0
 0
 0
 0
 0
 2.10889914273963E-04
 4.82059855527689E-05
 1.81260103748319E-04
 2.22028800344299E-04
 0
 0

 0
 0
 0
 0
 0
 0
 0
 5.2214344207561E-05
 0
 0
 0
 0
 0
 0
 0
 2.10889914273963E-04
 4.82059855527689E-05
 1.81260103748319E-04
 2.22028800344299E-04
 0
 0

 9.82815027601755E-05
 9.83376061754038E-05
 3.54267006133078E-04
 0
 9.4076697647663E-05
 1.23403423621983E-04
 0
 0
 0
 0
 3.15636150842637E-05
 0
 0
 0
 5.50304632842289E-05
 2.10889914273963E-04
 4.82059855527689E-04
 5.43780311243568E-04
 0
 0
 8.64033868999561E-05

 9.82815027601755E-05
 9.83376061754038E-05
 3.54267006133078E-04
 0
 9.4076697647663E-05
 1.23403423621983E-04
 0
 0
 0
 0
 3.15636150842637E-05
 0
 0
 0
 5.50304632842289E-05
 2.10889914273963E-04
 4.82059855527689E-04
 5.43780311243568E-04
 0
 0
 8.64033868999561E-05

 7.66595721527827E-03
 5.8346979663943E-03
 4.72356008177112E-03
 2.04539005251798E-02
 9.4076697647663E-03
 9.87227388973824E-04
 .010477566066511
 1.53510171969958E-02
 3.53897275412517E-02
 8.18438268022957E-03
 6.75461362802513E-03
 2.08300521077408E-03
 3.5094863387066E-03
 1.76404113177633E-03
 3.14151084411629E-03
 2.76793012485436E-03
 .003897453931937
 2.85484663403749E-03
 2.91412800451088E-03
 1.83834273232484E-03
 2.72170668735644E-03

 0
 1.31116808233872E-04
 0
 1.82624111831998E-04
 0
 0
 0
 0
 0
 0
 0

 7.27283120423837E-03
 5.70358115816042E-03
 4.72356008177112E-03
 1.98603721617255E-02
 9.4076697647663E-03
 8.63823965351587E-04
 .010477566066511
 1.53510171969958E-02
 3.49875715464646E-02
 8.18438268022957E-03
 6.75461362802513E-03
 3.85741705698276E-04
 6.26693989054218E-04
 1.92870852849138E-04
 5.44474940321293E-04
 1.21788925493373E-03
 9.61709411775702E-04
 3.17205181559802E-04
 1.94275200300666E-04
 0
 0

 3.9312601103989E-04
 0
 0
 4.10904251622278E-04
 0
 1.23403423622238E-04
 0
 0
 4.02155994787036E-04
 0
 0
 1.6972635050758E-03
 2.88279234965238E-03
 1.57117027892719E-03
 .002597035903795
 1.32860645993233E-03
 2.83451195050048E-03
 2.28387730723028E-03
 2.71985280421021E-03
 1.5319522769381E-03
 2.17736534988411E-03

 0
 0
 0
 0
 0
 0
 0
 0
 0
 0
 0
 0
 0
 0
 0
 2.21434409988298E-04
 1.01232569660815E-04
 2.53764145247404E-04
 0
 3.06390455386739E-04
 5.44341337472332E-04

 .286785425053936
 .16992738347076
 .190123293291007
 7.45106376275547E-02
 2.78467025036476E-02
 .395014359013884
 7.35147258434589E-02
 .0411187960634
 6.73611291268131E-02
 2.06441891487021E-02
 3.83182287123514E-02
 2.16015355191261E-03
 1.50406557373058E-03
 2.23613841818075E-03
 .001792108542937
 8.85737639952427E-03
 8.90846613016101E-03
 8.12045264792422E-03
 .014253912574766
 6.02057244836066E-02
 .166568449265908

 .285606047020817
 .167567280922557
 .188233869258297
 .071497339782325
 2.70940889224679E-02
 .393410114506801
 7.14535653057841E-02
 3.87064933610143E-02
 6.43449591659091E-02
 1.96669494256865E-02
 3.59193939659505E-02
 0
 0
 0
 0
 0
 0
 0
 1.88388073018773E-04
 0
 0

 0
 0
 0
 0
 0
 7.40420541731388E-04
 0
 0
 2.01077997393933E-04
 0
 0
 2.16015355191261E-03
 1.50406557373058E-03
 2.23613841818075E-03
 1.56098059714282E-03
 1.10717204994022E-03
 2.53081424151746E-03
 2.03011316197923E-03
 1.26667430595827E-02
 6.02057244836066E-02
 .166568449265908

 1.17937803311886E-03
 2.36010254820319E-03
 1.8894240327104E-03
 3.01329784522966E-03
 7.52613581179749E-04
 8.63823965351587E-04
 2.06116053767476E-03
 2.4123027023857E-03
 2.81509196351008E-03
 9.77239723015573E-04
 2.39883474640091E-03
 0
 0
 0
 2.31127945794184E-04
 7.75020434958406E-03
 6.37765188864355E-03
 6.09033948594499E-03
 1.39878144216461E-03
 0
 0

 0
 0
 0
 0
 0
 0
 0
 0
 0
 0
 0
 2.13076370767043E-03
 1.79652276862352E-02
 1.95995336270016E-03
 1.52010561942358E-02

 0
 0
 0
 0
 0
 0
 0
 0
 0
 0
 0
 2.13076370767043E-03
 1.79652276862352E-02
 1.95995336270016E-03
 1.52010561942358E-02

 1.96563005520351E-04
 1.96675212350808E-04
 0
 2.73936167747997E-04
 0
 0
 0
 2.19300245671303E-04
 0
 0
 0
 9.25780093676428E-04
 2.50677595622146E-04
 6.07397752116336E-04
 2.40902770707953E-04
 2.21434409988298E-03
 9.11093126943257E-04
 1.14193865361149E-03
 6.99390721080072E-04
 0
 1.17940623118831E-03

 1.96563005520351E-04
 1.96675212350808E-04
 0
 2.73936167747997E-04
 0
 0
 0
 2.19300245671303E-04
 0
 0
 0
 9.25780093676428E-04
 2.50677595622146E-04
 6.07397752116336E-04
 2.40902770707953E-04
 2.21434409988298E-03
 9.11093126943257E-04
 1.14193865361149E-03
 6.99390721080072E-04
 0
 1.17940623118831E-03

 1.96563005520351E-04
 1.96675212350808E-04
 0
 2.73936167747997E-04
 0
 0
 0
 2.19300245671303E-04
 0
 0
 0
 9.25780093676428E-04
 2.50677595622146E-04
 6.07397752116336E-04
 2.40902770707953E-04

 0
 0
 0
 0
 0
 0
 0
 0
 0
 0
 0
 0
 0
 0
 0
 2.21434409988298E-03
 9.11093126943257E-04
 1.14193865361149E-03
 6.99390721080072E-04
 0
 1.17940623118831E-03

 0
 4.03436333026464E-04
 3.63350775521106E-04
 3.51200215062259E-04
 1.92977841328141E-04
 6.3283806985763E-05
 8.80837836615572E-05
 1.68692496670349E-04
 0
 0
 0
 0
 7.12395731147102E-04
 0
 7.21656583282303E-04
 0
 0
 0
 2.98884923539829E-05
 5.89212414206962E-05
 0

 0
 4.03436333026464E-04
 3.63350775521106E-04
 3.51200215062259E-04
 1.92977841328141E-04
 6.3283806985763E-05
 8.80837836615572E-05
 1.68692496670349E-04
 0
 0
 0
 0
 7.12395731147102E-04
 0
 7.21656583282303E-04
 0
 0
 0
 2.98884923539829E-05
 5.89212414206962E-05
 0

 0
 4.03436333026464E-04
 3.63350775521106E-04
 3.51200215062259E-04
 1.92977841328141E-04
 6.3283806985763E-05
 8.80837836615572E-05
 1.68692496670349E-04
 0
 0
 0
 0
 6.42763065696634E-04
 0
 5.58435836226967E-04
 0
 0
 0
 2.98884923539829E-05
 5.89212414206962E-05
 0

 0
 4.03436333026464E-04
 3.63350775521106E-04
 3.51200215062259E-04
 1.92977841328141E-04
 0
 8.80837836615572E-05
 1.68692496670349E-04
 0
 0
 0
 0
 0
 0
 0

 0
 0
 0
 0
 0
 6.3283806985763E-05
 0
 0
 0
 0
 0
 0
 6.42763065696634E-04
 0
 5.58435836226967E-04

 0
 0
 0
 0
 0
 0
 0
 0
 0
 0
 0
 0
 0
 0
 0
 0
 0
 0
 2.98884923539829E-05
 5.89212414206962E-05
 0

 0
 0
 0
 0
 0
 0
 0
 0
 0
 0
 0
 0
 6.96326654504686E-05
 0
 1.63220747055336E-04

 0
 0
 0
 0
 0
 0
 0
 0
 0
 0
 0
 0
 6.96326654504686E-05
 0
 1.63220747055336E-04

 .021685338028347
 3.44269362785661E-02
 1.70657654567083E-02
 3.36185461780989E-02
 2.00292324023656E-02
 .005095367168909
 3.95609845134766E-02
 2.65282555247683E-02
 4.92965412964968E-03
 1.39493089494953E-02
 1.96305322201135E-02
 2.65788478507204E-02
 2.62807156700369E-02
 2.31718863508413E-02
 2.29819348817207E-02
 2.95722083016457E-02
 2.25977220016845E-02
 1.77634901673406E-02
 4.88821471723125E-02
 1.51218515078116E-02
 4.10304599532774E-02

 .021685338028347
 3.44269362785661E-02
 1.70657654567083E-02
 3.36185461780989E-02
 2.00292324023656E-02
 .005095367168909
 3.95609845134766E-02
 2.65282555247683E-02
 4.92965412964968E-03
 1.39493089494953E-02
 1.96305322201135E-02
 2.65788478507204E-02
 2.62807156700369E-02
 2.31718863508413E-02
 2.29819348817207E-02
 2.95722083016457E-02
 2.25977220016845E-02
 1.77634901673406E-02
 4.88821471723125E-02
 1.51218515078116E-02
 4.10304599532774E-02

 0
 1.6738315944715E-04
 0
 0
 0
 0
 0
 0
 0
 0
 0

 0
 1.6738315944715E-04
 0
 0
 0
 0
 0
 0
 0
 0
 0

 3.80444526813059E-04
 0
 0
 0
 0
 5.573057841001E-04
 0
 0
 0
 0
 0
 0
 4.04318702616364E-04
 0
 2.76716753177137E-04
 0
 1.30622670529708E-04
 0
 0
 0
 4.09719286268375E-04

 2.53629684541865E-04
 0
 0
 0
 0
 1.59230224028365E-04
 0
 0
 0
 0
 0
 0
 2.42591221569818E-04
 0
 1.21295610784909E-04
 0
 1.30622670529708E-04
 0
 0
 0
 4.09719286268375E-04

 1.26814842271194E-04
 0
 0
 0
 0
 3.98075560071735E-04
 0
 0
 0
 0
 0
 0
 1.61727481046545E-04
 0
 1.55421142392228E-04

 1.71200037065903E-02
 .031468033976081
 1.41706802452973E-02
 3.08399104980731E-02
 1.74800573693341E-02
 2.38845336042712E-04
 .037123052694718
 2.32741228470654E-02
 2.59455480507765E-04
 1.24519255029428E-02
 1.38065361465097E-02
 0
 0
 0
 0
 .02471493737288
 1.44991164288295E-02
 1.06417222200877E-02
 4.79797075322069E-02
 1.42323308308793E-02
 4.00939587275203E-02

 1.71200037065903E-02
 .031468033976081
 1.41706802452973E-02
 3.08399104980731E-02
 1.74800573693341E-02
 2.38845336042712E-04
 .037123052694718
 2.32741228470654E-02
 2.59455480507765E-04
 1.24519255029428E-02
 1.38065361465097E-02
 0
 0
 0
 0
 .02471493737288
 1.44991164288295E-02
 1.06417222200877E-02
 4.79797075322069E-02
 1.42323308308793E-02
 4.00939587275203E-02

 0
 0
 0
 3.04373519720206E-04
 0
 0
 0
 0
 0
 0
 0
 1.26423733222519E-02
 1.66579305477912E-02
 1.06098024306747E-02
 1.45172296290152E-02

 0
 0
 0
 3.04373519720206E-04
 0
 0
 0
 0
 0
 0
 0

 0
 0
 0
 0
 0
 0
 0
 0
 0
 0
 0
 1.26423733222519E-02
 1.66579305477912E-02
 1.06098024306747E-02
 1.45172296290152E-02

 4.18488979494365E-03
 2.79151914303796E-03
 2.89508521141094E-03
 2.47426216030558E-03
 2.54917503303144E-03
 4.29921604876618E-03
 2.43793181875861E-03
 3.25413267770292E-03
 4.67019864914191E-03
 1.49738344655246E-03
 5.82399607360378E-03
 1.39364745284684E-02
 9.21846641962929E-03
 1.25620839201666E-02
 8.18798849952831E-03
 4.85727092876571E-03
 7.96798290232533E-03
 7.1217679472529E-03
 9.02439640105626E-04
 8.89520676932369E-04
 5.26781939488681E-04

 1.26814842271194E-04
 0
 1.52372905863899E-04
 0
 0
 0
 0
 0
 0
 0
 0
 0
 6.46909924184703E-04
 0
 6.96241971436446E-04
 0
 0
 0
 1.50406606684271E-04
 1.97671261539832E-04
 3.51187959659569E-04

 2.53629684541865E-04
 2.53774467548905E-04
 3.04745811727169E-04
 2.65099517175663E-04
 1.21389287287307E-03
 2.46806847244015E-03
 3.32445248012516E-04
 1.76855036831769E-03
 0
 5.51667585571273E-04
 1.42545358444725E-03
 4.47958109843888E-03
 8.08637405231249E-05
 3.82471376839833E-03
 4.04318702615624E-05
 0
 0
 0
 0
 5.93013784622337E-04
 1.17062653219295E-04

 3.80444526813059E-03
 2.53774467548905E-03
 2.43796649381987E-03
 2.20916264312992E-03
 1.33528216015837E-03
 1.83114757632603E-03
 2.10548657074609E-03
 1.48558230938523E-03
 4.67019864914191E-03
 9.45715860981185E-04
 4.39854248915652E-03
 9.45689343002954E-03
 8.49069275492146E-03
 8.7373701517683E-03
 7.4513146578303E-03
 4.85727092876571E-03
 7.96798290232533E-03
 7.1217679472529E-03
 7.52033033421355E-04
 9.88356307702E-05
 5.8531326609816E-05

 3.71875956388537E-03
 4.35874794938574E-03
 5.23421522574981E-03
 5.4046865528642E-03
 4.78011328587117E-03
 7.33750086401668E-04
 2.97104221646941E-03
 9.30544285685619E-03
 2.71727023504597E-03
 5.21634717014899E-03
 6.21035453548596E-03
 4.17018060215421E-03
 1.53116585433671E-02
 4.03789712915912E-03
 1.29030583113193E-02
 5.74532523211917E-03
 5.19842925284478E-03
 3.70358482253559E-03
 4.15853942265035E-03
 4.63726094639865E-03
 6.86556641855961E-03

 3.71875956388537E-03
 4.35874794938574E-03
 5.23421522574981E-03
 5.4046865528642E-03
 4.78011328587117E-03
 7.33750086401668E-04
 2.97104221646941E-03
 9.30544285685619E-03
 2.71727023504597E-03
 5.21634717014899E-03
 6.21035453548596E-03
 4.17018060215421E-03
 1.53116585433671E-02
 4.03789712915912E-03
 1.29030583113193E-02
 5.74532523211917E-03
 5.19842925284478E-03
 3.70358482253559E-03
 4.15853942265035E-03
 4.63726094639865E-03
 6.86556641855961E-03

 2.23125573833298E-03
 2.33884036308717E-03
 3.0639308638547E-03
 2.36917766700909E-03
 2.64431798792843E-03
 3.33522766546589E-04
 1.11414083117555E-03
 3.55622020007274E-03
 6.5214485641096E-04
 2.17898046347965E-03
 2.69570334232945E-03
 2.75231919742327E-03
 3.72628858356624E-03
 2.70406824180747E-03
 2.86261648980974E-03
 1.79541413503681E-03
 1.75104985359058E-03
 1.3716980824184E-03
 1.59620705111744E-03
 1.24212346778527E-03
 1.81447112490119E-03

 2.23125573833298E-03
 2.33884036308717E-03
 3.0639308638547E-03
 2.36917766700909E-03
 2.64431798792843E-03
 3.33522766546589E-04
 1.11414083117555E-03
 3.55622020007274E-03
 6.5214485641096E-04
 2.17898046347965E-03
 2.69570334232945E-03
 2.75231919742327E-03
 3.72628858356624E-03
 2.70406824180747E-03
 2.86261648980974E-03
 1.79541413503681E-03
 1.75104985359058E-03
 1.3716980824184E-03
 1.59620705111744E-03
 1.24212346778527E-03
 1.81447112490119E-03

 7.43751912776196E-04
 7.44176479161705E-04
 1.02131028795157E-03
 1.70284644816307E-03
 1.72897714595369E-03
 1.3340910661836E-04
 5.57070415587774E-04
 3.73403121007393E-03
 4.34763237607606E-04
 1.78280219739293E-03
 2.21798376266953E-03
 4.17018060214353E-04
 9.07859400359054E-03
 4.42845849477533E-04
 7.66264762062417E-03
 2.87266261606303E-03
 2.6265747803843E-03
 1.64603769890602E-03
 1.47019070497849E-03
 2.15301401082812E-03
 3.67798200994819E-03

 7.43751912776196E-04
 7.44176479161705E-04
 1.02131028795157E-03
 1.70284644816307E-03
 1.72897714595369E-03
 1.3340910661836E-04
 5.57070415587774E-04
 3.73403121007393E-03
 4.34763237607606E-04
 1.78280219739293E-03
 2.21798376266953E-03
 4.17018060214353E-04
 9.07859400359054E-03
 4.42845849477533E-04
 7.66264762062417E-03
 2.87266261606303E-03
 2.6265747803843E-03
 1.64603769890602E-03
 1.47019070497849E-03
 2.15301401082812E-03
 3.67798200994819E-03

 7.43751912776196E-04
 1.27573110713686E-03
 1.14897407394354E-03
 1.33266243769204E-03
 4.06818151989054E-04
 2.6681821323672E-04
 1.29983096970608E-03
 2.01519144670952E-03
 1.6303621410274E-03
 1.25456450927641E-03
 1.29666743048698E-03
 1.00084334451658E-03
 2.5067759562103E-03
 8.90983037874123E-04
 2.37779420088538E-03
 1.07724848101933E-03
 8.20804618869897E-04
 6.85849041211172E-04
 1.09214166655442E-03
 1.24212346778527E-03
 1.37311328371023E-03

 7.43751912776196E-04
 1.27573110713686E-03
 1.14897407394354E-03
 1.33266243769204E-03
 4.06818151989054E-04
 2.6681821323672E-04
 1.29983096970608E-03
 2.01519144670952E-03
 1.6303621410274E-03
 1.25456450927641E-03
 1.29666743048698E-03
 1.00084334451658E-03
 2.5067759562103E-03
 8.90983037874123E-04
 2.37779420088538E-03
 1.07724848101933E-03
 8.20804618869897E-04
 6.85849041211172E-04
 1.09214166655442E-03
 1.24212346778527E-03
 1.37311328371023E-03

 0
 0
 0
 0
 0
 0
 0
 0
 0
 0
 0
 0
 0
 0
 7.70426485979204E-05

 0
 0
 0
 0
 0
 0
 0
 0
 0
 0
 0
 0
 0
 0
 7.70426485979204E-05

 0
 0
 0
 0
 0
 0
 0
 0
 0
 0
 0
 0
 0
 0
 7.70426485979204E-05

 0
 0
 0
 0
 0
 0
 0
 0
 0
 0
 0
 0
 0
 0
 7.70426485979204E-05

 0
 0
 0
 0
 3.76306790590652E-04
 4.93613694487932E-04
 3.43526756280073E-04
 0
 0
 0
 0

 0
 0
 0
 0
 3.76306790590652E-04
 4.93613694487932E-04
 3.43526756280073E-04
 0
 0
 0
 0

 0
 0
 0
 0
 3.76306790590652E-04
 4.93613694487932E-04
 3.43526756280073E-04
 0
 0
 0
 0

 0
 0
 0
 0
 3.76306790590652E-04
 4.93613694487932E-04
 3.43526756280073E-04
 0
 0
 0
 0

 0
 0
 0
 0
 3.76306790590652E-04
 4.93613694487932E-04
 3.43526756280073E-04
 0
 0
 0
 0

 1.00372598563307E-03
 2.00859791336788E-03
 1.60802045337055E-03
 9.90832947173725E-04
 0
 0
 9.50180389710237E-04
 0
 5.13390631642671E-04
 0
 8.05879534065197E-05
 5.45619331805182E-02
 2.54411091727068E-02
 4.56057734722952E-02
 2.01461545469598E-02
 .150104261749206
 7.90044820245235E-02
 .103611360580998
 4.96021788001319E-03
 1.30378917185846E-04
 1.54423074459718E-04

 1.00372598563307E-03
 2.00859791336788E-03
 1.60802045337055E-03
 9.90832947173725E-04
 0
 0
 9.50180389710237E-04
 0
 5.13390631642671E-04
 0
 8.05879534065197E-05
 5.45619331805182E-02
 2.54411091727068E-02
 4.56057734722952E-02
 2.01461545469598E-02
 .150104261749206
 7.90044820245235E-02
 .103611360580998
 4.96021788001319E-03
 1.30378917185846E-04
 1.54423074459718E-04

 1.00372598563307E-03
 2.00859791336788E-03
 1.60802045337055E-03
 9.90832947173725E-04
 0
 0
 9.50180389710237E-04
 0
 5.13390631642671E-04
 0
 8.05879534065197E-05
 5.45619331805182E-02
 2.54411091727068E-02
 4.56057734722952E-02
 2.01461545469598E-02
 .150104261749206
 7.90044820245235E-02
 .103611360580998
 4.96021788001319E-03
 1.30378917185846E-04
 1.54423074459718E-04

 1.00372598563307E-03
 2.00859791336788E-03
 1.60802045337055E-03
 9.90832947173725E-04
 0
 0
 9.50180389710237E-04
 0
 5.13390631642671E-04
 0
 8.05879534065197E-05
 5.45619331805182E-02
 2.54411091727068E-02
 4.56057734722952E-02
 2.01461545469598E-02
 .150104261749206
 7.90044820245235E-02
 .103611360580998
 4.96021788001319E-03
 1.30378917185846E-04
 1.54423074459718E-04

 1.00372598563307E-03
 2.00859791336788E-03
 1.60802045337055E-03
 9.90832947173725E-04
 0
 0
 9.50180389710237E-04
 0
 5.13390631642671E-04
 0
 8.05879534065197E-05
 5.45619331805182E-02
 2.54411091727068E-02
 4.56057734722952E-02
 2.01461545469598E-02
 .150104261749206
 7.90044820245235E-02
 .103611360580998
 4.96021788001319E-03
 1.30378917185846E-04
 1.54423074459718E-04

 8.36438321363196E-05
 0
 0
 0
 0
 0
 0
 0
 8.55651052740141E-05
 5.19808363306693E-05
 2.68626511355436E-05
 0
 8.53370538286204E-04
 0
 1.16432764933551E-03
 1.31918371907814E-03
 1.16309760886621E-03
 2.6996185664664E-03
 3.30681192001513E-05
 6.51894585931107E-05
 0

 8.36438321363196E-05
 0
 0
 0
 0
 0
 0
 0
 8.55651052740141E-05
 5.19808363306693E-05
 2.68626511355436E-05
 0
 8.53370538286204E-04
 0
 1.16432764933551E-03
 1.31918371907814E-03
 1.16309760886621E-03
 2.6996185664664E-03
 3.30681192001513E-05
 6.51894585931107E-05
 0

 8.36438321363196E-05
 0
 0
 0
 0
 0
 0
 0
 8.55651052740141E-05
 5.19808363306693E-05
 2.68626511355436E-05
 0
 8.53370538286204E-04
 0
 1.16432764933551E-03
 1.31918371907814E-03
 1.16309760886621E-03
 2.6996185664664E-03
 3.30681192001513E-05
 6.51894585931107E-05
 0

 8.36438321363196E-05
 0
 0
 0
 0
 0
 0
 0
 8.55651052740141E-05
 5.19808363306693E-05
 2.68626511355436E-05
 0
 8.53370538286204E-04
 0
 1.16432764933551E-03
 1.31918371907814E-03
 1.16309760886621E-03
 2.6996185664664E-03
 3.30681192001513E-05
 6.51894585931107E-05
 0

 8.36438321363196E-05
 0
 0
 0
 0
 0
 0
 0
 8.55651052740141E-05
 5.19808363306693E-05
 2.68626511355436E-05
 0
 8.53370538286204E-04
 0
 1.16432764933551E-03
 1.31918371907814E-03
 1.16309760886621E-03
 2.6996185664664E-03
 3.30681192001513E-05
 6.51894585931107E-05
 0

 2.18403339467057E-04
 0
 0
 1.52186759860312E-04
 0
 0
 0
 1.21833469817642E-04
 0
 0
 0
 0
 1.39265330900937E-04
 1.60564116975691E-04
 5.83250322770877E-04

 2.18403339467057E-04
 0
 0
 1.52186759860312E-04
 0
 0
 0
 1.21833469817642E-04
 0
 0
 0
 0
 1.39265330900937E-04
 1.60564116975691E-04
 5.83250322770877E-04

 2.18403339467057E-04
 0
 0
 1.52186759860312E-04
 0
 0
 0
 1.21833469817642E-04
 0
 0
 0
 0
 1.39265330900937E-04
 1.60564116975691E-04
 5.83250322770877E-04

 2.18403339467057E-04
 0
 0
 1.52186759860312E-04
 0
 0
 0
 1.21833469817642E-04
 0
 0
 0
 0
 1.39265330900937E-04
 1.60564116975691E-04
 5.83250322770877E-04

 2.18403339467057E-04
 0
 0
 1.52186759860312E-04
 0
 0
 0
 1.21833469817642E-04
 0
 0
 0
 0
 1.39265330900937E-04
 1.60564116975691E-04
 5.83250322770877E-04

 .010246180197237
 1.25480565345739E-02
 1.39783180700458E-02
 1.00897755903569E-02
 4.92093495386988E-03
 8.85601040110551E-03
 9.15553210174718E-03
 .010736781258661
 4.60386727064107E-02
 9.08700285427807E-03
 9.90611919567077E-03
 1.23025422554073E-02
 1.59027144371745E-02
 .013827834294954
 1.61715637975034E-02
 4.95191466534736E-02
 5.19639147848182E-02
 6.08241653298892E-02
 1.79334470416994E-02
 1.64570493243545E-02
 2.24551064552174E-02

 3.02404623877463E-04
 3.02577249770473E-04
 3.63350775521606E-04
 2.10720129037356E-04
 5.78933523984423E-04
 0
 2.64251350984672E-04
 3.37384993340467E-04
 6.18701530441594E-04
 3.75861431928678E-04
 9.71188156438882E-05
 4.74759022399615E-04
 0
 2.37379511199808E-04
 0
 0
 1.55742414862792E-04
 1.9520318865241E-04
 0
 0
 2.79149403830627E-04

 3.02404623877463E-04
 3.02577249770473E-04
 3.63350775521606E-04
 2.10720129037356E-04
 5.78933523984423E-04
 0
 2.64251350984672E-04
 3.37384993340467E-04
 6.18701530441594E-04
 3.75861431928678E-04
 9.71188156438882E-05
 4.74759022399615E-04
 0
 2.37379511199808E-04
 0
 0
 1.55742414862792E-04
 1.9520318865241E-04
 0
 0
 2.79149403830627E-04

 3.02404623877463E-04
 3.02577249770473E-04
 3.63350775521606E-04
 2.10720129037356E-04
 5.78933523984423E-04
 0
 2.64251350984672E-04
 3.37384993340467E-04
 6.18701530441594E-04
 3.75861431928678E-04
 9.71188156438882E-05
 4.74759022399615E-04
 0
 2.37379511199808E-04
 0
 0
 1.55742414862792E-04
 1.9520318865241E-04
 0
 0
 2.79149403830627E-04

 3.02404623877463E-04
 3.02577249770473E-04
 3.63350775521606E-04
 2.10720129037356E-04
 5.78933523984423E-04
 0
 2.64251350984672E-04
 3.37384993340467E-04
 6.18701530441594E-04
 3.75861431928678E-04
 9.71188156438882E-05
 4.74759022399615E-04
 0
 2.37379511199808E-04
 0
 0
 1.55742414862792E-04
 1.9520318865241E-04
 0
 0
 2.79149403830627E-04

 9.94377557335954E-03
 1.13377475354933E-02
 1.36149672945242E-02
 9.66833533228224E-03
 3.76306790590103E-03
 8.85601040110551E-03
 8.89128075076251E-03
 1.00620112719801E-02
 4.54199711759691E-02
 8.33527999042072E-03
 .008837812223588
 5.50161925955968E-03
 9.52801720915238E-03
 5.44479942953201E-03
 9.78398208788878E-03
 .014167794376609
 1.58215970863212E-02
 1.98877601614693E-02
 7.72177990696791E-03
 1.42658723345801E-02
 1.89246875244183E-02

 9.94377557335954E-03
 1.13377475354933E-02
 1.36149672945242E-02
 9.66833533228224E-03
 3.76306790590103E-03
 8.85601040110551E-03
 8.89128075076251E-03
 1.00620112719801E-02
 4.54199711759691E-02
 8.33527999042072E-03
 .008837812223588
 5.50161925955968E-03
 9.52801720915238E-03
 5.44479942953201E-03
 9.78398208788878E-03
 .014167794376609
 1.58215970863212E-02
 1.98877601614693E-02
 7.72177990696791E-03
 1.42658723345801E-02
 1.89246875244183E-02

 9.94377557335954E-03
 1.13377475354933E-02
 1.36149672945242E-02
 9.66833533228224E-03
 3.76306790590103E-03
 8.85601040110551E-03
 8.89128075076251E-03
 1.00620112719801E-02
 4.54199711759691E-02
 8.33527999042072E-03
 .008837812223588
 5.50161925955968E-03
 9.52801720915238E-03
 5.44479942953201E-03
 9.78398208788878E-03
 .014167794376609
 1.58215970863212E-02
 1.98877601614693E-02
 7.72177990696791E-03
 1.42658723345801E-02
 1.89246875244183E-02

 4.62501189458694E-04
 0
 0
 0
 4.42713871282206E-04
 4.3554149513671E-04
 0
 5.16000578050126E-04
 2.36562349875215E-04
 7.18558619865134E-04
 1.48534659219758E-04
 0
 0
 0
 0
 9.37839854066886E-03
 9.8850626844999E-03
 1.47780296349809E-02
 3.38267407582146E-03
 1.08137807784073E-02
 1.53696377638633E-02

 1.38750356837513E-03
 1.61967821935195E-03
 2.77856475398875E-04
 9.66833533227558E-04
 2.2135693564156E-04
 8.71082990272221E-04
 1.01037281258845E-03
 5.16000578050126E-04
 3.78499759799954E-03
 2.8742344794546E-04
 7.42673296100322E-04
 0
 0
 0
 0
 0
 1.19097140777429E-04
 0
 9.14236236710066E-05
 7.2091871855807E-04
 1.17406955140682E-03

 6.93751784188519E-04
 6.94147808296012E-04
 1.11142590159321E-03
 1.12797245542993E-03
 1.3281416138457E-03
 0
 4.04149125034546E-04
 1.16100130061198E-03
 3.78499759799954E-03
 7.18558619865134E-04
 3.71336648050161E-04
 1.81525508564227E-04
 5.8982963675799E-04
 2.60771819315198E-04
 2.94914818378995E-04
 2.60511070574468E-04
 2.38194281554173E-04
 4.47819079848789E-04
 0
 7.2091871855807E-04
 1.06733595582606E-04

 2.08125535256269E-03
 3.93350424700851E-03
 3.61213418018308E-03
 4.35075089953067E-03
 6.64070806923765E-04
 5.08131744325662E-03
 3.8394166878311E-03
 4.3860049134266E-03
 3.28821666326002E-02
 3.01794620342525E-03
 5.79285170957883E-03
 3.63051017129117E-04
 7.3728704594479E-04
 3.51534573597644E-04
 7.76516368491543E-04
 2.60511070574468E-04
 0
 4.47819079848789E-04
 9.14236236710066E-05
 0
 0

 5.31876367877451E-03
 5.09041726083679E-03
 8.61355073734907E-03
 3.22277844409408E-03
 1.1067846782078E-03
 2.46806847243996E-03
 3.63734212530841E-03
 3.48300390184128E-03
 4.73124699749454E-03
 3.59279309931973E-03
 1.78241591063893E-03
 1.63372957707771E-03
 5.30846673081651E-03
 1.83691917873612E-03
 6.73296182059974E-03
 2.60511070574468E-04
 2.38194281554173E-04
 0
 9.14236236710066E-05
 3.60459359278517E-04
 3.20200786747203E-04

 0
 0
 0
 0
 0
 0
 0
 0
 0
 0
 0
 3.32331315678862E-03
 2.89243379563309E-03
 2.99557385788304E-03
 1.97958908041851E-03

 0
 0
 0
 0
 0
 0
 0
 0
 0
 0
 0
 0
 0
 0
 0
 3.74735155364224E-03
 4.98375727560396E-03
 3.31845420708985E-03
 4.06483496013343E-03
 1.64979475977814E-03
 1.95404582681841E-03

 0
 0
 0
 0
 0
 0
 0
 0
 0
 0
 0
 0
 0
 0
 0
 2.60511070574468E-04
 3.57291422331602E-04
 8.95638159701012E-04
 0
 0
 0

 0
 9.0773174931017E-04
 0
 2.10720129037356E-04
 5.78933523984423E-04
 0
 0
 3.37384993340467E-04
 0
 3.75861431928678E-04
 9.71188156438882E-04
 0
 7.71315678837371E-04
 2.22319546581727E-04
 1.27461147708537E-03
 4.42868819976792E-03
 5.29524210532417E-03
 8.39373711204354E-03
 7.17323816496965E-04
 2.35684965682785E-04
 1.11659761532251E-03

 0
 9.0773174931017E-04
 0
 2.10720129037356E-04
 5.78933523984423E-04
 0
 0
 3.37384993340467E-04
 0
 3.75861431928678E-04
 9.71188156438882E-04
 0
 7.71315678837371E-04
 2.22319546581727E-04
 1.27461147708537E-03
 4.42868819976792E-03
 5.29524210532417E-03
 8.39373711204354E-03
 7.17323816496965E-04
 2.35684965682785E-04
 1.11659761532251E-03

 0
 9.0773174931017E-04
 0
 2.10720129037356E-04
 5.78933523984423E-04
 0
 0
 3.37384993340467E-04
 0
 3.75861431928678E-04
 9.71188156438882E-04
 0
 7.71315678837371E-04
 2.22319546581727E-04
 1.27461147708537E-03
 4.42868819976792E-03
 5.29524210532417E-03
 8.39373711204354E-03
 7.17323816496965E-04
 2.35684965682785E-04
 1.11659761532251E-03

 0
 9.0773174931017E-04
 0
 2.10720129037356E-04
 5.78933523984423E-04
 0
 0
 3.37384993340467E-04
 0
 3.75861431928678E-04
 9.71188156438882E-04
 0
 7.71315678837371E-04
 2.22319546581727E-04
 1.27461147708537E-03
 4.42868819976792E-03
 5.29524210532417E-03
 8.39373711204354E-03
 7.17323816496965E-04
 2.35684965682785E-04
 1.11659761532251E-03

 0
 0
 0
 0
 0
 0
 0
 0
 0
 0
 0
 6.17186729116844E-03
 5.60338154918472E-03
 7.84618746650065E-03
 5.11297023252923E-03
 3.04797952571214E-02
 .030488868038989
 3.20937007224765E-02
 9.41663323811421E-03
 1.80229679639777E-03
 2.13467191164597E-03

 0
 0
 0
 0
 0
 0
 0
 0
 0
 0
 0
 6.17186729116844E-03
 5.60338154918472E-03
 7.84618746650065E-03
 5.11297023252923E-03
 3.04797952571214E-02
 .030488868038989
 3.20937007224765E-02
 9.41663323811421E-03
 1.80229679639777E-03
 2.13467191164597E-03

 0
 0
 0
 0
 0
 0
 0
 0
 0
 0
 0
 6.17186729116844E-03
 5.60338154918472E-03
 7.84618746650065E-03
 5.11297023252923E-03
 3.04797952571214E-02
 .030488868038989
 3.20937007224765E-02
 9.41663323811421E-03
 1.80229679639777E-03
 2.13467191164597E-03

 0
 0
 0
 0
 0
 0
 0
 0
 0
 0
 0
 6.17186729116844E-03
 5.60338154918472E-03
 7.84618746650065E-03
 5.11297023252923E-03
 3.04797952571214E-02
 .030488868038989
 3.20937007224765E-02
 9.41663323811421E-03
 1.80229679639777E-03
 2.13467191164597E-03

 0
 0
 0
 0
 0
 0
 0
 0
 0
 0
 0
 1.54296682279593E-04
 0
 7.71483411397964E-05
 0
 4.42868819975322E-04
 2.02465139321047E-04
 2.53764145247404E-04
 7.77100801203556E-05
 1.5319522769381E-04
 0

 0
 0
 0
 0
 0
 0
 0
 0
 0
 0
 0
 1.54296682279593E-04
 0
 7.71483411397964E-05
 0
 4.42868819975322E-04
 2.02465139321047E-04
 2.53764145247404E-04
 7.77100801203556E-05
 1.5319522769381E-04
 0

 0
 0
 0
 0
 0
 0
 0
 0
 0
 0
 0
 1.54296682279593E-04
 0
 7.71483411397964E-05
 0
 4.42868819975322E-04
 2.02465139321047E-04
 2.53764145247404E-04
 7.77100801203556E-05
 1.5319522769381E-04
 0

 0
 0
 0
 0
 0
 0
 0
 0
 0
 0
 0
 1.54296682279593E-04
 0
 7.71483411397964E-05
 0
 4.42868819975322E-04
 2.02465139321047E-04
 2.53764145247404E-04
 7.77100801203556E-05
 1.5319522769381E-04
 0

 3.08603918666074E-02
 4.60219996899842E-02
 2.76328264783625E-02
 .036159574142739
 2.42717879930877E-02
 4.05997263716607E-02
 5.08419599293416E-02
 5.53733120318893E-02
 7.39967030408304E-02
 2.80956420366697E-02
 3.91388827044974E-02
 .113870951522212
 8.96172404347658E-02
 9.10392942067972E-02
 .145349276637715
 .1618685537012
 7.90626369049723E-02
 6.59786777644447E-02
 3.86996198998593E-02
 2.23665032432214E-02
 2.81243024359644E-02

 2.96810138334886E-02
 4.56286492652834E-02
 2.69242924660964E-02
 3.50638294717458E-02
 2.40836345977924E-02
 4.03529195244167E-02
 4.94678529042227E-02
 5.49347115405467E-02
 7.35945470460433E-02
 2.78513321059161E-02
 3.86969920933198E-02
 1.77441184621433E-02
 3.88550273212721E-03
 .013929828915802
 4.94741466138377E-03
 .128431957793034
 5.78037972762419E-02
 4.51700178541328E-02
 3.13948723685647E-02
 2.22133080155276E-02
 2.64005548673038E-02

 2.96810138334886E-02
 4.56286492652834E-02
 2.69242924660964E-02
 3.50638294717458E-02
 2.40836345977924E-02
 4.03529195244167E-02
 4.94678529042227E-02
 5.49347115405467E-02
 7.35945470460433E-02
 2.78513321059161E-02
 3.86969920933198E-02
 1.77441184621433E-02
 3.88550273212721E-03
 .013929828915802
 4.94741466138377E-03
 .128431957793034
 5.78037972762419E-02
 4.51700178541328E-02
 3.13948723685647E-02
 2.22133080155276E-02
 2.64005548673038E-02

 2.96810138334886E-02
 4.56286492652834E-02
 2.69242924660964E-02
 3.50638294717458E-02
 2.40836345977924E-02
 4.03529195244167E-02
 4.94678529042227E-02
 5.49347115405467E-02
 7.35945470460433E-02
 2.78513321059161E-02
 3.86969920933198E-02
 1.77441184621433E-02
 3.88550273212721E-03
 .013929828915802
 4.94741466138377E-03
 .128431957793034
 5.78037972762419E-02
 4.51700178541328E-02
 3.13948723685647E-02
 2.22133080155276E-02
 2.64005548673038E-02

 2.96810138334886E-02
 4.56286492652834E-02
 2.69242924660964E-02
 3.50638294717458E-02
 2.40836345977924E-02
 4.03529195244167E-02
 4.94678529042227E-02
 5.49347115405467E-02
 7.35945470460433E-02
 2.78513321059161E-02
 3.86969920933198E-02
 1.77441184621433E-02
 3.88550273212721E-03
 .013929828915802
 4.94741466138377E-03
 .128431957793034
 5.78037972762419E-02
 4.51700178541328E-02
 3.13948723685647E-02
 2.22133080155276E-02
 2.64005548673038E-02

 1.17937803311886E-03
 3.93350424700803E-04
 7.08534012266156E-04
 1.09574467099312E-03
 1.88153395295326E-04
 2.46806847243966E-04
 1.37410702511887E-03
 4.38600491342607E-04
 4.02155994787036E-04
 2.44309930753641E-04
 4.41890611177606E-04
 9.35037894613164E-02
 8.44783497245301E-02
 7.47863895546705E-02
 .138503964285414
 2.21434409988298E-03
 1.41725597525024E-03
 6.34410363119604E-04
 8.54810881322571E-04
 0
 5.44341337472332E-04

 1.17937803311886E-03
 3.93350424700803E-04
 7.08534012266156E-04
 1.09574467099312E-03
 1.88153395295326E-04
 2.46806847243966E-04
 1.37410702511887E-03
 4.38600491342607E-04
 4.02155994787036E-04
 2.44309930753641E-04
 4.41890611177606E-04
 9.35037894613164E-02
 8.44783497245301E-02
 7.47863895546705E-02
 .138503964285414
 2.21434409988298E-03
 1.41725597525024E-03
 6.34410363119604E-04
 8.54810881322571E-04
 0
 5.44341337472332E-04

 1.17937803311886E-03
 3.93350424700803E-04
 7.08534012266156E-04
 1.09574467099312E-03
 1.88153395295326E-04
 2.46806847243966E-04
 1.37410702511887E-03
 4.38600491342607E-04
 4.02155994787036E-04
 2.44309930753641E-04
 4.41890611177606E-04
 9.35037894613164E-02
 8.44783497245301E-02
 7.47863895546705E-02
 .138503964285414
 2.21434409988298E-03
 1.41725597525024E-03
 6.34410363119604E-04
 8.54810881322571E-04
 0
 5.44341337472332E-04

 1.17937803311886E-03
 3.93350424700803E-04
 7.08534012266156E-04
 1.09574467099312E-03
 1.88153395295326E-04
 2.46806847243966E-04
 1.37410702511887E-03
 4.38600491342607E-04
 4.02155994787036E-04
 2.44309930753641E-04
 4.41890611177606E-04
 9.35037894613164E-02
 8.44783497245301E-02
 7.47863895546705E-02
 .138503964285414
 2.21434409988298E-03
 1.41725597525024E-03
 6.34410363119604E-04
 8.54810881322571E-04
 0
 5.44341337472332E-04

 0
 0
 0
 0
 0
 0
 0
 0
 0
 0
 0
 2.62304359875223E-03
 5.01355191244291E-04
 1.74504491521127E-03
 8.28497460105492E-04
 3.12222518082825E-02
 1.98415836534801E-02
 2.01742495471923E-02
 6.44993664997209E-03
 1.5319522769381E-04
 1.17940623118831E-03

 0
 0
 0
 0
 0
 0
 0
 0
 0
 0
 0
 2.62304359875223E-03
 5.01355191244291E-04
 1.74504491521127E-03
 8.28497460105492E-04
 3.12222518082825E-02
 1.98415836534801E-02
 2.01742495471923E-02
 6.44993664997209E-03
 1.5319522769381E-04
 1.17940623118831E-03

 0
 0
 0
 0
 0
 0
 0
 0
 0
 0
 0
 2.62304359875223E-03
 5.01355191244291E-04
 1.74504491521127E-03
 8.28497460105492E-04
 3.12222518082825E-02
 1.98415836534801E-02
 2.01742495471923E-02
 6.44993664997209E-03
 1.5319522769381E-04
 1.17940623118831E-03

 0
 0
 0
 0
 0
 0
 0
 0
 0
 0
 0
 2.62304359875223E-03
 5.01355191244291E-04
 1.74504491521127E-03
 8.28497460105492E-04
 3.12222518082825E-02
 1.98415836534801E-02
 2.01742495471923E-02
 6.44993664997209E-03
 1.5319522769381E-04
 1.17940623118831E-03

 0
 0
 0
 0
 0
 0
 0
 0
 0
 0
 0
 0
 7.52032786864145E-04
 5.78030821113546E-04
 1.06940023081251E-03

 0
 0
 0
 0
 0
 0
 0
 0
 0
 0
 0
 0
 7.52032786864145E-04
 5.78030821113546E-04
 1.06940023081251E-03

 0
 0
 0
 0
 0
 0
 0
 0
 0
 0
 0
 0
 7.52032786864145E-04
 5.78030821113546E-04
 1.06940023081251E-03

 0
 0
 0
 0
 0
 0
 0
 0
 0
 0
 0
 0
 7.52032786864145E-04
 5.78030821113546E-04
 1.06940023081251E-03

 1.96563005520351E-04
 0
 0
 0
 0
 1.23403423622238E-04
 0
 0
 4.02155994787036E-04
 0
 0

 1.96563005520351E-04
 0
 0
 0
 0
 1.23403423622238E-04
 0
 0
 4.02155994787036E-04
 0
 0

 1.96563005520351E-04
 0
 0
 0
 0
 1.23403423622238E-04
 0
 0
 4.02155994787036E-04
 0
 0

 1.96563005520351E-04
 0
 0
 0
 0
 1.23403423622238E-04
 0
 0
 4.02155994787036E-04
 0
 0

 1.96563005520351E-04
 0
 0
 0
 0
 1.23403423622238E-04
 0
 0
 4.02155994787036E-04
 0
 0

 8.25564623183201E-03
 2.16342733585563E-02
 1.70048162943839E-02
 5.34175527108792E-02
 5.38118710543513E-02
 3.70210270865694E-03
 2.64515602335543E-02
 7.96059891787098E-02
 1.44776158123399E-02
 .131194432814322
 .141531250037847
 5.55468056206421E-03
 .086233092893798
 6.2455252077081E-03
 6.66915969178466E-02
 4.42868819976596E-04
 4.04930278642094E-04
 1.52258487149172E-03
 3.10840320480529E-02
 .361847127812827
 4.91721674848615E-02

 8.25564623183201E-03
 2.16342733585563E-02
 1.70048162943839E-02
 5.34175527108792E-02
 5.38118710543513E-02
 3.70210270865694E-03
 2.64515602335543E-02
 7.96059891787098E-02
 1.44776158123399E-02
 .131194432814322
 .141531250037847
 5.55468056206421E-03
 .086233092893798
 6.2455252077081E-03
 6.66915969178466E-02
 4.42868819976596E-04
 4.04930278642094E-04
 1.52258487149172E-03
 3.10840320480529E-02
 .361847127812827
 4.91721674848615E-02

 8.25564623183201E-03
 2.16342733585563E-02
 1.70048162943839E-02
 5.34175527108792E-02
 5.38118710543513E-02
 3.70210270865694E-03
 2.64515602335543E-02
 7.96059891787098E-02
 1.44776158123399E-02
 .131194432814322
 .141531250037847
 5.55468056206421E-03
 .086233092893798
 6.2455252077081E-03
 6.66915969178466E-02
 4.42868819976596E-04
 4.04930278642094E-04
 1.52258487149172E-03
 3.10840320480529E-02
 .361847127812827
 4.91721674848615E-02

 8.25564623183201E-03
 2.16342733585563E-02
 1.70048162943839E-02
 5.34175527108792E-02
 5.38118710543513E-02
 3.70210270865694E-03
 2.64515602335543E-02
 7.96059891787098E-02
 1.44776158123399E-02
 .131194432814322
 .141531250037847
 5.55468056206421E-03
 .086233092893798
 6.2455252077081E-03
 6.66915969178466E-02
 4.42868819976596E-04
 4.04930278642094E-04
 1.52258487149172E-03
 3.10840320480529E-02
 .361847127812827
 4.91721674848615E-02

 8.25564623183201E-03
 2.16342733585563E-02
 1.70048162943839E-02
 5.34175527108792E-02
 5.38118710543513E-02
 3.70210270865694E-03
 2.64515602335543E-02
 7.96059891787098E-02
 1.44776158123399E-02
 .131194432814322
 .141531250037847
 5.55468056206421E-03
 .086233092893798
 6.2455252077081E-03
 6.66915969178466E-02
 4.42868819976596E-04
 4.04930278642094E-04
 1.52258487149172E-03
 3.10840320480529E-02
 .361847127812827
 4.91721674848615E-02

 .27385081624651
 .295127854209096
 .292646127726195
 .325741759374748
 .593274918298165
 .155437275639325
 .392734430331662
 .446080592503057
 .279008562054389
 .4585314772455
 .271339958805984
 .279041896692705
 .202865269457929
 .311839942941183
 .194493520908059
 .369268984687502
 .482975942341469
 .445355919392349
 .568411073625784
 .267120649962093
 .17650084892965

 5.61608587201003E-05
 5.61929178145164E-05
 0
 5.47872335496155E-04
 1.07516225882821E-04
 0
 1.47225752691257E-04
 2.19300245670721E-04
 5.74508563982666E-04
 3.14112768111608E-04
 7.21454059067394E-05
 4.40847663655979E-05
 7.16221701777559E-05
 2.2042383182799E-05
 1.34865919000671E-04
 6.32669742823708E-04
 5.20624643969239E-04
 6.52536373494367E-04
 4.21854720652465E-04
 0
 0

 5.61608587201003E-05
 5.61929178145164E-05
 0
 5.47872335496155E-04
 1.07516225882821E-04
 0
 1.47225752691257E-04
 2.19300245670721E-04
 5.74508563982666E-04
 3.14112768111608E-04
 7.21454059067394E-05
 4.40847663655979E-05
 7.16221701777559E-05
 2.2042383182799E-05
 1.34865919000671E-04
 6.32669742823708E-04
 5.20624643969239E-04
 6.52536373494367E-04
 4.21854720652465E-04
 0
 0

 5.61608587201003E-05
 5.61929178145164E-05
 0
 5.47872335496155E-04
 1.07516225882821E-04
 0
 1.47225752691257E-04
 2.19300245670721E-04
 5.74508563982666E-04
 3.14112768111608E-04
 7.21454059067394E-05
 4.40847663655979E-05
 7.16221701777559E-05
 2.2042383182799E-05
 1.34865919000671E-04
 6.32669742823708E-04
 5.20624643969239E-04
 6.52536373494367E-04
 4.21854720652465E-04
 0
 0

 5.61608587201003E-05
 5.61929178145164E-05
 0
 5.47872335496155E-04
 1.07516225882821E-04
 0
 1.47225752691257E-04
 2.19300245670721E-04
 5.74508563982666E-04
 3.14112768111608E-04
 7.21454059067394E-05
 4.40847663655979E-05
 7.16221701777559E-05
 2.2042383182799E-05
 1.34865919000671E-04
 6.32669742823708E-04
 5.20624643969239E-04
 6.52536373494367E-04
 4.21854720652465E-04
 0
 0

 0
 1.00141395282986E-04
 2.06898504530602E-03
 8.0569461102352E-05
 0
 3.4813073673714E-03
 5.05186406294225E-05
 3.22500361281994E-05
 8.797162385969E-04
 0
 0
 2.25474700499878E-04
 1.43629405786509E-04
 3.75406263557425E-04
 1.21519637472649E-04
 1.14171436348646E-04
 2.20085258179047E-04
 1.23756898421336E-04
 1.91659343744883E-04
 1.66527488888586E-04
 2.59210160700614E-04

 0
 4.22957445915715E-05
 1.93005680760687E-03
 0
 0
 3.44501224277663E-03
 0
 0
 8.797162385969E-04
 0
 0
 2.25474700499878E-04
 1.43629405786509E-04
 3.75406263557425E-04
 1.21519637472649E-04
 1.14171436348646E-04
 2.20085258179047E-04
 1.23756898421336E-04
 1.91659343744883E-04
 1.66527488888586E-04
 2.59210160700614E-04

 0
 0
 0
 0
 0
 1.54254279527479E-04
 0
 0
 8.797162385969E-04
 0
 0
 8.81695327313571E-05
 3.58110850888124E-05
 2.09236429540977E-04
 1.79055425444062E-05
 1.14171436348646E-04
 2.20085258179047E-04
 1.23756898421336E-04
 1.91659343744883E-04
 1.66527488888586E-04
 2.59210160700614E-04

 0
 0
 0
 0
 0
 1.54254279527479E-04
 0
 0
 8.797162385969E-04
 0
 0
 8.81695327313571E-05
 3.58110850888124E-05
 2.09236429540977E-04
 1.79055425444062E-05
 6.32669742823708E-05
 1.73541547990301E-04
 3.62520207497334E-05
 6.66086401030343E-05
 1.31310195165871E-04
 2.59210160700614E-04

 0
 0
 0
 0
 0
 0
 0
 0
 0
 0
 0
 0
 0
 0
 0
 5.09044620662754E-05
 4.65437101887465E-05
 8.75048776716025E-05
 1.25050703641849E-04
 3.5217293722715E-05
 0

 0
 4.22957445915715E-05
 1.93005680760687E-03
 0
 0
 3.29075796324915E-03
 0
 0
 0
 0
 0
 7.09410033470689E-05
 0
 1.01910825939459E-04
 0

 0
 4.22957445915715E-05
 1.93005680760687E-03
 0
 0
 3.29075796324915E-03
 0
 0
 0
 0
 0
 7.09410033470689E-05
 0
 1.01910825939459E-04
 0

 0
 0
 0
 0
 0
 0
 0
 0
 0
 0
 0
 6.63641644214516E-05
 1.07818320697697E-04
 6.42590080769886E-05
 1.03614094928243E-04

 0
 0
 0
 0
 0
 0
 0
 0
 0
 0
 0
 6.63641644214516E-05
 1.07818320697697E-04
 6.42590080769886E-05
 1.03614094928243E-04

 0
 5.7845650691414E-05
 1.38928237699151E-04
 8.0569461102352E-05
 0
 3.62951245947758E-05
 5.05186406294225E-05
 3.22500361281994E-05
 0
 0
 0

 0
 5.7845650691414E-05
 1.38928237699151E-04
 8.0569461102352E-05
 0
 3.62951245947758E-05
 5.05186406294225E-05
 3.22500361281994E-05
 0
 0
 0

 0
 5.7845650691414E-05
 1.38928237699151E-04
 8.0569461102352E-05
 0
 3.62951245947758E-05
 5.05186406294225E-05
 3.22500361281994E-05
 0
 0
 0

 .271492060180269
 .293285732361565
 .28875519807792
 .317443104881202
 .589619366618148
 .150898224640907
 .388168988608498
 .435803888133429
 .268362200228103
 .45231902472062
 .26435989078451
 .256316659358448
 .171660000123169
 .291399685338778
 .166184862631517
 .2594301089161
 .363306848057141
 .329557842987823
 .551927863532508
 .265101731915948
 .172928521331501

 .254531480846853
 .274341694943396
 .265179572312668
 .292700798872801
 .580446888597534
 .140076185115761
 .37395863627061
 .42565733569531
 .249665537149014
 .444821763720628
 .254091570434431
 .25307642903058
 .168474462744973
 .288582220616826
 .163344241983198
 .2556519883532
 .359349358644515
 .323888575492889
 .547543525083618
 .265101731915948
 .172928521331501

 0
 0
 6.21521063392221E-05
 1.44176930393682E-04
 4.95140513935068E-05
 6.49491703273594E-05
 1.80803555936507E-04
 8.65658864492583E-05
 1.58745787416044E-04
 3.21460435202823E-05
 9.96745739500319E-05
 0
 0
 0
 0
 5.31442583970896E-04
 8.90846613013772E-04
 1.11656223909004E-03
 1.61636966650054E-03
 0
 1.45157689991926E-04

 0
 0
 6.21521063392221E-05
 1.44176930393682E-04
 4.95140513935068E-05
 6.49491703273594E-05
 1.80803555936507E-04
 8.65658864492583E-05
 1.58745787416044E-04
 3.21460435202823E-05
 9.96745739500319E-05
 0
 0
 0
 0
 5.31442583970896E-04
 8.90846613013772E-04
 1.11656223909004E-03
 1.61636966650054E-03
 0
 1.45157689991926E-04

 1.34490477460994E-03
 1.60445567970096E-03
 1.18089002044368E-03
 6.88444842630303E-03
 1.28736533622831E-03
 1.52630550269771E-03
 5.01729867724022E-03
 1.35619888770834E-03
 5.76776360944969E-03
 6.10774826884567E-04
 1.06319545546609E-03
 0
 1.51143844418763E-03
 0
 1.97933775865002E-03
 0
 0
 0
 5.01355355614236E-05
 0
 0

 1.34490477460994E-03
 1.60445567970096E-03
 1.18089002044368E-03
 6.88444842630303E-03
 1.28736533622831E-03
 1.52630550269771E-03
 5.01729867724022E-03
 1.35619888770834E-03
 5.76776360944969E-03
 6.10774826884567E-04
 1.06319545546609E-03
 0
 1.51143844418763E-03
 0
 1.97933775865002E-03
 0
 0
 0
 5.01355355614236E-05
 0
 0

 1.83458805152274E-03
 1.96675212350266E-03
 9.44712016356176E-04
 2.92198578931121E-03
 5.01742387453166E-04
 7.40420541732067E-04
 2.40468729395578E-03
 1.97370221104339E-03
 4.15561194613575E-03
 7.32929792261764E-04
 5.47102661459701E-04
 2.59137217449648E-02
 7.12452113872863E-04
 .026986784398963
 5.69107059641045E-04
 2.71160031880005E-03
 1.58952894467182E-03
 2.48866942445522E-03
 2.92707968453108E-03
 7.95540129777795E-04
 3.85177203707681E-04

 3.93126011040161E-04
 7.86700849401064E-04
 7.87260013630147E-04
 1.55230495057217E-03
 0
 3.29075796325288E-04
 1.48861594387937E-03
 3.65500409452927E-04
 1.74267597741298E-03
 8.14366435847152E-05
 8.41696402245293E-05
 3.70312037471324E-03
 2.50677595622146E-04
 4.54903735254259E-03
 1.25338797811073E-04
 1.47622939992199E-04
 3.37441898868994E-04
 8.45880484160445E-05
 9.84327681522419E-04
 7.14911062570526E-04
 2.41929483320225E-04

 1.31042003680234E-03
 1.1800512741016E-03
 1.57452002726029E-04
 1.36968083873904E-03
 5.01742387453166E-04
 3.29075796325288E-04
 8.01562431316387E-04
 1.60820180159046E-03
 2.27888397046015E-03
 6.51493148677049E-04
 4.62933021235172E-04
 1.21813170220954E-04
 6.59677883216173E-05
 1.74991615593414E-04
 3.29838941608086E-05
 3.49633278929562E-04
 7.99204497320689E-05
 1.00170057334598E-04
 2.04500210843041E-05
 4.03145336036342E-05
 1.43247720387456E-04

 1.31042003680234E-04
 0
 0
 0
 0
 8.22689490814919E-05
 1.14508918760024E-04
 0
 1.34051998262622E-04
 0
 0
 2.20887882000307E-02
 3.958067299291E-04
 .022262755430827
 4.10784367669163E-04
 2.21434409987829E-03
 1.17216659607075E-03
 2.30391131870458E-03
 1.92230198192435E-03
 4.03145336036342E-05
 0

 2.927534124766E-04
 0
 5.02506391678817E-05
 6.41127201113296E-04
 8.00652745935904E-05
 2.62560475791995E-05
 3.65453996041273E-04
 4.66596267386715E-05
 5.98955736916508E-04
 2.59904181653346E-05
 1.07450604542119E-04
 2.38557991042583E-04
 5.54690849885773E-04
 3.07856426522698E-04
 6.62558667931784E-04
 1.94736644245028E-04
 8.52938246500657E-04
 5.07528290495294E-04
 1.03613440160176E-04
 7.14911062569939E-04
 4.4267948011919E-04

 4.18219160681598E-05
 0
 0
 2.91421455051662E-05
 0
 0
 3.65453996042631E-05
 2.33298133693358E-05
 0
 0
 0

 8.36438321361469E-05
 0
 0
 1.45710727525831E-04
 0
 0
 0
 2.33298133693358E-05
 2.99477868457636E-04
 0
 4.02939767032599E-05
 0
 5.01355191242763E-04
 9.63384701854148E-05
 6.35890838610279E-04
 0
 6.07395417962171E-04
 3.38352193663692E-04
 1.03613440160176E-04
 4.08520607182906E-04
 3.62894224981555E-04

 0
 0
 0
 3.7884789156692E-04
 0
 0
 2.55817797228634E-04
 0
 2.13912763185035E-04
 0
 0

 1.67287664272294E-04
 0
 5.02506391678817E-05
 8.74264365153783E-05
 8.00652745935904E-05
 2.62560475791995E-05
 7.30907992083753E-05
 0
 8.55651052738374E-05
 2.59904181653346E-05
 6.71566278388589E-05
 2.057289097065E-04
 0
 1.0286445485325E-04
 0
 1.47622939992199E-04
 2.02465139321241E-04
 1.69176096831603E-04
 0
 3.06390455387033E-04
 6.04823708301432E-05

 0
 0
 0
 0
 0
 0
 0
 0
 0
 0
 0
 3.28290813360836E-05
 5.33356586430097E-05
 1.08653501484033E-04
 2.66678293215048E-05
 4.71137042528293E-05
 4.30776892172441E-05
 0
 0
 0
 1.93028843074925E-05

 0
 8.36915797235751E-05
 0
 0
 0
 2.62560475791995E-05
 0
 0
 0
 0
 0
 0
 0
 0
 0
 0
 8.6155378434612E-05
 1.61977113988481E-04
 1.65340596000757E-05
 0
 1.93028843074925E-04

 0
 8.36915797235751E-05
 0
 0
 0
 2.62560475791995E-05
 0
 0
 0
 0
 0
 0
 0
 0
 0
 0
 8.6155378434612E-05
 1.61977113988481E-04
 1.65340596000757E-05
 0
 1.93028843074925E-04

 0
 0
 0
 3.60442325984951E-05
 0
 0
 0
 0
 5.29152624720877E-05
 0
 0
 6.56581626722872E-05
 0
 3.28290813361436E-05
 0
 0
 6.4616533825928E-05
 2.69961856646951E-05
 0
 0
 0

 0
 0
 0
 3.60442325984951E-05
 0
 0
 0
 0
 5.29152624720877E-05
 0
 0
 6.56581626722872E-05
 0
 3.28290813361436E-05
 0
 0
 6.4616533825928E-05
 2.69961856646951E-05
 0
 0
 0

 4.66420691063038E-04
 3.33347817543742E-04
 2.40181021107172E-04
 0
 0
 4.18316690244874E-05
 0
 0
 0
 0
 0
 0
 0
 0
 0

 4.66420691063038E-04
 3.33347817543742E-04
 2.40181021107172E-04
 0
 0
 4.18316690244874E-05
 0
 0
 0
 0
 0
 0
 0
 0
 0

 8.91085625021694E-03
 8.39147572693746E-03
 1.00769281744295E-02
 7.66540679927241E-04
 3.01045432472211E-03
 3.76705187898989E-04
 5.03236564023372E-04
 2.48540278427507E-03
 1.8696726073471E-04
 1.38442294093747E-03
 1.26254460336881E-03
 2.09215840379491E-04
 2.96855047446674E-04
 1.04607920189746E-04
 3.91720098242919E-04
 1.6712030942465E-04
 2.82486117921725E-04
 3.06180175249267E-04
 4.09000421684906E-05
 0
 0

 0
 0
 0
 0
 0
 9.74237554911062E-05
 0
 0
 0
 0
 0
 0
 2.30887259125057E-04
 0
 3.28324632267142E-04

 8.91085625021694E-03
 8.39147572693746E-03
 1.00769281744295E-02
 7.30496447328746E-04
 3.01045432472211E-03
 2.46806847244136E-04
 4.58035675039152E-04
 2.48540278427507E-03
 1.34051998262622E-04
 1.38442294093747E-03
 1.26254460336881E-03
 0
 6.59677883216173E-05
 0
 6.33954659757772E-05
 0
 5.32802998213282E-05
 6.6780038223001E-05
 4.09000421684906E-05
 0
 0

 0
 0
 0
 3.60442325984951E-05
 0
 3.24745851637468E-05
 4.52008889842202E-05
 0
 5.29152624720877E-05
 0
 0

 0
 0
 0
 0
 0
 0
 0
 0
 0
 0
 0
 2.09215840379491E-04
 0
 1.04607920189746E-04
 0

 0
 0
 0
 0
 0
 0
 0
 0
 0
 0
 0
 0
 0
 0
 0
 1.6712030942465E-04
 2.29205818100397E-04
 2.39400137026266E-04
 0
 0
 0

 6.72452387305823E-04
 6.21079617949064E-04
 6.83673169731443E-04
 9.37150047557894E-04
 1.63396369598327E-03
 3.57220436800812E-04
 1.0848213356196E-03
 1.73131772898445E-03
 7.93728937080878E-04
 1.25369569728902E-03
 1.47850618025979E-03
 8.04237619390427E-03
 3.17305061826613E-03
 7.15979404667037E-03
 2.4644060155253E-03
 3.99397748962164E-02
 3.76922601036387E-02
 3.84118779859066E-02
 3.55571333264977E-02
 1.12343166975285E-03
 5.07733586704285E-04

 5.17271067158819E-05
 0
 6.21521063392221E-05
 3.60442325984951E-05
 0
 1.62372925818734E-04
 9.04017779682536E-05
 5.77105909661325E-05
 1.05830524943957E-04
 6.42920870404319E-05
 3.32248579833668E-05
 7.92056302368331E-03
 3.00813114746269E-03
 6.94677408758265E-03
 2.35153470830861E-03

 1.55181320147432E-04
 5.17566348291599E-05
 0
 2.52309628188274E-04
 0
 6.49491703273594E-05
 2.71205333904574E-04
 0
 1.05830524943957E-04
 3.21460435202823E-05
 1.66124289917177E-05
 0
 0
 0
 0
 1.74816639464111E-04
 4.52882548482439E-04
 4.67460267561967E-04
 6.13500632527947E-05
 0
 2.38746200645302E-05

 5.17271067158819E-05
 0
 0
 0
 0
 0
 0
 5.77105909661325E-05
 5.29152624720877E-05
 0
 0

 4.13816853726628E-04
 5.69322983119904E-04
 6.21521063392221E-04
 6.48796186771125E-04
 1.63396369598327E-03
 1.29898340654719E-04
 7.23214223746776E-04
 1.61589654705219E-03
 5.29152624720877E-04
 1.1572575667283E-03
 1.42866889328471E-03
 1.21813170220954E-04
 1.6491947080344E-04
 2.13019959087726E-04
 1.12871307216689E-04
 3.94153249778228E-02
 3.71860972553349E-02
 3.78108576418984E-02
 3.53321830945709E-02
 1.12343166975285E-03
 4.83858966639754E-04

 0
 0
 0
 0
 0
 0
 0
 0
 0
 0
 0
 0
 0
 0
 0
 3.49633278929562E-04
 5.32802998213282E-05
 1.33560076446194E-04
 1.63600168673962E-04
 0
 0

 1.03454213431636E-03
 1.03513269658277E-03
 1.05658580776575E-03
 7.20884651968561E-04
 1.48542154180316E-04
 7.14440873600954E-04
 5.87611556794675E-04
 3.17408250314026E-04
 1.16413577438418E-03
 2.89314391681479E-04
 2.99023721850645E-04
 1.33994487242529E-03
 1.88008196716308E-03
 1.24039758862664E-03
 2.15650385617917E-03
 1.22371647625011E-03
 6.3936359785747E-04
 8.68140496899973E-04
 9.20250948791921E-04
 5.24088936845854E-04
 5.01367021354723E-04

 6.72452387306037E-04
 2.58783174145799E-04
 4.97216850713263E-04
 6.12751954173225E-04
 1.48542154180316E-04
 6.49491703273594E-04
 4.52008889842202E-04
 5.77105909661325E-05
 8.46644199552528E-04
 2.25022304640914E-04
 1.66124289917177E-04
 1.25873609227799E-03
 1.81411417884146E-03
 1.12373651156436E-03
 2.06269681838831E-03
 7.57538771012933E-04
 4.52882548482439E-04
 6.01020344007969E-04
 9.20250948791921E-04
 4.8377440324222E-04
 5.01367021354723E-04

 5.17271067158819E-05
 5.17566348291599E-05
 6.21521063392221E-05
 0
 0
 0
 4.52008889842202E-05
 1.44276477415629E-04
 0
 3.21460435202823E-05
 6.64497159667337E-05

 0
 5.17566348291599E-05
 0
 0
 0
 0
 0
 0
 0
 3.21460435202823E-05
 3.32248579833668E-05

 3.10362640294437E-04
 6.72836252778651E-04
 4.97216850713263E-04
 1.08132697795336E-04
 0
 6.49491703273594E-05
 9.04017779682536E-05
 1.15421181932265E-04
 3.17491574831652E-04
 0
 3.32248579833668E-05
 8.12087801473026E-05
 6.59677883216173E-05
 1.16661077062276E-04
 9.3807037790857E-05
 4.66177705237181E-04
 1.86481049375032E-04
 2.67120152892004E-04
 0
 4.03145336036342E-05
 0

 0
 6.66695635087483E-05
 0
 4.64298589404343E-05
 6.37808119645173E-05
 0
 0
 3.71695331647044E-05
 2.04486099044396E-04
 8.28169256792004E-05
 4.27981221480658E-05

 0
 6.66695635087483E-05
 0
 4.64298589404343E-05
 0
 0
 0
 0
 0
 0
 0

 0
 0
 0
 0
 6.37808119645173E-05
 0
 0
 3.71695331647044E-05
 2.04486099044396E-04
 8.28169256792004E-05
 4.27981221480658E-05

 8.9182537827098E-03
 1.64798319510077E-02
 1.51238574231367E-02
 1.15819033503884E-02
 .014566714474447
 1.80637843036799E-03
 5.15290134419011E-03
 .010518551568368
 2.72428254533234E-03
 4.45274551213022E-03
 1.18873778443808E-02
 8.23052442145677E-03
 2.18544898729935E-03
 7.72961773082199E-03
 .002085863967199
 4.58233051317003E-02
 5.43132501502459E-02
 5.63344195347333E-02
 4.76023716594499E-03
 9.88356307697738E-04
 2.39978439099976E-03

 0
 0
 7.61864529319497E-05
 8.83665057251602E-05
 0
 0
 1.10815082670762E-04
 0
 0
 0
 0
 0
 4.04318702615624E-05
 0
 2.02159351307812E-05
 1.30003427799557E-02
 1.63278338162417E-02
 1.57579348258674E-02
 1.95528588690302E-03
 4.44760338463911E-04
 1.7852054615972E-03

 5.07259369084253E-03
 9.96064785130775E-03
 9.67567952235509E-03
 1.81151336736697E-03
 7.10127330629042E-03
 6.36920896114118E-04
 5.54075413354957E-04
 7.53402456903822E-03
 1.03782192203213E-03
 2.28547999737228E-03
 9.9985387137503E-03
 6.41549363162502E-03
 9.89516824824259E-05
 6.44015601282557E-03
 4.94758412412129E-05
 1.16544426309295E-04
 2.93041649017688E-04
 1.00170057334598E-04
 1.02250105421403E-04
 0
 0

 0
 1.74822410978135E-04
 4.19872007268544E-04
 1.82624111832123E-04
 0
 5.48459660543279E-05
 0
 4.87333879270569E-05
 0
 0
 5.61130934830196E-05
 2.84230730514816E-04
 4.28790624089909E-04
 1.80143708751651E-04
 3.36041599305051E-04

 0
 1.26887233774452E-04
 7.61864529319497E-05
 4.41832528626714E-05
 1.8208393093071E-04
 0
 1.10815082670762E-04
 7.07420147326785E-05
 0
 1.18214482622811E-04
 6.10908679049424E-05
 1.49319369948266E-04
 0
 7.4659684974133E-05
 0
 5.71443638677835E-04
 3.91868011589124E-04
 3.68367307616611E-04
 1.50406606684848E-04
 9.88356307699159E-05
 0

 1.26814842270932E-03
 2.47430105860274E-03
 1.44754260570516E-03
 2.12079613740969E-03
 6.73710544443451E-03
 3.98075560071735E-05
 1.16355836804221E-03
 2.22837346407638E-03
 7.13502571396756E-04
 1.73381241180752E-03
 1.1199992449238E-03
 .000137152606471
 0
 1.32801950025777E-04
 5.13617657319469E-05
 9.84152933281324E-05
 0
 1.69176096831765E-04
 0
 0
 0

 2.94844508280121E-04
 0
 0
 0
 0
 0
 0
 0
 0
 0
 0

 2.28266716087783E-03
 3.74317339634465E-03
 3.42839038194403E-03
 7.33441997519177E-03
 5.46251792791378E-04
 1.07480401219237E-03
 3.21363739745143E-03
 6.36678132593668E-04
 9.72958051903449E-04
 3.15238620327604E-04
 6.51635924318768E-04
 0
 0
 0
 0
 0
 6.53113352648539E-05
 4.09297008464731E-05
 5.01355355613756E-05
 0
 0

 0
 0
 0
 0
 0
 0
 0
 0
 0
 0
 0
 1.24432808289767E-03
 1.61727481046546E-03
 9.01856374244857E-04
 1.62876882579001E-03
 3.09293869434902E-02
 3.66723147513098E-02
 3.94153019152057E-02
 2.38143793916714E-03
 4.44760338463911E-04
 6.14578929402563E-04

 0
 0
 0
 0
 0
 0
 0
 0
 0
 0
 0
 0
 0
 0
 0
 1.10717204993914E-03
 3.99602248660345E-04
 4.00680229338006E-04
 2.04500210843041E-05
 0
 0

 0
 0
 0
 0
 0
 0
 0
 0
 0
 0
 0
 0
 0
 0
 0
 0
 1.63278338162417E-04
 8.18594016927109E-05
 1.00271071122895E-04
 0
 0

 0
 0
 0
 4.56560279580937E-05
 0
 0
 0
 0
 0
 0
 0

 0
 0
 0
 4.56560279580937E-05
 0
 0
 0
 0
 0
 0
 0

 6.62106965962049E-03
 7.86700849402119E-03
 7.7068611860543E-03
 8.79479275400927E-03
 1.02989226898335E-02
 4.87118777455236E-03
 4.52008889841343E-03
 2.44692905696218E-02
 6.66732307147955E-03
 1.32441699303234E-02
 1.28912448975411E-02
 2.48289296204948E-02
 3.93806416346958E-02
 .025326927507674
 3.00106348655283E-02
 1.94170533226876E-02
 .018215102242812
 2.09781232331192E-02
 1.25438230403977E-02
 4.24212931015564E-03
 8.65339454983805E-03

 4.03471432383195E-03
 4.24404405598513E-03
 4.72356008177472E-03
 6.70422726329864E-03
 3.86209600868699E-03
 2.40311930211243E-03
 3.5256693407632E-03
 1.17729605570877E-02
 5.29152624720877E-03
 4.30756983169977E-03
 3.08991179244962E-03
 0
 0
 0
 0
 3.49633278928221E-04
 6.92643897679565E-04
 6.01020344006049E-04
 3.27200337347925E-04
 0
 1.90996960515967E-04

 2.069084268631E-04
 4.14053078632424E-04
 0
 7.20884651969901E-05
 2.37667446688587E-03
 1.42888174720271E-03
 0
 7.09840268882702E-03
 1.05830524944175E-04
 6.04345618179289E-03
 6.87754560255357E-03
 1.19953227191801E-02
 2.83023091830715E-03
 1.20576619035139E-02
 3.57728011335314E-03
 3.35723137723742E-03
 2.54714207534151E-03
 3.39716517024809E-03
 3.03319990147295E-03
 4.94178153851E-05
 5.85313266096477E-05

 0
 4.14053078632424E-04
 6.21521063392221E-04
 2.16265395590673E-04
 0
 0
 1.80803555936507E-04
 0
 0
 0
 0
 6.49670241176935E-03
 5.93710094893349E-03
 6.82301549433751E-03
 4.97571421425723E-03

 0
 0
 0
 0
 1.98056205573618E-04
 0
 0
 1.15421181932265E-04
 0
 0
 1.66124289917177E-04

 0
 0
 0
 7.20884651969901E-05
 0
 6.49491703274936E-05
 0
 0
 0
 0
 0
 2.35505462426732E-03
 2.90917946497162E-02
 1.93809418201851E-03
 2.02632728260737E-02

 2.37944690892544E-03
 2.79485828077121E-03
 2.36178004088736E-03
 1.73012316472598E-03
 3.86209600868699E-03
 9.74237554909721E-04
 8.13616001713722E-04
 5.48250614177484E-03
 1.26996629932661E-03
 2.89314391683072E-03
 2.75766321262075E-03
 0
 6.59677883214966E-05
 0
 9.38070377906855E-05
 0
 0
 0
 8.18000843369812E-05
 0
 0

 0
 0
 0
 0
 0
 0
 0
 0
 0
 0
 0
 3.98184986527799E-03
 1.45554732941743E-03
 4.50815592780407E-03
 1.10056067405349E-03

 0
 0
 0
 0
 0
 0
 0
 0
 0
 0
 0
 0
 0
 0
 0
 4.42868819975747E-04
 2.69953519095118E-04
 8.45880484160445E-05
 1.03613440160176E-04
 0
 0

 0
 0
 0
 0
 0
 0
 0
 0
 0
 0
 0
 0
 0
 0
 0
 1.52673198465462E-02
 1.47053627506958E-02
 .016895349670449
 8.9980092770797E-03
 4.19271149477054E-03
 8.40386626271244E-03

 .224435639703011
 .235892249692918
 .228053480748137
 .259143614689733
 .548805823396735
 .1295242334336
 .354141733048395
 .382635068648643
 .227137705856096
 .422680611198235
 .224379426911481
 6.21084750565893E-03
 9.00328375011989E-03
 5.49360372427213E-03
 8.49893887442281E-03
 2.33088852618859E-03
 1.84562958581643E-03
 1.60539211888383E-03
 6.15136634214099E-04
 3.25741431517365E-04
 1.76863185437751E-03

 4.71751213248193E-04
 3.14680339760642E-04
 5.66827209812925E-04
 1.97234040778422E-03
 1.50522716236261E-04
 1.97445477795173E-04
 5.49642810046982E-04
 0
 4.82587193744775E-04
 9.77239723016582E-05
 1.51505352404257E-04
 6.04842994536432E-03
 2.40650491797627E-03
 5.33633825713635E-03
 2.22021542048085E-03
 0
 8.09860557286517E-05
 2.03011316197923E-04
 0
 1.22556182155048E-04
 0

 .223767325484243
 .235380894140807
 .227297711135052
 .257171274281949
 .548655300680499
 .129228065216907
 .353420326860208
 .382635068648643
 .226655118662351
 .422460732260556
 .224227921559076
 0
 5.27742306571732E-04
 0
 4.46340584176011E-04
 5.82722131547482E-04
 6.92643897679565E-04
 8.68140496901894E-04
 4.90800506021887E-04
 8.06290672072685E-05
 1.57572492425652E-03

 1.96563005520351E-04
 1.96675212350808E-04
 0
 0
 0
 0
 1.71763378140037E-04
 0
 0
 1.22154965377073E-04
 0

 0
 0
 1.88942403271235E-04
 0
 0
 9.87227388977902E-05
 0
 0
 0
 0
 0
 1.62417560294605E-04
 6.0690365255719E-03
 1.57265467135788E-04
 5.83238286976595E-03
 0
 4.85916334372842E-04
 0
 1.24336128192211E-04
 1.22556182155048E-04
 1.45157689991926E-04

 0
 0
 0
 0
 0
 0
 0
 0
 0
 0
 0
 0
 0
 0
 0
 1.74816639464111E-03
 5.86083298035376E-04
 5.34240305784008E-04
 0
 0
 4.77492401290604E-05

 0
 0
 0
 3.60442325984951E-05
 0
 0
 0
 0
 5.29152624720877E-05
 3.21460435202823E-05
 3.32248579833668E-05
 .177996652677581
 .109696515844071
 .21419980219175
 .114386816758536
 .143312350143716
 .242877181129776
 .201082708694403
 .48839231153925
 .25638753306763
 .157931566711333

 0
 0
 0
 3.60442325984951E-05
 0
 0
 0
 0
 5.29152624720877E-05
 3.21460435202823E-05
 3.32248579833668E-05
 .177996652677581
 .109696515844071
 .21419980219175
 .114386816758536
 .143312350143716
 .242877181129776
 .201082708694403
 .48839231153925
 .25638753306763
 .157931566711333

 0
 0
 0
 0
 0
 0
 0
 0
 0
 0
 0
 0
 8.00034879645145E-05
 0
 1.38354061341484E-04

 0
 0
 0
 0
 0
 0
 0
 0
 0
 0
 0
 0
 8.00034879645145E-05
 0
 1.38354061341484E-04

 5.61608587201003E-05
 1.12385835628801E-04
 1.34958859479175E-04
 1.56534952998855E-04
 0
 7.05162420697045E-05
 1.47225752691257E-04
 3.13286065245366E-05
 1.72352569194563E-04
 0
 1.80363514767221E-05
 0
 5.93710094893349E-04
 0
 4.48912906521239E-04
 1.16544426309295E-04
 7.99204497320689E-05
 2.33730133780984E-04
 4.09000421684906E-05
 0
 0

 5.61608587201003E-05
 1.12385835628801E-04
 1.34958859479175E-04
 1.56534952998855E-04
 0
 7.05162420697045E-05
 1.47225752691257E-04
 3.13286065245366E-05
 1.72352569194563E-04
 0
 1.80363514767221E-05
 0
 5.93710094893349E-04
 0
 4.48912906521239E-04
 1.16544426309295E-04
 7.99204497320689E-05
 2.33730133780984E-04
 4.09000421684906E-05
 0
 0

 5.61608587201003E-05
 1.12385835628801E-04
 1.34958859479175E-04
 1.56534952998855E-04
 0
 7.05162420697045E-05
 1.47225752691257E-04
 3.13286065245366E-05
 1.72352569194563E-04
 0
 1.80363514767221E-05
 0
 5.93710094893349E-04
 0
 4.48912906521239E-04
 1.16544426309295E-04
 7.99204497320689E-05
 2.33730133780984E-04
 4.09000421684906E-05
 0
 0

 1.69044184746957E-02
 1.88316515825403E-02
 2.34406669057726E-02
 2.45857710554021E-02
 9.17247802061362E-03
 1.07515232830763E-02
 .014063126585196
 1.01152238315944E-02
 1.85243105098945E-02
 7.49726099999211E-03
 1.02502839986016E-02
 3.2402303278681E-03
 2.5918272833023E-03
 2.81746472195157E-03
 2.39170774179812E-03
 3.66157613659123E-03
 3.87756896289377E-03
 5.43553736115289E-03
 4.34343840672164E-03
 0
 0

 1.17937803311886E-03
 3.93350424700803E-04
 7.08534012266156E-04
 3.69813826460107E-03
 9.4076697647663E-04
 4.93613694487932E-04
 2.57645067209345E-03
 6.57900737013457E-04
 1.80970197654042E-03
 2.44309930753641E-04
 5.05017841347697E-04
 3.08593364558944E-04
 1.79055425443735E-04
 4.84600008629314E-04
 1.8858254663366E-04
 5.06135794257511E-04
 1.4461795665814E-04
 2.17512124498817E-04
 8.88115201374216E-05
 0
 0

 1.17937803311886E-03
 3.93350424700803E-04
 7.08534012266156E-04
 3.69813826460107E-03
 9.4076697647663E-04
 4.93613694487932E-04
 2.57645067209345E-03
 6.57900737013457E-04
 1.80970197654042E-03
 2.44309930753641E-04
 5.05017841347697E-04
 3.08593364558944E-04
 1.79055425443735E-04
 4.84600008629314E-04
 1.8858254663366E-04
 5.06135794257511E-04
 1.4461795665814E-04
 2.17512124498817E-04
 8.88115201374216E-05
 0
 0

 3.68555635350151E-04
 1.22922007719255E-04
 4.42833757666348E-04
 0
 3.5278761617825E-04
 0
 0
 6.85313267724238E-05
 0
 0
 1.18363556565826E-04
 2.93163696330916E-03
 2.25609836059473E-03
 2.33286471332226E-03
 2.05256096347199E-03
 2.87864732984915E-03
 3.03697708981862E-03
 4.82151875970432E-03
 3.96321408613233E-03
 0
 0

 3.68555635350151E-04
 1.22922007719255E-04
 4.42833757666348E-04
 0
 3.5278761617825E-04
 0
 0
 6.85313267724238E-05
 0
 0
 1.18363556565826E-04
 2.93163696330916E-03
 2.25609836059473E-03
 2.33286471332226E-03
 2.05256096347199E-03
 2.87864732984915E-03
 3.03697708981862E-03
 4.82151875970432E-03
 3.96321408613233E-03
 0
 0

 1.53564848062267E-02
 1.83153791501202E-02
 2.22892991358401E-02
 .020887632790801
 7.87892342795874E-03
 1.02579095885883E-02
 1.14866759131025E-02
 9.38879176780848E-03
 1.67146085333541E-02
 7.25295106923847E-03
 9.62690260068804E-03
 0
 1.56673497263841E-04
 0
 1.50564231692471E-04
 2.76793012484576E-04
 6.95973916417009E-04
 3.96506476949753E-04
 2.91412800451892E-04
 0
 0

 1.53564848062267E-02
 1.83153791501202E-02
 2.22892991358401E-02
 .020887632790801
 7.87892342795874E-03
 1.02579095885883E-02
 1.14866759131025E-02
 9.38879176780848E-03
 1.67146085333541E-02
 7.25295106923847E-03
 9.62690260068804E-03
 0
 1.56673497263841E-04
 0
 1.50564231692471E-04
 2.76793012484576E-04
 6.95973916417009E-04
 3.96506476949753E-04
 2.91412800451892E-04
 0
 0

 2.30259520752086E-03
 1.6857875344334E-03
 1.82194460296872E-03
 7.67021269694779E-03
 3.54803545413377E-03
 1.0577436310463E-03
 4.3676973298434E-03
 1.00251540878289E-02
 9.19213702370628E-03
 5.89833975676828E-03
 6.90792261556728E-03
 2.20423831828143E-03
 1.07791366117411E-02
 1.96916539080877E-03
 9.35176166233625E-03
 4.04908635406737E-03
 4.59885102172634E-03
 3.36555922364289E-03
 1.93045040730931E-03
 1.28944245703419E-04
 7.61517418056879E-04

 2.02179091392036E-03
 1.51720878099009E-03
 1.55202688401037E-03
 6.41793307295727E-03
 1.88153395294882E-03
 8.46194904837328E-04
 3.72971906818284E-03
 5.48250614178097E-03
 8.04311989574309E-03
 3.07132484375891E-03
 2.20043488015354E-03
 0
 0
 0
 0
 0
 0
 1.02877356181479E-04
 2.10027243568529E-05
 4.14041155929216E-05
 7.35596401986818E-04

 2.02179091392036E-03
 1.51720878099009E-03
 1.55202688401037E-03
 6.41793307295727E-03
 1.88153395294882E-03
 8.46194904837328E-04
 3.72971906818284E-03
 5.48250614178097E-03
 8.04311989574309E-03
 3.07132484375891E-03
 2.20043488015354E-03
 0
 0
 0
 0
 0
 0
 1.02877356181479E-04
 2.10027243568529E-05
 4.14041155929216E-05
 7.35596401986818E-04

 2.02179091392036E-03
 1.51720878099009E-03
 1.55202688401037E-03
 6.41793307295727E-03
 1.88153395294882E-03
 8.46194904837328E-04
 3.72971906818284E-03
 5.48250614178097E-03
 8.04311989574309E-03
 3.07132484375891E-03
 2.20043488015354E-03
 0
 0
 0
 0
 0
 0
 1.02877356181479E-04
 2.10027243568529E-05
 4.14041155929216E-05
 7.35596401986818E-04

 2.80804293600502E-04
 1.68578753443317E-04
 2.6991771895835E-04
 1.25227962399052E-03
 1.66650150118495E-03
 2.11548726208968E-04
 6.37978261660561E-04
 4.54264794604797E-03
 1.1490171279632E-03
 2.82701491300937E-03
 4.70748773541374E-03
 2.20423831828143E-03
 1.07791366117411E-02
 1.96916539080877E-03
 9.35176166233625E-03
 4.04908635406737E-03
 4.59885102172634E-03
 3.26268186746141E-03
 1.90944768295246E-03
 8.75401301104969E-05
 2.59210160700614E-05

 0
 0
 0
 1.0174771944924E-03
 1.66650150118495E-03
 0
 3.43526756278452E-04
 4.54264794604797E-03
 9.76664558768634E-04
 2.82701491300937E-03
 4.70748773541374E-03
 2.16015355191583E-03
 7.5919500388395E-03
 1.86454717603832E-03
 6.70158348082873E-03

 0
 0
 0
 1.0174771944924E-03
 1.61274338824343E-03
 0
 3.43526756278452E-04
 4.51131933952344E-03
 9.76664558768634E-04
 2.79211349433021E-03
 4.70748773541374E-03
 2.16015355191583E-03
 7.5919500388395E-03
 1.86454717603832E-03
 6.70158348082873E-03

 0
 0
 0
 0
 5.37581129415217E-05
 0
 0
 3.13286065245366E-05
 0
 3.49014186791637E-05
 0

 2.80804293600502E-04
 1.68578753443317E-04
 2.6991771895835E-04
 2.34802429498121E-04
 0
 2.11548726208968E-04
 2.94451505382109E-04
 0
 1.72352569194563E-04
 0
 0
 4.40847663655979E-05
 3.18718657290162E-03
 1.04618214770448E-04
 2.65017818150752E-03
 4.04908635406737E-03
 4.59885102172634E-03
 3.26268186746141E-03
 1.90944768295246E-03
 8.75401301104969E-05
 2.59210160700614E-05

 2.80804293600502E-04
 1.68578753443317E-04
 2.6991771895835E-04
 2.34802429498121E-04
 0
 2.11548726208968E-04
 2.94451505382109E-04
 0
 1.72352569194563E-04
 0
 0
 4.40847663655979E-05
 3.18718657290162E-03
 1.04618214770448E-04
 2.65017818150752E-03
 4.04908635406737E-03
 4.59885102172634E-03
 3.26268186746141E-03
 1.90944768295246E-03
 8.75401301104969E-05
 2.59210160700614E-05

 0
 0
 0
 0
 0
 0
 0
 0
 0
 0
 0
 2.02514395491101E-02
 2.02108811470555E-02
 1.80736435648564E-02
 1.87005110577318E-02
 .105042948238161
 .114329533360453
 .111656223908967
 .013939245621569
 1.72344631155316E-03
 2.55160001939178E-03

 0
 0
 0
 0
 0
 0
 0
 0
 0
 0
 0
 2.02514395491101E-02
 2.02108811470555E-02
 1.80736435648564E-02
 1.87005110577318E-02
 .105042948238161
 .114329533360453
 .111656223908967
 .013939245621569
 1.72344631155316E-03
 2.55160001939178E-03

 0
 0
 0
 0
 0
 0
 0
 0
 0
 0
 0
 2.02514395491101E-02
 2.02108811470555E-02
 1.80736435648564E-02
 1.87005110577318E-02
 .105042948238161
 .114329533360453
 .111656223908967
 .013939245621569
 1.72344631155316E-03
 2.55160001939178E-03

 0
 0
 0
 0
 0
 0
 0
 0
 0
 0
 0
 2.02514395491101E-02
 2.02108811470555E-02
 1.80736435648564E-02
 1.87005110577318E-02
 .105042948238161
 .114329533360453
 .111656223908967
 .013939245621569
 1.72344631155316E-03
 2.55160001939178E-03

 8.36438321363196E-05
 1.6738315944715E-04
 0
 1.16568582020424E-04
 8.00652745937557E-05
 0
 1.46181598416751E-04
 1.39978880215822E-04
 0
 0
 2.68626511355436E-05
 6.56581626721671E-05
 1.60006975929029E-04
 3.28290813360836E-05
 1.29179646644038E-04
 0
 4.30776892173679E-05
 5.39923713293901E-05
 2.6454495360045E-04
 1.95568375778957E-04
 1.54423074459718E-04

 8.36438321363196E-05
 1.6738315944715E-04
 0
 1.16568582020424E-04
 8.00652745937557E-05
 0
 1.46181598416751E-04
 1.39978880215822E-04
 0
 0
 2.68626511355436E-05
 6.56581626721671E-05
 1.60006975929029E-04
 3.28290813360836E-05
 1.29179646644038E-04
 0
 4.30776892173679E-05
 5.39923713293901E-05
 2.6454495360045E-04
 1.95568375778957E-04
 1.54423074459718E-04

 8.36438321363196E-05
 1.6738315944715E-04
 0
 1.16568582020424E-04
 8.00652745937557E-05
 0
 1.46181598416751E-04
 1.39978880215822E-04
 0
 0
 2.68626511355436E-05
 6.56581626721671E-05
 1.60006975929029E-04
 3.28290813360836E-05
 1.29179646644038E-04
 0
 4.30776892173679E-05
 5.39923713293901E-05
 2.6454495360045E-04
 1.95568375778957E-04
 1.54423074459718E-04

 8.36438321363196E-05
 1.6738315944715E-04
 0
 1.16568582020424E-04
 8.00652745937557E-05
 0
 1.46181598416751E-04
 1.39978880215822E-04
 0
 0
 2.68626511355436E-05
 6.56581626721671E-05
 1.60006975929029E-04
 3.28290813360836E-05
 1.29179646644038E-04
 0
 4.30776892173679E-05
 5.39923713293901E-05
 2.6454495360045E-04
 1.95568375778957E-04
 1.54423074459718E-04

 8.36438321363196E-05
 1.6738315944715E-04
 0
 1.16568582020424E-04
 8.00652745937557E-05
 0
 1.46181598416751E-04
 1.39978880215822E-04
 0
 0
 2.68626511355436E-05
 6.56581626721671E-05
 1.60006975929029E-04
 3.28290813360836E-05
 1.29179646644038E-04
 0
 4.30776892173679E-05
 5.39923713293901E-05
 2.6454495360045E-04
 1.95568375778957E-04
 1.54423074459718E-04

 7.52794489226185E-04
 4.18457898618048E-04
 7.03508948350344E-04
 6.41127201111971E-04
 7.2058747134314E-04
 4.20096761266759E-04
 3.65453996042027E-04
 5.03923968777421E-03
 1.71130210547675E-04
 1.40348258092614E-03
 6.17840976116281E-04
 6.56581626721671E-05
 4.26685269142907E-04
 3.28290813360836E-05
 5.57575745327759E-04
 7.16128304640405E-03
 9.86479083076066E-03
 8.69277178402467E-03
 4.62953668802309E-04
 1.23859971327023E-03
 2.23913457966602E-03

 4.18219160681598E-04
 0
 1.00501278335763E-04
 1.16568582020424E-04
 8.00652745937557E-05
 4.20096761266759E-04
 1.46181598416751E-04
 2.33298133693358E-04
 0
 1.55942508991793E-04
 2.95489162490535E-04
 0
 5.33356586429122E-05
 0
 7.58439880009797E-05
 7.53819268043102E-04
 7.75398405911632E-04
 8.09885569939299E-04
 4.29885549602157E-04
 8.47462961710814E-04
 1.96889419936135E-03

 4.18219160681598E-04
 0
 1.00501278335763E-04
 1.16568582020424E-04
 8.00652745937557E-05
 4.20096761266759E-04
 1.46181598416751E-04
 2.33298133693358E-04
 0
 1.55942508991793E-04
 2.95489162490535E-04
 0
 5.33356586429122E-05
 0
 7.58439880009797E-05
 7.53819268043102E-04
 7.75398405911632E-04
 8.09885569939299E-04
 4.29885549602157E-04
 8.47462961710814E-04
 1.96889419936135E-03

 4.18219160681598E-04
 0
 1.00501278335763E-04
 1.16568582020424E-04
 8.00652745937557E-05
 4.20096761266759E-04
 1.46181598416751E-04
 2.33298133693358E-04
 0
 1.55942508991793E-04
 2.95489162490535E-04
 0
 5.33356586429122E-05
 0
 7.58439880009797E-05
 7.53819268043102E-04
 7.75398405911632E-04
 8.09885569939299E-04
 4.29885549602157E-04
 8.47462961710814E-04
 1.96889419936135E-03

 4.18219160681598E-04
 0
 1.00501278335763E-04
 1.16568582020424E-04
 8.00652745937557E-05
 4.20096761266759E-04
 1.46181598416751E-04
 2.33298133693358E-04
 0
 1.55942508991793E-04
 2.95489162490535E-04
 0
 5.33356586429122E-05
 0
 7.58439880009797E-05
 7.53819268043102E-04
 7.75398405911632E-04
 8.09885569939299E-04
 4.29885549602157E-04
 8.47462961710814E-04
 1.96889419936135E-03

 0
 8.36915797237479E-05
 0
 0
 0
 0
 0
 0
 0
 5.19808363306693E-05
 5.37253022709762E-05

 0
 8.36915797237479E-05
 0
 0
 0
 0
 0
 0
 0
 5.19808363306693E-05
 5.37253022709762E-05

 0
 8.36915797237479E-05
 0
 0
 0
 0
 0
 0
 0
 5.19808363306693E-05
 5.37253022709762E-05

 0
 8.36915797237479E-05
 0
 0
 0
 0
 0
 0
 0
 5.19808363306693E-05
 5.37253022709762E-05

 3.34575328544587E-04
 3.347663188943E-04
 6.0300767001458E-04
 5.24558619091547E-04
 6.40522196749384E-04
 0
 2.19272397625277E-04
 4.80594155408086E-03
 1.71130210547675E-04
 1.19555923560368E-03
 2.6862651135477E-04
 6.56581626721671E-05
 3.73349610499995E-04
 3.28290813360836E-05
 4.8173175732678E-04
 6.40746377836095E-03
 9.08939242484903E-03
 7.88288621408537E-03
 3.30681192001513E-05
 3.91136751559414E-04
 2.70240380304673E-04

 3.34575328544587E-04
 3.347663188943E-04
 6.0300767001458E-04
 5.24558619091547E-04
 6.40522196749384E-04
 0
 2.19272397625277E-04
 4.80594155408086E-03
 1.71130210547675E-04
 1.19555923560368E-03
 2.6862651135477E-04
 6.56581626721671E-05
 3.73349610499995E-04
 3.28290813360836E-05
 4.8173175732678E-04
 6.40746377836095E-03
 9.08939242484903E-03
 7.88288621408537E-03
 3.30681192001513E-05
 3.91136751559414E-04
 2.70240380304673E-04

 3.34575328544587E-04
 3.347663188943E-04
 6.0300767001458E-04
 5.24558619091547E-04
 6.40522196749384E-04
 0
 2.19272397625277E-04
 4.80594155408086E-03
 1.71130210547675E-04
 1.19555923560368E-03
 2.6862651135477E-04
 6.56581626721671E-05
 3.73349610499995E-04
 3.28290813360836E-05
 4.8173175732678E-04
 6.40746377836095E-03
 9.08939242484903E-03
 7.88288621408537E-03
 3.30681192001513E-05
 3.91136751559414E-04
 2.70240380304673E-04

 1.67287664272294E-04
 1.6738315944715E-04
 1.00501278335763E-04
 0
 0
 0
 7.30907992085262E-05
 0
 0
 5.19808363306693E-05
 2.68626511355436E-05

 1.67287664272294E-04
 1.6738315944715E-04
 5.02506391678817E-04
 5.24558619091547E-04
 6.40522196749384E-04
 0
 1.46181598416751E-04
 4.80594155408086E-03
 1.71130210547675E-04
 1.14357839927301E-03
 2.41763860219226E-04
 0
 3.20013951857083E-04
 0
 4.55063928005324E-04
 6.40746377836095E-03
 9.08939242484903E-03
 7.88288621408537E-03
 3.30681192001513E-05
 3.91136751559414E-04
 2.70240380304673E-04

 0
 0
 0
 0
 0
 0
 0
 0
 0
 0
 0
 6.56581626721671E-05
 5.33356586429122E-05
 3.28290813360836E-05
 2.66678293214561E-05

 6.3882976793972E-03
 7.37532046314513E-03
 5.60922759710748E-03
 1.24983376535338E-02
 0
 4.01061126771381E-03
 6.4411266802425E-03
 2.74125307089129E-04
 1.13106373534295E-02
 0
 0
 5.20751302694931E-03
 1.56673497263554E-04
 4.41010282945118E-03
 7.83367486317772E-05
 6.64303229964575E-03
 8.22514628490845E-03
 7.77152694820858E-03
 1.02965856159482E-02
 2.29792841540054E-03
 2.60830224204406E-03

 6.3882976793972E-03
 7.37532046314513E-03
 5.60922759710748E-03
 1.24983376535338E-02
 0
 4.01061126771381E-03
 6.4411266802425E-03
 2.74125307089129E-04
 1.13106373534295E-02
 0
 0
 5.20751302694931E-03
 1.56673497263554E-04
 4.41010282945118E-03
 7.83367486317772E-05
 6.64303229964575E-03
 8.22514628490845E-03
 7.77152694820858E-03
 1.02965856159482E-02
 2.29792841540054E-03
 2.60830224204406E-03

 6.3882976793972E-03
 7.37532046314513E-03
 5.60922759710748E-03
 1.24983376535338E-02
 0
 4.01061126771381E-03
 6.4411266802425E-03
 2.74125307089129E-04
 1.13106373534295E-02
 0
 0
 5.20751302694931E-03
 1.56673497263554E-04
 4.41010282945118E-03
 7.83367486317772E-05
 6.64303229964575E-03
 8.22514628490845E-03
 7.77152694820858E-03
 1.02965856159482E-02
 2.29792841540054E-03
 2.60830224204406E-03

 6.3882976793972E-03
 7.37532046314513E-03
 5.60922759710748E-03
 1.24983376535338E-02
 0
 4.01061126771381E-03
 6.4411266802425E-03
 2.74125307089129E-04
 1.13106373534295E-02
 0
 0
 5.20751302694931E-03
 1.56673497263554E-04
 4.41010282945118E-03
 7.83367486317772E-05
 6.64303229964575E-03
 8.22514628490845E-03
 7.77152694820858E-03
 1.02965856159482E-02
 2.29792841540054E-03
 2.60830224204406E-03

 6.3882976793972E-03
 7.37532046314513E-03
 5.60922759710748E-03
 1.24983376535338E-02
 0
 4.01061126771381E-03
 6.4411266802425E-03
 2.74125307089129E-04
 1.13106373534295E-02
 0
 0
 5.20751302694931E-03
 1.56673497263554E-04
 4.41010282945118E-03
 7.83367486317772E-05
 6.64303229964575E-03
 8.22514628490845E-03
 7.77152694820858E-03
 1.02965856159482E-02
 2.29792841540054E-03
 2.60830224204406E-03

 1.50558897844961E-03
 1.50644843502331E-03
 1.60802045337055E-03
 7.34382066727685E-03
 2.16176241402876E-03
 2.10048380633162E-04
 5.04326514536536E-03
 3.91940864604147E-03
 3.08034378985956E-03
 2.54706098020365E-03
 3.86822176350984E-03
 3.28290813360235E-03
 3.62682478771881E-03
 3.36324800202627E-03
 4.71480575595391E-03
 6.59591859539069E-03
 7.23705178850691E-03
 7.23497775813144E-03
 4.19965113842093E-03
 1.21904287568851E-02
 4.7099037710224E-03

 1.50558897844961E-03
 1.50644843502331E-03
 1.60802045337055E-03
 7.34382066727685E-03
 2.16176241402876E-03
 2.10048380633162E-04
 5.04326514536536E-03
 3.91940864604147E-03
 3.08034378985956E-03
 2.54706098020365E-03
 3.86822176350984E-03
 3.28290813360235E-03
 3.62682478771881E-03
 3.36324800202627E-03
 4.71480575595391E-03
 6.59591859539069E-03
 7.23705178850691E-03
 7.23497775813144E-03
 4.19965113842093E-03
 1.21904287568851E-02
 4.7099037710224E-03

 1.50558897844961E-03
 1.50644843502331E-03
 1.60802045337055E-03
 7.34382066727685E-03
 2.16176241402876E-03
 2.10048380633162E-04
 5.04326514536536E-03
 3.91940864604147E-03
 3.08034378985956E-03
 2.54706098020365E-03
 3.86822176350984E-03
 3.28290813360235E-03
 3.62682478771881E-03
 3.36324800202627E-03
 4.71480575595391E-03
 6.59591859539069E-03
 7.23705178850691E-03
 7.23497775813144E-03
 4.19965113842093E-03
 1.21904287568851E-02
 4.7099037710224E-03

 1.50558897844961E-03
 1.50644843502331E-03
 1.60802045337055E-03
 7.34382066727685E-03
 2.16176241402876E-03
 2.10048380633162E-04
 5.04326514536536E-03
 3.91940864604147E-03
 3.08034378985956E-03
 2.54706098020365E-03
 3.86822176350984E-03
 3.28290813360235E-03
 3.62682478771881E-03
 3.36324800202627E-03
 4.71480575595391E-03
 6.59591859539069E-03
 7.23705178850691E-03
 7.23497775813144E-03
 4.19965113842093E-03
 1.21904287568851E-02
 4.7099037710224E-03

 1.50558897844961E-03
 1.50644843502331E-03
 1.60802045337055E-03
 7.34382066727685E-03
 2.16176241402876E-03
 2.10048380633162E-04
 5.04326514536536E-03
 3.91940864604147E-03
 3.08034378985956E-03
 2.54706098020365E-03
 3.86822176350984E-03
 3.28290813360235E-03
 3.62682478771881E-03
 3.36324800202627E-03
 4.71480575595391E-03
 6.59591859539069E-03
 7.23705178850691E-03
 7.23497775813144E-03
 4.19965113842093E-03
 1.21904287568851E-02
 4.7099037710224E-03

 2.06908426863527E-04
 0
 0
 4.32530791181345E-04
 0
 0
 0
 0
 0
 0
 0
 4.87252680883816E-04
 1.31935576642993E-03
 2.43626340441908E-04
 1.14626303225535E-03
 4.66177705237181E-04
 2.13121199285313E-04
 6.67800382231162E-04
 8.18000843372165E-05
 0
 0

 2.06908426863527E-04
 0
 0
 4.32530791181345E-04
 0
 0
 0
 0
 0
 0
 0
 4.87252680883816E-04
 1.31935576642993E-03
 2.43626340441908E-04
 1.14626303225535E-03
 4.66177705237181E-04
 2.13121199285313E-04
 6.67800382231162E-04
 8.18000843372165E-05
 0
 0

 2.06908426863527E-04
 0
 0
 4.32530791181345E-04
 0
 0
 0
 0
 0
 0
 0
 4.87252680883816E-04
 1.31935576642993E-03
 2.43626340441908E-04
 1.14626303225535E-03
 4.66177705237181E-04
 2.13121199285313E-04
 6.67800382231162E-04
 8.18000843372165E-05
 0
 0

 2.06908426863527E-04
 0
 0
 4.32530791181345E-04
 0
 0
 0
 0
 0
 0
 0
 4.87252680883816E-04
 1.31935576642993E-03
 2.43626340441908E-04
 1.14626303225535E-03
 4.66177705237181E-04
 2.13121199285313E-04
 6.67800382231162E-04
 8.18000843372165E-05
 0
 0

 2.06908426863527E-04
 0
 0
 4.32530791181345E-04
 0
 0
 0
 0
 0
 0
 0
 0
 2.63871153286469E-04
 0
 1.31935576643235E-04

 0
 0
 0
 0
 0
 0
 0
 0
 0
 0
 0
 4.87252680883816E-04
 1.05548461314346E-03
 2.43626340441908E-04
 1.01432745561212E-03
 4.66177705237181E-04
 2.13121199285313E-04
 6.67800382231162E-04
 8.18000843372165E-05
 0
 0

 .102019830299801
 .133188372363875
 .142218825641288
 .192225840490388
 3.33208788919436E-02
 7.23757257385104E-02
 .175131165679739
 .047447843124648
 .100324813069327
 6.09537392610761E-02
 5.36956522947475E-02
 .10117015913665
 .1038064809308
 9.01325029176955E-02
 .100825382786136
 .02931494517985
 4.17780321555341E-02
 2.95523704770384E-02
 .13381730898835
 .100165357327093
 .245324176876398

 2.54756000575209E-03
 2.25791938787004E-03
 5.90445010221391E-04
 1.07084138301572E-03
 0
 2.46196799779664E-03
 1.32125675492336E-04
 5.22946739677898E-04
 0
 0
 0
 7.34746106093299E-05
 4.50103788427293E-03
 3.67373053046649E-05
 3.22527893601572E-03
 1.38396506242686E-04
 8.02534516059972E-04
 6.50270622197157E-04
 3.03887313312265E-03
 8.6172315577592E-04
 9.61669696197164E-03

 8.276337074524E-04
 1.55906909223591E-03
 0
 8.21808503244556E-04
 0
 1.13411243571747E-03
 1.32125675492336E-04
 5.22946739677898E-04
 0
 0
 0
 0
 3.70927449762211E-03
 0
 2.56018571491877E-03
 0
 0
 0
 7.77100801201769E-04
 6.12780910773478E-04
 1.45157689991926E-03

 8.276337074524E-04
 6.21079617949064E-04
 0
 0
 0
 1.03918672523882E-03
 0
 0
 0
 0
 0
 0
 2.84980845548904E-03
 0
 1.89932474805847E-03
 0
 0
 0
 4.6626048072124E-04
 6.12780910773478E-04
 0

 8.276337074524E-04
 6.21079617949064E-04
 0
 0
 0
 1.03918672523882E-03
 0
 0
 0
 0
 0
 0
 1.84709807300046E-03
 0
 1.16684161102042E-03

 0
 0
 0
 0
 0
 0
 0
 0
 0
 0
 0
 0
 1.00271038248858E-03
 0
 7.32483137038052E-04
 0
 0
 0
 4.6626048072124E-04
 6.12780910773478E-04
 0

 0
 1.51288624885237E-04
 0
 0
 0
 9.49257104786444E-05
 1.32125675492336E-04
 8.43462483352908E-05
 0
 0
 0

 0
 1.51288624885237E-04
 0
 0
 0
 9.49257104786444E-05
 1.32125675492336E-04
 8.43462483352908E-05
 0
 0
 0

 0
 7.86700849401606E-04
 0
 8.21808503244556E-04
 0
 0
 0
 4.38600491342607E-04
 0
 0
 0
 0
 5.01355191244291E-04
 0
 4.81805541415907E-04
 0
 0
 0
 3.10840320480529E-04
 0
 1.45157689991926E-03

 0
 7.86700849401606E-04
 0
 8.21808503244556E-04
 0
 0
 0
 4.38600491342607E-04
 0
 0
 0
 0
 5.01355191244291E-04
 0
 4.81805541415907E-04
 0
 0
 0
 3.10840320480529E-04
 0
 1.45157689991926E-03

 0
 0
 0
 0
 0
 0
 0
 0
 0
 0
 0
 0
 3.58110850888779E-04
 0
 1.7905542544439E-04

 0
 0
 0
 0
 0
 0
 0
 0
 0
 0
 0
 0
 3.58110850888779E-04
 0
 1.7905542544439E-04

 0
 0
 0
 0
 0
 0
 0
 0
 0
 0
 0
 0
 0
 0
 0

 0
 0
 0
 0
 0
 0
 0
 0
 0
 0
 0
 0
 0
 0
 0

 7.37111270699287E-04
 3.68766023157256E-04
 5.90445010221391E-04
 0
 0
 1.15690709645529E-03
 0
 0
 0
 0
 0
 0
 1.56673497263841E-04
 0
 2.95019197813968E-04
 1.38396506242686E-04
 6.95973916417009E-04
 3.96506476949753E-04
 7.77100801201322E-04
 9.57470173086313E-05
 8.16512006205238E-03

 7.37111270699287E-04
 3.68766023157256E-04
 5.90445010221391E-04
 0
 0
 1.15690709645529E-03
 0
 0
 0
 0
 0
 0
 1.56673497263841E-04
 0
 2.95019197813968E-04
 1.38396506242686E-04
 6.95973916417009E-04
 3.96506476949753E-04
 7.77100801201322E-04
 9.57470173086313E-05
 8.16512006205238E-03

 7.37111270699287E-04
 3.68766023157256E-04
 5.90445010221391E-04
 0
 0
 1.15690709645529E-03
 0
 0
 0
 0
 0
 0
 1.56673497263841E-04
 0
 2.95019197813968E-04
 1.38396506242686E-04
 6.95973916417009E-04
 3.96506476949753E-04
 7.77100801201322E-04
 9.57470173086313E-05
 8.16512006205238E-03

 0
 0
 0
 1.24516439885453E-04
 0
 5.87635350582085E-05
 0
 0
 0
 0
 0
 7.34746106093299E-05
 1.1394436164643E-04
 3.67373053046649E-05
 1.09501259412706E-04

 0
 0
 0
 0
 0
 5.87635350582085E-05
 0
 0
 0
 0
 0

 0
 0
 0
 0
 0
 5.87635350582085E-05
 0
 0
 0
 0
 0

 0
 0
 0
 1.24516439885453E-04
 0
 0
 0
 0
 0
 0
 0
 0
 1.1394436164643E-04
 0
 1.09501259412706E-04

 0
 0
 0
 1.24516439885453E-04
 0
 0
 0
 0
 0
 0
 0

 0
 0
 0
 0
 0
 0
 0
 0
 0
 0
 0
 0
 1.1394436164643E-04
 0
 1.09501259412706E-04

 0
 0
 0
 0
 0
 0
 0
 0
 0
 0
 0
 7.34746106093299E-05
 0
 3.67373053046649E-05
 0

 0
 0
 0
 0
 0
 0
 0
 0
 0
 0
 0
 7.34746106093299E-05
 0
 3.67373053046649E-05
 0

 9.82815027600402E-04
 3.3008427247688E-04
 0
 1.2451643988571E-04
 0
 1.12184930565671E-04
 0
 0
 0
 0
 0
 0
 1.25338797810844E-04
 0
 6.26693989054218E-05

 9.82815027600402E-04
 3.3008427247688E-04
 0
 1.2451643988571E-04
 0
 1.12184930565671E-04
 0
 0
 0
 0
 0
 0
 1.25338797810844E-04
 0
 6.26693989054218E-05

 0
 1.51288624885237E-04
 0
 0
 0
 0
 0
 0
 0
 0
 0

 0
 1.78795647591643E-04
 0
 1.2451643988571E-04
 0
 1.12184930565671E-04
 0
 0
 0
 0
 0

 9.82815027600402E-04
 0
 0
 0
 0
 0
 0
 0
 0
 0
 0

 0
 0
 0
 0
 0
 0
 0
 0
 0
 0
 0
 0
 1.25338797810844E-04
 0
 6.26693989054218E-05

 0
 0
 0
 0
 0
 0
 0
 0
 0
 0
 0
 0
 3.95806729929704E-04
 0
 1.97903364964852E-04
 0
 1.06560599642963E-04
 2.53764145247404E-04
 1.48467153071956E-03
 1.5319522769381E-04
 0

 0
 0
 0
 0
 0
 0
 0
 0
 0
 0
 0
 0
 3.95806729929704E-04
 0
 1.97903364964852E-04
 0
 1.06560599642963E-04
 2.53764145247404E-04
 1.48467153071956E-03
 1.5319522769381E-04
 0

 0
 0
 0
 0
 0
 0
 0
 0
 0
 0
 0
 0
 3.95806729929704E-04
 0
 1.97903364964852E-04
 0
 0
 2.53764145247404E-04
 1.3210713620456E-03
 1.5319522769381E-04
 0

 0
 0
 0
 0
 0
 0
 0
 0
 0
 0
 0
 0
 0
 0
 0
 0
 1.06560599642963E-04
 0
 1.63600168673962E-04
 0
 0

 1.35167749367486E-02
 8.59288252209426E-03
 5.62726691969221E-02
 3.47314375885792E-03
 3.44947891374764E-04
 .021847451260808
 4.73872014867784E-04
 5.48250614179391E-05
 8.30732313012884E-03
 2.70776839918404E-03
 1.08368411789071E-03
 1.01142537351762E-03
 3.63115438824465E-02
 1.14921508398048E-03
 3.58755811187457E-02
 1.45956408178803E-02
 1.13995475383104E-02
 1.03089328457381E-02
 3.39412148061328E-02
 1.88589269035597E-03
 .103210137918177

 1.09201669733528E-04
 1.0926400686156E-04
 2.6242000454284E-04
 0
 0
 1.02836186351582E-03
 0
 0
 0
 0
 0

 1.09201669733528E-04
 1.0926400686156E-04
 2.6242000454284E-04
 0
 0
 1.02836186351582E-03
 0
 0
 0
 0
 0

 1.09201669733528E-04
 1.0926400686156E-04
 2.6242000454284E-04
 0
 0
 1.02836186351582E-03
 0
 0
 0
 0
 0

 9.05042664465527E-03
 6.05795756291094E-03
 3.89267068966237E-02
 1.60124661258249E-03
 1.25435596863551E-04
 1.73637935358927E-02
 3.02108636728102E-04
 5.48250614179391E-05
 1.57121021743263E-03
 5.70056505090315E-04
 8.41696402245293E-05
 7.28548122671328E-04
 3.56012906948521E-02
 7.18761048000732E-04
 3.52893265791543E-02
 .012824165537979
 1.07246637405716E-02
 9.63222845841171E-03
 3.34231476053318E-02
 1.88589269035597E-03
 9.61034593456566E-02

 0
 0
 7.50212483575414E-04
 0
 0
 0
 0
 0
 0
 0
 0
 0
 4.59575591973781E-04
 0
 7.69086336173319E-04
 0
 9.78581506717133E-04
 2.11470121040111E-04
 7.77100801203556E-05
 1.02130151795873E-04
 2.88180708072207E-04

 0
 0
 4.72356008177112E-04
 0
 0
 0
 0
 0
 0
 0
 0
 0
 8.35591985405624E-05
 0
 3.49950193662526E-04
 0
 9.11093126943257E-04
 1.26882072624067E-04
 7.77100801203556E-05
 0
 1.81447112489908E-04

 0
 0
 2.77856475398301E-04
 0
 0
 0
 0
 0
 0
 0
 0
 0
 3.76016393433218E-04
 0
 4.19136142510793E-04
 0
 0
 0
 0
 0
 1.06733595582299E-04

 0
 0
 0
 0
 0
 0
 0
 0
 0
 0
 0
 0
 0
 0
 0

 0
 0
 0
 0
 0
 0
 0
 0
 0
 0
 0
 0
 0
 0
 0
 0
 6.74883797738765E-05
 8.45880484160445E-05
 0
 1.02130151795873E-04
 0

 9.05042664465527E-03
 6.05795756291094E-03
 3.81764944130483E-02
 1.53276257064534E-03
 1.25435596863551E-04
 1.73637935358927E-02
 3.02108636728102E-04
 0
 1.57121021743263E-03
 5.70056505090315E-04
 8.41696402245293E-05
 7.28548122671328E-04
 3.50679863982837E-02
 7.18761048000732E-04
 3.44833758906837E-02
 .012824165537979
 9.74608223385448E-03
 9.4207583373716E-03
 3.33454375252114E-02
 1.7837625385601E-03
 9.58152786375844E-02

 2.62084007359927E-04
 0
 0
 1.82624111831998E-04
 0
 0
 0
 0
 0
 0
 0

 6.5521001840117E-04
 5.24467232934404E-04
 1.57452002726029E-04
 5.47872335495616E-04
 1.25435596863551E-04
 1.64537898162644E-04
 2.29017837519576E-04
 0
 6.70259991313111E-04
 5.70056505090315E-04
 8.41696402245293E-05
 1.02864454853062E-04
 5.84914389783478E-04
 5.14322274265309E-05
 1.06288368087094E-03
 7.38114699960144E-03
 4.92665172348949E-03
 6.17492753436687E-03
 7.40836097146824E-03
 5.1065075897878E-04
 6.77402553297743E-02

 4.43312310322079E-03
 3.6824295078532E-03
 4.12055241176049E-03
 6.41127201112694E-04
 0
 1.19727576960907E-02
 7.30907992085262E-05
 0
 4.27825526370071E-04
 0
 0
 2.62632650689149E-04
 1.41339495403927E-02
 3.15794246976557E-04
 1.40008131440066E-02
 7.53819268043102E-04
 1.68002987947562E-03
 1.40380165456166E-03
 8.20089356162156E-03
 9.12652420302799E-04
 1.99205766053039E-02

 3.70000951567338E-03
 1.85106082212334E-03
 3.38984899985618E-02
 1.61138922205037E-04
 0
 5.22649794163932E-03
 0
 0
 4.73124699749454E-04
 0
 0
 3.63051017129117E-04
 2.03491224681075E-02
 3.51534573597644E-04
 1.94196790658062E-02
 4.68919927033443E-03
 2.9774285194323E-03
 1.79127631939344E-03
 1.77361829921217E-02
 3.60459359278517E-04
 7.79155247752469E-03

 0
 0
 0
 0
 0
 0
 0
 0
 0
 0
 0
 0
 0
 0
 0
 0
 1.61972111457071E-04
 5.07528290496267E-05
 0
 0
 0

 0
 0
 0
 0
 0
 0
 0
 0
 0
 0
 0
 0
 0
 0
 0
 0
 0
 0
 0
 0
 3.62894224981555E-04

 0
 0
 0
 6.84840419371406E-05
 0
 0
 0
 5.48250614179391E-05
 0
 0
 0

 0
 0
 0
 6.84840419371406E-05
 0
 0
 0
 5.48250614179391E-05
 0
 0
 0

 0
 0
 0
 0
 0
 0
 0
 0
 0
 0
 0
 0
 7.37287045946139E-05
 0
 3.68643522973069E-05

 0
 0
 0
 0
 0
 0
 0
 0
 0
 0
 0
 0
 7.37287045946139E-05
 0
 3.68643522973069E-05

 2.94844508280121E-04
 5.90025637050798E-04
 2.36178004088556E-04
 1.23271275486514E-03
 9.4076697647663E-05
 0
 1.71763378139682E-04
 0
 4.72533293876241E-03
 1.40478210183331E-03
 6.62835916767712E-04
 7.71483411397964E-05
 1.25338797811073E-04
 3.85741705698982E-05
 6.26693989055364E-05

 2.94844508280121E-04
 5.90025637050798E-04
 2.36178004088556E-04
 1.23271275486514E-03
 9.4076697647663E-05
 0
 1.71763378139682E-04
 0
 4.72533293876241E-03
 1.40478210183331E-03
 6.62835916767712E-04
 7.71483411397964E-05
 1.25338797811073E-04
 3.85741705698982E-05
 6.26693989055364E-05

 2.94844508280121E-04
 5.90025637050798E-04
 2.36178004088556E-04
 1.23271275486514E-03
 9.4076697647663E-05
 0
 1.71763378139682E-04
 0
 4.72533293876241E-03
 1.40478210183331E-03
 6.62835916767712E-04
 7.71483411397964E-05
 1.25338797811073E-04
 3.85741705698982E-05
 6.26693989055364E-05

 4.06230211407968E-03
 1.83563531527095E-03
 1.68473642916669E-02
 6.39184391410295E-04
 1.25435596863551E-04
 3.45529586139955E-03
 0
 0
 2.01077997393379E-03
 7.32929792260419E-04
 3.36678560898465E-04
 2.057289097065E-04
 5.84914389783325E-04
 3.91879865409847E-04
 5.23585140685847E-04
 1.77147527990129E-03
 6.74883797738764E-04
 6.76704387326411E-04
 5.1806720080103E-04
 0
 7.10667857252009E-03

 4.06230211407968E-03
 1.83563531527095E-03
 1.68473642916669E-02
 6.39184391410295E-04
 1.25435596863551E-04
 3.45529586139955E-03
 0
 0
 2.01077997393379E-03
 7.32929792260419E-04
 3.36678560898465E-04
 2.057289097065E-04
 5.84914389783325E-04
 3.91879865409847E-04
 5.23585140685847E-04
 1.77147527990129E-03
 6.74883797738764E-04
 6.76704387326411E-04
 5.1806720080103E-04
 0
 7.10667857252009E-03

 4.06230211407968E-03
 1.83563531527095E-03
 1.68473642916669E-02
 6.39184391410295E-04
 1.25435596863551E-04
 3.45529586139955E-03
 0
 0
 2.01077997393379E-03
 7.32929792260419E-04
 3.36678560898465E-04
 0
 8.35591985405624E-05
 9.63384701854148E-05
 4.17795992702812E-05
 1.77147527990129E-03
 6.74883797738764E-04
 6.76704387326411E-04
 5.18067200802371E-05
 0
 1.20964741659939E-04

 0
 0
 0
 0
 0
 0
 0
 0
 0
 0
 0
 2.057289097065E-04
 5.01355191242763E-04
 2.95541395224432E-04
 4.81805541415565E-04

 0
 0
 0
 0
 0
 0
 0
 0
 0
 0
 0
 0
 0
 0
 0
 0
 0
 0
 4.66260480720793E-04
 0
 6.98571383086015E-03

 8.37064799025971E-02
 .119920009148154
 8.03201307970995E-02
 .185713233318262
 3.27250598068423E-02
 .038595345449381
 .174339828811592
 .046117909954626
 8.42759870395467E-02
 5.73725628594487E-02
 5.19628098266242E-02
 9.89784643363445E-02
 5.42092596114976E-02
 8.81591882641562E-02
 5.46998672752499E-02
 1.18383143501697E-02
 2.34336300060113E-02
 1.39396249530104E-02
 8.22163679591205E-02
 9.04391778781886E-02
 .117042531735999

 0
 7.86700849402147E-04
 3.14904005452059E-04
 0
 0
 0
 0
 0
 0
 0
 0
 0
 3.34236794162861E-04
 0
 4.75288991473675E-04
 1.18098351993589E-03
 1.61972111456838E-03
 1.52258487148199E-03
 1.03613440160474E-04
 0
 2.41929483319877E-04

 0
 7.86700849402147E-04
 3.14904005452059E-04
 0
 0
 0
 0
 0
 0
 0
 0
 0
 3.34236794162861E-04
 0
 4.75288991473675E-04
 1.18098351993589E-03
 1.61972111456838E-03
 1.52258487148199E-03
 1.03613440160474E-04
 0
 2.41929483319877E-04

 0
 7.86700849402147E-04
 3.14904005452059E-04
 0
 0
 0
 0
 0
 0
 0
 0
 0
 3.34236794162861E-04
 0
 4.75288991473675E-04
 1.18098351993589E-03
 1.61972111456838E-03
 1.52258487148199E-03
 1.03613440160474E-04
 0
 2.41929483319877E-04

 8.56453095478978E-03
 1.01147252066199E-02
 8.77232586615892E-03
 1.34033053505353E-02
 1.61274338824121E-03
 8.81453025873127E-03
 9.44698579769796E-03
 2.27132397302373E-03
 1.03411541516951E-02
 1.74507093395458E-03
 1.08218108860221E-03
 9.25780093677637E-03
 2.77535909437658E-03
 7.93193373188661E-03
 2.13059080152523E-03
 4.90319050687191E-03
 1.14971275543158E-02
 6.70662383867878E-03
 1.72072320266087E-03
 7.65976138468421E-04
 1.23124826332531E-03

 8.56453095478978E-03
 1.01147252066199E-02
 8.77232586615892E-03
 1.34033053505353E-02
 1.61274338824121E-03
 8.81453025873127E-03
 9.44698579769796E-03
 2.27132397302373E-03
 1.03411541516951E-02
 1.74507093395458E-03
 1.08218108860221E-03
 9.25780093677637E-03
 2.77535909437658E-03
 7.93193373188661E-03
 2.13059080152523E-03
 4.90319050687191E-03
 1.14971275543158E-02
 6.70662383867878E-03
 1.72072320266087E-03
 7.65976138468421E-04
 1.23124826332531E-03

 8.56453095478978E-03
 1.01147252066199E-02
 8.77232586615892E-03
 1.34033053505353E-02
 1.61274338824121E-03
 8.81453025873127E-03
 9.44698579769796E-03
 2.27132397302373E-03
 1.03411541516951E-02
 1.74507093395458E-03
 1.08218108860221E-03
 9.25780093677637E-03
 2.77535909437658E-03
 7.93193373188661E-03
 2.13059080152523E-03
 4.90319050687191E-03
 1.14971275543158E-02
 6.70662383867878E-03
 1.72072320266087E-03
 7.65976138468421E-04
 1.23124826332531E-03

 1.45602226311371E-04
 0
 5.24840009086042E-04
 0
 1.39372885403945E-04
 2.74229830271262E-04
 0
 8.12223132117616E-05
 0
 0
 4.67609112359462E-05
 2.28587677451667E-04
 0
 2.21336583376294E-04
 0
 8.2012744440016E-04
 3.67436734323705E-03
 1.50378752739202E-03
 1.38151253547233E-03
 2.26955892879066E-04
 6.04823708298727E-04

 1.45602226311371E-04
 0
 5.24840009086042E-04
 0
 1.39372885403945E-04
 2.74229830271262E-04
 0
 8.12223132117616E-05
 0
 0
 4.67609112359462E-05
 2.28587677451667E-04
 0
 2.21336583376294E-04
 0
 8.2012744440016E-04
 3.67436734323705E-03
 1.50378752739202E-03
 1.38151253547233E-03
 2.26955892879066E-04
 6.04823708298727E-04

 1.45602226311371E-04
 0
 5.24840009086042E-04
 0
 1.39372885403945E-04
 2.74229830271262E-04
 0
 8.12223132117616E-05
 0
 0
 4.67609112359462E-05
 2.28587677451667E-04
 0
 2.21336583376294E-04
 0
 8.2012744440016E-04
 3.67436734323705E-03
 1.50378752739202E-03
 1.38151253547233E-03
 2.26955892879066E-04
 6.04823708298727E-04

 0
 0
 0
 8.83665057253427E-05
 0
 7.9615112014347E-05
 0
 1.41484029465357E-04
 0
 0
 4.07272452700177E-05
 0
 0
 0
 0
 0
 0
 0
 5.11382462726766E-04
 5.93013784622337E-04
 2.34125306438927E-04

 0
 0
 0
 8.83665057253427E-05
 0
 7.9615112014347E-05
 0
 1.41484029465357E-04
 0
 0
 4.07272452700177E-05
 0
 0
 0
 0
 0
 0
 0
 3.10840320480976E-04
 0
 0

 0
 0
 0
 8.83665057253427E-05
 0
 7.9615112014347E-05
 0
 1.41484029465357E-04
 0
 0
 4.07272452700177E-05
 0
 0
 0
 0
 0
 0
 0
 3.10840320480976E-04
 0
 0

 0
 0
 0
 0
 0
 0
 0
 0
 0
 0
 0
 0
 0
 0
 0
 0
 0
 0
 2.00542142245791E-04
 5.93013784622337E-04
 2.34125306438927E-04

 0
 0
 0
 0
 0
 0
 0
 0
 0
 0
 0
 0
 0
 0
 0
 0
 0
 0
 2.00542142245791E-04
 5.93013784622337E-04
 2.34125306438927E-04

 .074996346721496
 .109018583092132
 7.07080609164025E-02
 .172221561462001
 3.09729435331971E-02
 2.94269702483641E-02
 .164892843013894
 4.36238796389251E-02
 7.39348328878516E-02
 5.56274919254941E-02
 5.07931405815161E-02
 8.94920757221165E-02
 5.10996637229582E-02
 8.00059179488933E-02
 .052093987482251
 1.0220049691748E-03
 4.51653003101469E-03
 3.31845420708985E-03
 7.81882959976192E-02
 8.88532320622187E-02
 .114730404974617

 0
 3.93350424701615E-04
 9.44712016356176E-04
 2.73936167748562E-04
 0
 0
 0
 4.38600491342607E-04
 0
 0
 0
 9.25780093678121E-03
 0
 8.09708539506607E-03
 2.31127945793761E-04

 0
 0
 4.72356008178088E-04
 0
 0
 0
 0
 0
 0
 0
 0

 0
 3.93350424701615E-04
 4.72356008178088E-04
 2.73936167748562E-04
 0
 0
 0
 4.38600491342607E-04
 0
 0
 0
 .001234373458239
 0
 9.06202139675744E-04
 2.31127945793761E-04

 0
 0
 0
 0
 0
 0
 0
 0
 0
 0
 0
 8.02342747854221E-03
 0
 7.19088325539032E-03
 0

 .074996346721496
 .10862523266743
 6.97633489000463E-02
 .171947625294252
 3.09729435331971E-02
 2.94269702483641E-02
 .164892843013894
 4.31852791475825E-02
 7.39348328878516E-02
 5.56274919254941E-02
 5.07931405815161E-02
 8.02342747853352E-02
 5.10996637229582E-02
 7.19088325538273E-02
 5.18628595364572E-02
 1.0220049691748E-03
 4.51653003101469E-03
 3.31845420708985E-03
 7.81882959976192E-02
 8.88532320622187E-02
 .114730404974617

 .074996346721496
 .10862523266743
 6.97633489000463E-02
 .171947625294252
 3.09729435331971E-02
 2.94269702483641E-02
 .164892843013894
 4.31852791475825E-02
 7.39348328878516E-02
 5.56274919254941E-02
 5.07931405815161E-02
 8.02342747853352E-02
 5.10996637229582E-02
 7.19088325538273E-02
 5.18628595364572E-02
 1.0220049691748E-03
 4.51653003101469E-03
 3.31845420708985E-03
 7.81882959976192E-02
 8.88532320622187E-02
 .114730404974617

 0
 0
 0
 0
 0
 0
 0
 0
 0
 0
 0
 0
 0
 0
 0
 6.6430322996362E-04
 2.12588396287536E-03
 8.88174508367738E-04
 0
 0
 0

 0
 0
 0
 0
 0
 0
 0
 0
 0
 0
 0
 0
 0
 0
 0
 6.6430322996362E-04
 2.12588396287536E-03
 8.88174508367738E-04
 0
 0
 0

 0
 0
 0
 0
 0
 0
 0
 0
 0
 0
 0
 0
 0
 0
 0
 6.6430322996362E-04
 2.12588396287536E-03
 8.88174508367738E-04
 0
 0
 0

 0
 0
 0
 0
 0
 0
 0
 0
 0
 0
 0
 0
 0
 0
 0
 3.24770467982327E-03
 0
 0
 3.10840320480827E-04
 0
 0

 0
 0
 0
 0
 0
 0
 0
 0
 0
 0
 0
 0
 0
 0
 0
 3.24770467982327E-03
 0
 0
 3.10840320480827E-04
 0
 0

 0
 0
 0
 0
 0
 0
 0
 0
 0
 0
 0
 0
 0
 0
 0
 3.24770467982327E-03
 0
 0
 3.10840320480827E-04
 0
 0

 1.91321325372822E-03
 2.00608716597385E-03
 4.50312727795702E-03
 1.82624111832149E-03
 2.50871193726583E-04
 8.76164307715985E-03
 1.14508918760024E-04
 6.94450777959957E-04
 7.57393790182375E-03
 6.18918491241145E-04
 6.22855337662351E-04
 1.54296682279593E-04
 3.76016393432363E-03
 2.69825281510978E-04
 3.08194728528796E-03
 1.5648031639173E-03
 1.62646995254809E-03
 2.01319555229865E-03
 .010661822992493
 4.5652177852702E-03
 7.99576942373185E-03

 1.91321325372822E-03
 2.00608716597385E-03
 4.50312727795702E-03
 1.82624111832149E-03
 2.50871193726583E-04
 8.76164307715985E-03
 1.14508918760024E-04
 6.94450777959957E-04
 7.57393790182375E-03
 6.18918491241145E-04
 6.22855337662351E-04
 1.54296682279593E-04
 3.76016393432363E-03
 2.69825281510978E-04
 3.08194728528796E-03
 1.5648031639173E-03
 1.62646995254809E-03
 2.01319555229865E-03
 .010661822992493
 4.5652177852702E-03
 7.99576942373185E-03

 4.71751213247544E-04
 2.36010254820644E-04
 1.98389523434251E-03
 5.47872335497125E-04
 0
 6.91059172283308E-03
 0
 0
 0
 4.88619861508291E-05
 7.57526762021285E-05
 0
 2.50677595621229E-04
 0
 1.71564386969367E-04
 8.85737639953191E-05
 4.04930278643259E-05
 1.52258487148588E-04
 7.70883994792426E-03
 3.49285119141499E-03
 7.33046334459958E-03

 4.71751213247544E-04
 2.36010254820644E-04
 1.98389523434251E-03
 5.47872335497125E-04
 0
 6.91059172283308E-03
 0
 0
 0
 4.88619861508291E-05
 7.57526762021285E-05
 0
 2.50677595621229E-04
 0
 1.71564386969367E-04
 8.85737639953191E-05
 4.04930278643259E-05
 1.52258487148588E-04
 7.70883994792426E-03
 3.49285119141499E-03
 7.33046334459958E-03

 1.44146204048068E-03
 1.77007691115321E-03
 2.51923204361452E-03
 1.27836878282436E-03
 2.50871193726583E-04
 1.85105135432677E-03
 1.14508918760024E-04
 6.94450777959957E-04
 7.57393790182375E-03
 5.70056505090315E-04
 5.47102661460223E-04
 1.54296682279593E-04
 3.5094863387024E-03
 2.69825281510978E-04
 2.9103828983186E-03
 1.47622939992199E-03
 1.58597692468377E-03
 1.86093706515006E-03
 2.95298304456875E-03
 1.0723665938552E-03
 6.65306079132271E-04

 3.9312601103989E-04
 5.9002563705161E-04
 9.44712016354225E-04
 8.21808503243424E-04
 0
 8.63823965351587E-04
 0
 1.09650122835878E-04
 3.41832595569022E-03
 0
 0
 1.54296682279593E-04
 2.00542076497258E-03
 7.71483411397964E-05
 1.69609421986673E-03
 0
 1.01232569660815E-04
 0
 2.64214272408673E-03
 7.65976138468169E-04
 5.44341337472332E-04

 1.04833602944079E-03
 1.1800512741016E-03
 1.57452002726029E-03
 4.56560279580937E-04
 2.50871193726583E-04
 9.87227388975184E-04
 1.14508918760024E-04
 5.84800655124079E-04
 4.15561194613353E-03
 5.70056505090315E-04
 5.47102661460223E-04
 0
 1.50406557372982E-03
 0
 1.21428867845187E-03
 1.47622939992199E-03
 1.48474435502295E-03
 1.86093706515006E-03
 3.10840320482018E-04
 3.06390455387033E-04
 1.20964741659939E-04

 0
 0
 0
 0
 0
 0
 0
 0
 0
 0
 0
 0
 0
 1.92676940371182E-04
 0

 3.35802200974539E-04
 4.11474139783637E-04
 5.32453359088516E-04
 1.42380911930933E-04
 0
 7.09317953364939E-04
 7.08302590266729E-05
 5.77105909662516E-05
 1.67564997827932E-04
 2.54489511202235E-04
 2.63030125702197E-05
 9.52498133898709E-04
 5.02447561825957E-03
 5.17536982743104E-04
 3.94270817083662E-03
 1.08356293313439E-03
 4.5158501426043E-03
 2.64034650379409E-03
 3.85982573988093E-03
 2.34815635890935E-03
 7.45904083651869E-03

 1.28893774111439E-04
 0
 3.09741644706303E-04
 0
 0
 2.42760833354553E-04
 0
 0
 0
 0
 0
 0
 4.1094687806834E-05
 0
 9.63269982621658E-05
 2.1780433769299E-04
 2.65528051568586E-04
 1.24802038646384E-04
 7.64361443805311E-05
 0
 0

 1.28893774111439E-04
 0
 3.09741644706303E-04
 0
 0
 2.42760833354553E-04
 0
 0
 0
 0
 0
 0
 4.1094687806834E-05
 0
 9.63269982621658E-05
 2.1780433769299E-04
 2.65528051568586E-04
 1.24802038646384E-04
 7.64361443805311E-05
 0
 0

 1.28893774111439E-04
 0
 3.09741644706303E-04
 0
 0
 2.42760833354553E-04
 0
 0
 0
 0
 0
 0
 4.1094687806834E-05
 0
 9.63269982621658E-05
 2.1780433769299E-04
 2.65528051568586E-04
 1.24802038646384E-04
 7.64361443805311E-05
 0
 0

 0
 4.05515901754242E-05
 0
 2.82408420359343E-05
 0
 2.54440048705645E-05
 7.08302590266729E-05
 0
 0
 0
 0

 0
 4.05515901754242E-05
 0
 2.82408420359343E-05
 0
 2.54440048705645E-05
 7.08302590266729E-05
 0
 0
 0
 0

 0
 4.05515901754242E-05
 0
 2.82408420359343E-05
 0
 2.54440048705645E-05
 7.08302590266729E-05
 0
 0
 0
 0

 0
 1.63896010292001E-04
 9.84075017037683E-05
 1.14140069894999E-04
 0
 5.14180931759324E-05
 0
 0
 1.67564997827932E-04
 2.54489511202235E-04
 2.63030125702197E-05
 4.88606160551644E-04
 4.59575591972902E-04
 2.44303080275822E-04
 4.70546072854512E-04
 4.42868819976065E-04
 1.64695139438662E-03
 1.67101548176103E-03
 3.25087168502944E-03
 1.83834273232264E-03
 4.22649276160657E-03

 0
 1.63896010292001E-04
 9.84075017037683E-05
 1.14140069894999E-04
 0
 5.14180931759324E-05
 0
 0
 1.67564997827932E-04
 2.54489511202235E-04
 2.63030125702197E-05
 3.85741705698394E-04
 4.17795992702621E-04
 1.92870852849197E-04
 4.49656273219371E-04
 4.42868819976065E-04
 1.49318040249648E-03
 1.63889343805873E-03
 3.25087168502944E-03
 1.83834273232264E-03
 4.20352477268374E-03

 0
 1.63896010292001E-04
 9.84075017037683E-05
 1.14140069894999E-04
 0
 5.14180931759324E-05
 0
 0
 1.67564997827932E-04
 2.54489511202235E-04
 2.63030125702197E-05
 6.42902842831636E-05
 0
 3.21451421415818E-05
 0
 7.38114699960993E-05
 9.44837316833494E-04
 1.05735060519934E-03
 1.37287808212345E-03
 1.07236659385579E-03
 3.44749513730738E-03

 0
 0
 0
 0
 0
 0
 0
 0
 0
 0
 0
 3.2145142141523E-04
 4.17795992702621E-04
 1.60725710707615E-04
 4.49656273219371E-04
 2.76793012484842E-04
 5.48343085662989E-04
 5.81542832859394E-04
 9.06617601403404E-04
 0
 1.13404445306301E-04

 0
 0
 0
 0
 0
 0
 0
 0
 0
 0
 0
 0
 0
 0
 0
 9.22643374951241E-05
 0
 0
 9.71376001502583E-04
 7.65976138466848E-04
 6.42625190070054E-04

 0
 0
 0
 0
 0
 0
 0
 0
 0
 0
 0
 1.0286445485325E-04
 4.17795992702812E-05
 5.1432227426625E-05
 2.08897996351406E-05
 0
 1.5377099189014E-04
 3.21220437022954E-05
 0
 0
 2.29679889228392E-05

 0
 0
 0
 0
 0
 0
 0
 0
 0
 0
 0
 1.0286445485325E-04
 4.17795992702812E-05
 5.1432227426625E-05
 2.08897996351406E-05
 0
 1.5377099189014E-04
 3.21220437022954E-05
 0
 0
 2.29679889228392E-05

 2.069084268631E-04
 2.07026539316212E-04
 1.24304212678444E-04
 0
 0
 3.89695021963888E-04
 0
 5.77105909662516E-05
 0
 0
 0
 0
 4.21534167373771E-03
 0
 3.0565118774965E-03
 0
 5.06162848303491E-04
 0
 0
 4.59585683080549E-04
 2.26808890612463E-03

 2.069084268631E-04
 2.07026539316212E-04
 1.24304212678444E-04
 0
 0
 3.89695021963888E-04
 0
 5.77105909662516E-05
 0
 0
 0
 0
 4.21534167373771E-03
 0
 3.0565118774965E-03
 0
 5.06162848303491E-04
 0
 0
 4.59585683080549E-04
 2.26808890612463E-03

 2.069084268631E-04
 2.07026539316212E-04
 1.24304212678444E-04
 0
 0
 3.89695021963888E-04
 0
 5.77105909662516E-05
 0
 0
 0
 0
 1.58322691972123E-03
 0
 1.27819860889989E-03
 0
 5.06162848303491E-04
 0
 0
 4.59585683080549E-04
 1.81447112489908E-03

 0
 0
 0
 0
 0
 0
 0
 0
 0
 0
 0
 0
 2.63211475401648E-03
 0
 1.77831326859661E-03
 0
 0
 0
 0
 0
 4.53617781225552E-04

 0
 0
 0
 0
 0
 0
 0
 0
 0
 0
 0
 3.08593364558944E-04
 2.14866510532613E-04
 1.95584598073221E-04
 2.72524645118691E-04
 0
 4.64674090245981E-04
 0
 2.54787147935592E-04
 5.02279435061672E-05
 1.7847256966306E-04

 0
 0
 0
 0
 0
 0
 0
 0
 0
 0
 0
 3.08593364558944E-04
 2.14866510532613E-04
 1.95584598073221E-04
 2.72524645118691E-04
 0
 4.64674090245981E-04
 0
 2.54787147935592E-04
 5.02279435061672E-05
 1.7847256966306E-04

 0
 0
 0
 0
 0
 0
 0
 0
 0
 0
 0
 3.08593364558944E-04
 2.14866510532613E-04
 1.95584598073221E-04
 2.72524645118691E-04
 0
 4.64674090245981E-04
 0
 2.54787147935592E-04
 5.02279435061672E-05
 1.7847256966306E-04

 0
 0
 0
 0
 0
 0
 0
 0
 0
 0
 0
 1.55298608788122E-04
 9.35971542095152E-05
 7.76493043940608E-05
 4.67985771047576E-05

 0
 0
 0
 0
 0
 0
 0
 0
 0
 0
 0
 1.55298608788122E-04
 9.35971542095152E-05
 7.76493043940608E-05
 4.67985771047576E-05

 0
 0
 0
 0
 0
 0
 0
 0
 0
 0
 0
 3.50674277908165E-05
 2.84860904115554E-05
 1.75337138954083E-05
 1.42430452057777E-05

 0
 0
 0
 0
 0
 0
 0
 0
 0
 0
 0
 1.20231180997305E-04
 6.51110637979599E-05
 6.01155904986526E-05
 3.25555318989799E-05

 0
 0
 0
 0
 0
 0
 0
 0
 0
 0
 0
 0
 0
 0
 0
 1.89800922846749E-04
 3.76006687311764E-04
 1.76728601154742E-04
 1.72072320266645E-04
 0
 1.65246477446362E-04

 0
 0
 0
 0
 0
 0
 0
 0
 0
 0
 0
 0
 0
 0
 0
 1.89800922846749E-04
 3.76006687311764E-04
 1.76728601154742E-04
 1.72072320266645E-04
 0
 1.65246477446362E-04

 0
 0
 0
 0
 0
 0
 0
 0
 0
 0
 0
 0
 0
 0
 0
 1.89800922846749E-04
 3.76006687311764E-04
 1.45008082998725E-04
 1.33217280206579E-04
 0
 5.18420321399737E-05

 0
 0
 0
 0
 0
 0
 0
 0
 0
 0
 0
 0
 0
 0
 0
 0
 0
 3.17205181560167E-05
 3.88550400600661E-05
 0
 1.13404445306388E-04

 0
 0
 0
 0
 0
 0
 0
 0
 0
 0
 0
 0
 0
 0
 0
 0
 8.4360474717103E-05
 0
 6.47584001001102E-05
 0
 0

 0
 0
 0
 0
 0
 0
 0
 0
 0
 0
 0
 0
 0
 0
 0
 0
 8.4360474717103E-05
 0
 6.47584001001102E-05
 0
 0

 0
 0
 0
 0
 0
 0
 0
 0
 0
 0
 0
 0
 0
 0
 0
 0
 8.4360474717103E-05
 0
 6.47584001001102E-05
 0
 0

 0
 0
 0
 0
 0
 0
 0
 0
 0
 0
 0
 0
 0
 0
 0
 2.33088852618591E-04
 1.17216659607075E-03
 6.6780038223193E-04
 4.09000421686082E-05
 0
 6.2074012167806E-04

 0
 0
 0
 0
 0
 0
 0
 0
 0
 0
 0
 0
 0
 0
 0
 2.33088852618591E-04
 1.17216659607075E-03
 6.6780038223193E-04
 4.09000421686082E-05
 0
 6.2074012167806E-04

 0
 0
 0
 0
 0
 0
 0
 0
 0
 0
 0
 0
 0
 0
 0
 2.33088852618591E-04
 1.17216659607075E-03
 6.6780038223193E-04
 4.09000421686082E-05
 0
 6.2074012167806E-04

 0
 0
 0
 0
 0
 0
 0
 0
 0
 0
 0
 0
 0
 0
 0
 9.42274085056587E-05
 0
 0
 9.92043576002638E-05
 6.51894585931107E-05
 0

 0
 0
 0
 0
 0
 0
 0
 0
 0
 0
 0
 0
 0
 0
 0
 9.42274085056587E-05
 0
 0
 9.92043576002638E-05
 6.51894585931107E-05
 0

 0
 0
 0
 0
 0
 0
 0
 0
 0
 0
 0
 0
 0
 0
 0
 9.42274085056587E-05
 0
 0
 9.92043576002638E-05
 6.51894585931107E-05
 0

 0
 0
 0
 0
 0
 0
 0
 0
 0
 0
 0
 0
 0
 0
 0
 9.42274085056587E-05
 0
 0
 9.92043576002638E-05
 6.51894585931107E-05
 0

 1.33830131417973E-03
 1.25537369585276E-03
 1.20601534002584E-03
 2.79764596849788E-03
 8.8071802052999E-04
 1.10275399832334E-03
 2.04654237783511E-03
 3.73277013908987E-04
 1.54017189492801E-03
 3.63865854312967E-04
 8.86467487471606E-04
 1.90408671749273E-03
 8.85371933471815E-03
 2.36637409125711E-03
 7.13154839473333E-03
 1.69609335309969E-03
 4.52315736780753E-03
 5.45322950425816E-03
 2.6454495360045E-03
 5.93224073197195E-03
 1.30101440232231E-02

 1.33830131417973E-03
 1.25537369585276E-03
 1.20601534002584E-03
 2.79764596849788E-03
 8.8071802052999E-04
 1.10275399832334E-03
 2.04654237783511E-03
 3.73277013908987E-04
 1.54017189492801E-03
 3.63865854312967E-04
 8.86467487471606E-04
 1.90408671749273E-03
 8.85371933471815E-03
 2.36637409125711E-03
 7.13154839473333E-03
 1.69609335309969E-03
 4.52315736780753E-03
 5.45322950425816E-03
 2.6454495360045E-03
 5.93224073197195E-03
 1.30101440232231E-02

 1.33830131417973E-03
 1.25537369585276E-03
 1.20601534002584E-03
 2.79764596849788E-03
 8.8071802052999E-04
 1.10275399832334E-03
 2.04654237783511E-03
 3.73277013908987E-04
 1.54017189492801E-03
 3.63865854312967E-04
 8.86467487471606E-04
 1.90408671749273E-03
 8.85371933471815E-03
 2.36637409125711E-03
 7.13154839473333E-03
 1.69609335309969E-03
 4.52315736780753E-03
 5.45322950425816E-03
 2.6454495360045E-03
 5.93224073197195E-03
 1.30101440232231E-02

 1.33830131417973E-03
 1.25537369585276E-03
 1.20601534002584E-03
 2.79764596849788E-03
 8.8071802052999E-04
 1.10275399832334E-03
 2.04654237783511E-03
 3.73277013908987E-04
 1.54017189492801E-03
 3.63865854312967E-04
 8.86467487471606E-04
 1.90408671749273E-03
 8.85371933471815E-03
 2.36637409125711E-03
 7.13154839473333E-03
 1.69609335309969E-03
 4.52315736780753E-03
 5.45322950425816E-03
 2.6454495360045E-03
 5.93224073197195E-03
 1.30101440232231E-02

 1.33830131417973E-03
 1.25537369585276E-03
 1.20601534002584E-03
 2.79764596849788E-03
 8.8071802052999E-04
 1.10275399832334E-03
 2.04654237783511E-03
 3.73277013908987E-04
 1.54017189492801E-03
 3.63865854312967E-04
 8.86467487471606E-04
 1.90408671749273E-03
 8.85371933471815E-03
 2.36637409125711E-03
 7.13154839473333E-03
 1.69609335309969E-03
 4.52315736780753E-03
 5.45322950425816E-03
 2.6454495360045E-03
 5.93224073197195E-03
 1.30101440232231E-02

 7.66786972019108E-02
 .122850263951707
 .106405212349953
 .103945449035982
 4.15198605919915E-02
 6.92130892107451E-02
 9.75492194408934E-02
 4.84695032169995E-02
 .150499091541258
 5.34919894869242E-02
 4.15251540463254E-02
 4.74043889889612E-02
 9.76949307413863E-02
 4.63693346579022E-02
 9.98894694071257E-02
 1.03367976467932E-02
 .016296802106105
 1.33816006430525E-02
 2.18226707157251E-02
 7.47592711144877E-03
 1.02699065669215E-02

 7.66786972019108E-02
 .122850263951707
 .106405212349953
 .103945449035982
 4.15198605919915E-02
 6.92130892107451E-02
 9.75492194408934E-02
 4.84695032169995E-02
 .150499091541258
 5.34919894869242E-02
 4.15251540463254E-02
 4.74043889889612E-02
 9.76949307413863E-02
 4.63693346579022E-02
 9.98894694071257E-02
 1.03367976467932E-02
 .016296802106105
 1.33816006430525E-02
 2.18226707157251E-02
 7.47592711144877E-03
 1.02699065669215E-02

 7.66786972019108E-02
 .122850263951707
 .106405212349953
 .103945449035982
 4.15198605919915E-02
 6.92130892107451E-02
 9.75492194408934E-02
 4.84695032169995E-02
 .150499091541258
 5.34919894869242E-02
 4.15251540463254E-02
 4.74043889889612E-02
 9.76949307413863E-02
 4.63693346579022E-02
 9.98894694071257E-02
 1.03367976467932E-02
 .016296802106105
 1.33816006430525E-02
 2.18226707157251E-02
 7.47592711144877E-03
 1.02699065669215E-02

 0
 0
 0
 1.36968083874281E-04
 0
 2.46806847243966E-04
 0
 1.09650122835878E-04
 0
 0
 6.31272301685274E-05
 0
 3.76016393433218E-04
 0
 3.0357216961349E-04
 0
 2.02465139321047E-04
 3.80646217871471E-04
 3.10840320480976E-04
 0
 1.81447112489908E-04

 0
 0
 0
 1.36968083874281E-04
 0
 2.46806847243966E-04
 0
 1.09650122835878E-04
 0
 0
 6.31272301685274E-05
 0
 3.76016393433218E-04
 0
 3.0357216961349E-04
 0
 2.02465139321047E-04
 3.80646217871471E-04
 3.10840320480976E-04
 0
 1.81447112489908E-04

 2.35875606623772E-03
 7.60477487754723E-03
 6.61298411447502E-03
 3.2872340129737E-03
 0
 1.79346308997408E-02
 5.03839242543351E-03
 0
 1.34051998262622E-03
 0
 0
 .000411457819413
 .038270112931669
 3.9840585007733E-04
 3.56221832657942E-02

 2.35875606623772E-03
 7.60477487754723E-03
 6.61298411447502E-03
 3.2872340129737E-03
 0
 1.79346308997408E-02
 5.03839242543351E-03
 0
 1.34051998262622E-03
 0
 0
 .000411457819413
 .038270112931669
 3.9840585007733E-04
 3.56221832657942E-02

 7.43199411356731E-02
 .11524548907416
 9.97922282354781E-02
 .100521246939134
 4.15198605919915E-02
 5.10316514637604E-02
 9.25108270154599E-02
 4.83598530941636E-02
 .149158571558632
 5.34919894869242E-02
 4.14620268161569E-02
 4.69929311695482E-02
 5.90488014162842E-02
 4.59709288078248E-02
 6.39637139717179E-02
 1.03367976467932E-02
 .016094336966784
 .013000954425181
 2.15118303952442E-02
 7.47592711144877E-03
 1.00884594544316E-02

 3.4000087441279E-03
 7.76069756844079E-03
 7.02150822966373E-03
 5.84890736543739E-03
 1.93238622194948E-03
 1.86772749265759E-03
 2.50681687014882E-03
 4.14892356674337E-03
 2.93465185385381E-03
 2.77324786259608E-03
 8.32596981681128E-03
 3.33614448172702E-04
 1.69376753798128E-03
 7.9170540907254E-04
 2.09622401652677E-03
 2.39388551338012E-04
 3.83042155473038E-04
 6.17264137087294E-04
 1.26016346140876E-04
 0
 0

 0
 1.78795647591643E-04
 0
 0
 0
 0
 0
 0
 0
 0
 5.73883910622976E-05

 7.09199323915452E-02
 .107305995858128
 9.27707200058144E-02
 9.46723395736968E-02
 3.95874743700421E-02
 4.91639239711028E-02
 9.00040101453111E-02
 4.42109295274203E-02
 .146223919704778
 5.07187416243281E-02
 3.30786686082833E-02
 4.66593167213755E-02
 5.73550338783029E-02
 4.51792233987523E-02
 6.18674899551912E-02
 1.00974090954552E-02
 1.57112948113109E-02
 1.23836902880937E-02
 2.13858140491033E-02
 7.47592711144877E-03
 1.00884594544316E-02

 .117954183562235
 9.31339078485827E-02
 .118275976297602
 5.17511076903983E-02
 .144501807586611
 .189398546213196
 3.67001084625178E-02
 .147858621888833
 7.67363907553183E-02
 .167438828999888
 .304246946399792
 .125372483380579
 .184848614521446
 .178129834744326
 .18640670821912
 8.50677191703989E-02
 9.79087669566398E-02
 .138058268520653
 4.31647115867862E-02
 .108002635523934
 .214024429478598

 .117954183562235
 9.31339078485827E-02
 .118275976297602
 5.17511076903983E-02
 .144501807586611
 .189398546213196
 3.67001084625178E-02
 .147858621888833
 7.67363907553183E-02
 .167438828999888
 .304246946399792
 .125372483380579
 .184848614521446
 .178129834744326
 .18640670821912
 8.50677191703989E-02
 9.79087669566398E-02
 .138058268520653
 4.31647115867862E-02
 .108002635523934
 .214024429478598

 .117954183562235
 9.31339078485827E-02
 .118275976297602
 5.17511076903983E-02
 .144501807586611
 .189398546213196
 3.67001084625178E-02
 .147858621888833
 7.67363907553183E-02
 .167438828999888
 .304246946399792
 .125372483380579
 .184848614521446
 .178129834744326
 .18640670821912
 8.50677191703989E-02
 9.79087669566398E-02
 .138058268520653
 4.31647115867862E-02
 .108002635523934
 .214024429478598

 .117954183562235
 9.31339078485827E-02
 .118275976297602
 5.17511076903983E-02
 .144501807586611
 .189398546213196
 3.67001084625178E-02
 .147858621888833
 7.67363907553183E-02
 .167438828999888
 .304246946399792
 .125372483380579
 .184848614521446
 .178129834744326
 .18640670821912
 8.50677191703989E-02
 9.79087669566398E-02
 .138058268520653
 4.31647115867862E-02
 .108002635523934
 .214024429478598

 1.84277817674822E-03
 9.83376061753022E-04
 1.62372377810974E-03
 5.99235366947152E-04
 5.87979360297893E-04
 4.62762838582117E-04
 2.14704222674602E-04
 8.90907248040944E-04
 0
 4.58081120162762E-04
 4.73454226262652E-04
 9.64354264247454E-05
 4.7002049179009E-04
 2.28852344810356E-04
 5.96147661197137E-04
 4.84387771849004E-03
 1.58175890094841E-03
 2.5376414524786E-03
 1.21422000187916E-03
 1.05321719039329E-03
 9.07235562449539E-04

 2.45703756899931E-04
 2.45844015438002E-04
 1.47611252555652E-04
 2.56815157263924E-04
 3.5278761617825E-04
 0
 0
 2.05593980316988E-04
 0
 7.63468533606705E-05
 1.18363556565826E-04

 7.37111270699287E-03
 7.74408648628665E-03
 7.0853401226762E-03
 2.12300530004811E-02
 1.35235252868224E-02
 2.85370417125851E-03
 1.53513519212338E-02
 1.52139545434532E-02
 1.88510622556293E-02
 1.45822489918679E-02
 1.38090815993328E-02
 2.89306279274765E-04
 7.83367486317772E-04
 9.57508981826655E-04
 1.04173109070371E-03
 5.53586024969949E-03
 2.53081424151673E-03
 4.12366736027487E-03
 2.13702720330643E-03
 5.36183296927344E-03
 6.86096894104665E-03

 1.83458805152003E-03
 1.44228489057042E-03
 1.41706802453036E-03
 3.01329784522966E-03
 2.91010584722774E-02
 6.58151592651255E-04
 1.14508918760024E-03
 4.19594470051066E-02
 .029625491615993
 5.40739313400302E-02
 9.17449078448013E-02
 4.53246504195545E-03
 2.23259733600329E-02
 8.04654073209997E-03
 .020697014444008
 1.23172890555696E-02
 .013350045123997
 1.53844513056216E-02
 8.15955841262505E-03
 2.34580192405679E-02
 1.34384267688156E-02

 .106537148991623
 8.24724723790966E-02
 .107854621867174
 .026480496215634
 .100348477490737
 .185269673331177
 .01923749835165
 8.91089998245101E-02
 2.58720356646462E-02
 9.75610990142224E-02
 .196956958126028
 5.14322274264368E-04
 1.05284590161118E-02
 5.46176547688957E-04
 8.96227664075438E-03
 6.6430322996362E-04
 4.04930278642676E-04
 3.80646217871471E-04
 1.55420160240264E-04
 0
 0

 1.22851878450219E-04
 2.45844015438002E-04
 1.47611252555652E-04
 1.71210104842498E-04
 5.87979360297893E-04
 1.54254279527479E-04
 7.51464779359113E-04
 4.79719287404702E-04
 2.38780121904984E-03
 6.87121680244142E-04
 1.14418104680195E-03
 .11993995435866
 .150740794167193
 .1683507561379
 .155109538382456
 2.36196703986839E-03
 2.90200033027319E-03
 3.72187413030012E-03
 .023468444196303
 .056580104094807
 .173221510057352

 0
 0
 0
 0
 0
 0
 0
 0
 0
 0
 0
 0
 0
 0
 0
 5.93444218768078E-02
 7.71392180812617E-02
 .111909988054106
 8.03004161243228E-03
 2.15494620288923E-02
 1.95962881489344E-02

 1.90946919647923E-03
 9.74010575450535E-04
 2.2943006111457E-03
 3.44376896597783E-03
 3.54803545413214E-03
 1.48084108346161E-03
 2.09387737160363E-03
 8.85555277758285E-03
 4.05986051880863E-03
 4.04856456677184E-03
 1.11344409782565E-02
 2.38057738374019E-03
 1.22712651571134E-02
 2.09862283933399E-03
 1.04500209000312E-02
 2.40414502272694E-02
 2.38908864399093E-02
 2.35879815011726E-02
 1.42986547421226E-02
 3.9684858983484E-03
 1.14052470707942E-03

 1.90946919647923E-03
 9.74010575450535E-04
 2.2943006111457E-03
 3.44376896597783E-03
 3.54803545413214E-03
 1.48084108346161E-03
 2.09387737160363E-03
 8.85555277758285E-03
 4.05986051880863E-03
 4.04856456677184E-03
 1.11344409782565E-02
 2.38057738374019E-03
 1.22712651571134E-02
 2.09862283933399E-03
 1.04500209000312E-02
 2.40414502272694E-02
 2.38908864399093E-02
 2.35879815011726E-02
 1.42986547421226E-02
 3.9684858983484E-03
 1.14052470707942E-03

 2.62084007360468E-04
 0
 0
 0
 0
 3.29075796325288E-04
 0
 0
 0
 0
 0

 2.62084007360468E-04
 0
 0
 0
 0
 3.29075796325288E-04
 0
 0
 0
 0
 0

 2.62084007360468E-04
 0
 0
 0
 0
 3.29075796325288E-04
 0
 0
 0
 0
 0

 1.12321717439891E-03
 1.87309726048388E-04
 1.34958859479082E-03
 5.21783176662851E-04
 1.79193709805072E-03
 8.22689490811035E-04
 4.90752508970857E-04
 4.17714753659625E-03
 1.91502854660889E-03
 2.09408512074405E-03
 5.41090544300173E-03
 7.34746106091955E-04
 8.59466042132197E-03
 5.04999439025142E-04
 6.60860966858874E-03
 9.2791562280665E-03
 .011473024561544
 8.70048497995268E-03
 8.80714241362881E-03
 2.91800433701656E-04
 1.72806773799912E-04

 1.12321717439891E-03
 1.87309726048388E-04
 1.34958859479082E-03
 5.21783176662851E-04
 1.79193709805072E-03
 8.22689490811035E-04
 4.90752508970857E-04
 4.17714753659625E-03
 1.91502854660889E-03
 2.09408512074405E-03
 5.41090544300173E-03
 7.34746106091955E-04
 8.59466042132197E-03
 5.04999439025142E-04
 6.60860966858874E-03
 9.2791562280665E-03
 .011473024561544
 8.70048497995268E-03
 8.80714241362881E-03
 2.91800433701656E-04
 1.72806773799912E-04

 1.12321717439891E-03
 1.87309726048388E-04
 1.34958859479082E-03
 5.21783176662851E-04
 1.79193709805072E-03
 8.22689490811035E-04
 4.90752508970857E-04
 4.17714753659625E-03
 1.91502854660889E-03
 2.09408512074405E-03
 5.41090544300173E-03
 7.34746106091955E-04
 8.59466042132197E-03
 5.04999439025142E-04
 6.60860966858874E-03
 9.2791562280665E-03
 .011473024561544
 8.70048497995268E-03
 8.80714241362881E-03
 2.91800433701656E-04
 1.72806773799912E-04

 5.24168014719854E-04
 7.86700849402147E-04
 9.44712016354875E-04
 2.92198578931498E-03
 1.75609835608142E-03
 3.29075796325288E-04
 1.60312486263277E-03
 4.6784052409866E-03
 2.14483197219974E-03
 1.95447944602778E-03
 5.72353553525478E-03
 1.64583127764824E-03
 3.67660473579147E-03
 1.59362340030885E-03
 3.84141123144251E-03
 1.47622939992029E-02
 1.24178618783653E-02
 1.48874965212199E-02
 5.49151232849381E-03
 3.67668546464674E-03
 9.67717933279509E-04

 5.24168014719854E-04
 7.86700849402147E-04
 9.44712016354875E-04
 2.92198578931498E-03
 1.75609835608142E-03
 3.29075796325288E-04
 1.60312486263277E-03
 4.6784052409866E-03
 2.14483197219974E-03
 1.95447944602778E-03
 5.72353553525478E-03
 0
 3.34236794162861E-04
 0
 6.29374288669798E-04
 1.47622939992029E-02
 1.24178618783653E-02
 1.48874965212199E-02
 5.49151232849381E-03
 3.67668546464674E-03
 9.67717933279509E-04

 5.24168014719854E-04
 7.86700849402147E-04
 9.44712016354875E-04
 2.92198578931498E-03
 1.75609835608142E-03
 3.29075796325288E-04
 1.60312486263277E-03
 4.6784052409866E-03
 2.14483197219974E-03
 1.95447944602778E-03
 5.72353553525478E-03
 0
 3.34236794162861E-04
 0
 6.29374288669798E-04
 1.47622939992029E-02
 1.24178618783653E-02
 1.48874965212199E-02
 5.49151232849381E-03
 3.67668546464674E-03
 9.67717933279509E-04

 0
 0
 0
 0
 0
 0
 0
 0
 0
 0
 0
 1.64583127764824E-03
 3.34236794162861E-03
 1.59362340030885E-03
 3.21203694277271E-03

 0
 0
 0
 0
 0
 0
 0
 0
 0
 0
 0
 1.64583127764824E-03
 3.34236794162861E-03
 1.59362340030885E-03
 3.21203694277271E-03

 3.30225849273605E-02
 3.81549911959949E-02
 3.82608366623783E-02
 5.67047867235813E-02
 2.38954812025002E-02
 3.69798926120817E-02
 6.33806865335486E-02
 .028618682060099
 .121987318418634
 2.85842618981664E-02
 3.13742333936309E-02
 .195802489812673
 2.08062404365877E-02
 .15888349653362
 1.66435747547199E-02
 1.52789742892218E-02
 1.70070717029913E-02
 1.50989666422442E-02
 2.19142425938929E-02
 4.90224728619664E-03
 9.25380273697748E-03

 3.30225849273605E-02
 3.81549911959949E-02
 3.82608366623783E-02
 5.67047867235813E-02
 2.38954812025002E-02
 3.69798926120817E-02
 6.33806865335486E-02
 .028618682060099
 .121987318418634
 2.85842618981664E-02
 3.13742333936309E-02
 .195802489812673
 2.08062404365877E-02
 .15888349653362
 1.66435747547199E-02
 1.52789742892218E-02
 1.70070717029913E-02
 1.50989666422442E-02
 2.19142425938929E-02
 4.90224728619664E-03
 9.25380273697748E-03

 3.30225849273605E-02
 3.81549911959949E-02
 3.82608366623783E-02
 5.67047867235813E-02
 2.38954812025002E-02
 3.69798926120817E-02
 6.33806865335486E-02
 .028618682060099
 .121987318418634
 2.85842618981664E-02
 3.13742333936309E-02
 .195802489812673
 2.08062404365877E-02
 .15888349653362
 1.66435747547199E-02
 1.52789742892218E-02
 1.70070717029913E-02
 1.50989666422442E-02
 2.19142425938929E-02
 4.90224728619664E-03
 9.25380273697748E-03

 3.30225849273605E-02
 3.81549911959949E-02
 3.82608366623783E-02
 5.67047867235813E-02
 2.38954812025002E-02
 3.69798926120817E-02
 6.33806865335486E-02
 .028618682060099
 .121987318418634
 2.85842618981664E-02
 3.13742333936309E-02
 .195802489812673
 2.08062404365877E-02
 .15888349653362
 1.66435747547199E-02
 1.52789742892218E-02
 1.70070717029913E-02
 1.50989666422442E-02
 2.19142425938929E-02
 4.90224728619664E-03
 9.25380273697748E-03

 0
 0
 0
 0
 0
 8.22689490814919E-05
 0
 0
 1.34051998262622E-04
 0
 0
 0
 0
 0
 0

 3.30225849273605E-02
 3.81549911959949E-02
 3.82608366623783E-02
 5.67047867235813E-02
 2.38954812025002E-02
 3.68976236630002E-02
 6.33806865335486E-02
 .028618682060099
 .121853266420371
 2.85842618981664E-02
 3.13742333936309E-02
 .195802489812673
 2.08062404365877E-02
 .15888349653362
 1.66435747547199E-02
 1.52789742892218E-02
 1.70070717029913E-02
 1.50989666422442E-02
 2.19142425938929E-02
 4.90224728619664E-03
 9.25380273697748E-03

 4.91407513799863E-04
 2.45844015438509E-04
 0
 0
 2.35191744119157E-04
 0
 0
 1.37062653544848E-04
 0
 0
 7.89090377106592E-05
 3.85741705699687E-04
 2.66344945347957E-03
 7.34774747643793E-04
 2.05399955734397E-03
 5.53586024969153E-04
 1.39194783283402E-03
 7.93012953899505E-04
 2.91412800450775E-04
 0
 1.13404445306518E-04

 4.91407513799863E-04
 2.45844015438509E-04
 0
 0
 2.35191744119157E-04
 0
 0
 1.37062653544848E-04
 0
 0
 7.89090377106592E-05
 3.85741705699687E-04
 2.66344945347957E-03
 7.34774747643793E-04
 2.05399955734397E-03
 5.53586024969153E-04
 1.39194783283402E-03
 7.93012953899505E-04
 2.91412800450775E-04
 0
 1.13404445306518E-04

 4.91407513799863E-04
 2.45844015438509E-04
 0
 0
 2.35191744119157E-04
 0
 0
 1.37062653544848E-04
 0
 0
 7.89090377106592E-05
 3.85741705699687E-04
 2.66344945347957E-03
 7.34774747643793E-04
 2.05399955734397E-03
 5.53586024969153E-04
 1.39194783283402E-03
 7.93012953899505E-04
 2.91412800450775E-04
 0
 1.13404445306518E-04

 4.91407513799863E-04
 2.45844015438509E-04
 0
 0
 2.35191744119157E-04
 0
 0
 1.37062653544848E-04
 0
 0
 7.89090377106592E-05
 3.85741705699687E-04
 2.66344945347957E-03
 7.34774747643793E-04
 2.05399955734397E-03
 5.53586024969153E-04
 1.39194783283402E-03
 7.93012953899505E-04
 2.91412800450775E-04
 0
 1.13404445306518E-04

 4.91407513799863E-04
 2.45844015438509E-04
 0
 0
 2.35191744119157E-04
 0
 0
 1.37062653544848E-04
 0
 0
 7.89090377106592E-05
 3.85741705699687E-04
 2.66344945347957E-03
 7.34774747643793E-04
 2.05399955734397E-03
 5.53586024969153E-04
 1.39194783283402E-03
 7.93012953899505E-04
 2.91412800450775E-04
 0
 1.13404445306518E-04

 0
 0
 0
 0
 0
 0
 0
 0
 0
 0
 0
 0
 3.85657839418685E-04
 0
 3.7061964724333E-04

 0
 0
 0
 0
 0
 0
 0
 0
 0
 0
 0
 0
 3.85657839418685E-04
 0
 3.7061964724333E-04

 0
 0
 0
 0
 0
 0
 0
 0
 0
 0
 0
 0
 3.85657839418685E-04
 0
 3.7061964724333E-04

 0
 0
 0
 0
 0
 0
 0
 0
 0
 0
 0
 0
 3.85657839418685E-04
 0
 3.7061964724333E-04

 0
 0
 0
 0
 0
 0
 0
 0
 0
 0
 0
 0
 3.85657839418685E-04
 0
 3.7061964724333E-04

 0
 0
 0
 0
 0
 0
 0
 0
 0
 0
 0
 0
 1.25338797810844E-04
 0
 6.26693989054218E-05

 0
 0
 0
 0
 0
 0
 0
 0
 0
 0
 0
 0
 1.25338797810844E-04
 0
 6.26693989054218E-05

 0
 0
 0
 0
 0
 0
 0
 0
 0
 0
 0
 0
 1.25338797810844E-04
 0
 6.26693989054218E-05

 0
 0
 0
 0
 0
 0
 0
 0
 0
 0
 0
 0
 1.25338797810844E-04
 0
 6.26693989054218E-05

 0
 0
 0
 0
 0
 0
 0
 0
 0
 0
 0
 0
 1.25338797810844E-04
 0
 6.26693989054218E-05

 0
 0
 0
 0
 0
 0
 0
 0
 0
 0
 0
 1.31316325344574E-04
 0
 6.56581626722872E-05
 4.91761586795237E-05

 0
 0
 0
 0
 0
 0
 0
 0
 0
 0
 0
 1.31316325344574E-04
 0
 6.56581626722872E-05
 4.91761586795237E-05

 0
 0
 0
 0
 0
 0
 0
 0
 0
 0
 0
 1.31316325344574E-04
 0
 6.56581626722872E-05
 4.91761586795237E-05

 0
 0
 0
 0
 0
 0
 0
 0
 0
 0
 0
 1.31316325344574E-04
 0
 6.56581626722872E-05
 4.91761586795237E-05

 0
 0
 0
 0
 0
 0
 0
 0
 0
 0
 0
 1.31316325344574E-04
 0
 6.56581626722872E-05
 4.91761586795237E-05
